# Iron-Catalyzed Oxidative α-Amination of Ketones with Primary and Secondary Sulfonamides

**DOI:** 10.1021/acs.joc.3c00210

**Published:** 2023-02-22

**Authors:** Fubin Song, So Hyun Park, Christine Wu, Alexandra E. Strom

**Affiliations:** Department of Chemistry, Smith College, Northampton, Massachusetts 01063, United States

## Abstract

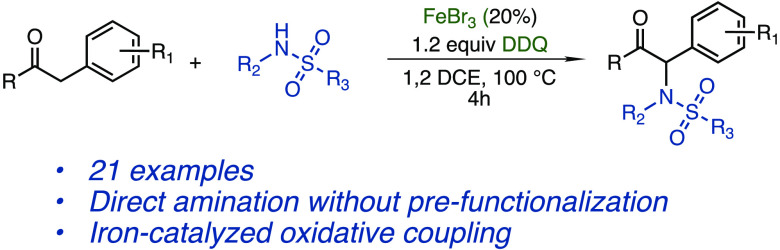

We report the iron-catalyzed α-amination of ketones with sulfonamides. Using an oxidative coupling approach, ketones can be directly coupled with free sulfonamides, without the need for prefunctionalization of either substrate. Primary and secondary sulfonamides are both competent coupling partners, with yields from 55% to 88% for deoxybenzoin-derived substrates.

Bond formation at the α-carbon of carbonyl compounds is a classic approach to the synthesis of complex molecules.^1^ Carbonyl compounds are ubiquitous in nature, in chemical catalogs, and as synthetic intermediates.^2^ 1,2-Aminoalcohols^3^ and α-aminocarbonyls are prevalent moieties in bioactive compounds, and many pharmaceutically relevant molecules contain nitrogen and, specifically, sulfonamides.^4^ While these functional group arrangements can be achieved through alternate pathways such as aziridine or epoxide opening, ketones are an abundant and attractive starting point for amination.

α-Amination of ketones is often accomplished through the use of prefunctionalized ketones or amines.^4^ Preactivation of ketone coupling partners is common, through either α-halogenation^4^ or other umpolung activation,^5,6^ for the formation of an electrophilic center at the α-carbon or through formation of the silyl enol ether or enolate with the use of a strong base.^7^ Electrophilic nitrogen sources such as azodicarboxylates,^8^*N*-nitrosamines,^9^ chloramine derivatives,^10^ or iodine(III) derivatives (such as PHINTs)^11^ require the synthesis of an aminating reagent and can be costly. These prior reaction steps also limit the range of functional groups for incorporation, due to their commercial availability or reactivity in formation of the aminating reagent. Because of these limitations and the desirability of this transformation, protocols for the oxidative amination of ketones have been developed using NIS^12^ or copper(II) bromide in air.^13^ These methods are limited to nucleophilic (often cyclic, secondary) amines. In addition to these amination reactions with alkyl amines, the use of TBHP in the presence of TBAI^14^ has been disclosed for the imidation of ketones.

Iron catalysis is an exciting approach to α-functionalization with multiple mechanistic possibilities. Three prior examples of iron-catalyzed oxidative coupling reactions are shown in Scheme 1. These examples showcase the range of mechanistic possibilities as well as the range of functionalization reactions that are possible with simple iron salts under oxidative conditions. The oxidative α-arylation in Scheme 1a is proposed to go through the formation of a carbocation at the benzylic α-carbon of the ketone.^15^ The addition of TEMPO to the α-carbon of arylacetic acids is shown in Scheme 1b, which is proposed to be formed through the addition of TEMPO to an iron enolate with concomitant reduction of the iron catalyst.^16^ The iron-catalyzed α-amination of thiohydantoins is shown in Scheme 1c, which is proposed to proceed through an α-carbocation, stabilized by the adjacent nitrogen in the ring.^17^ To the best of our knowledge, this α-amination of thiohydantoins is the only previous report of iron-catalyzed direct α-amination of carbonyl compounds. This work comprises the identification of conditions for the α-amination of ketones directly with free sulfonamides in the presence of iron halide salts and quinone-based oxidants (Scheme 1d).

**Scheme 1:**
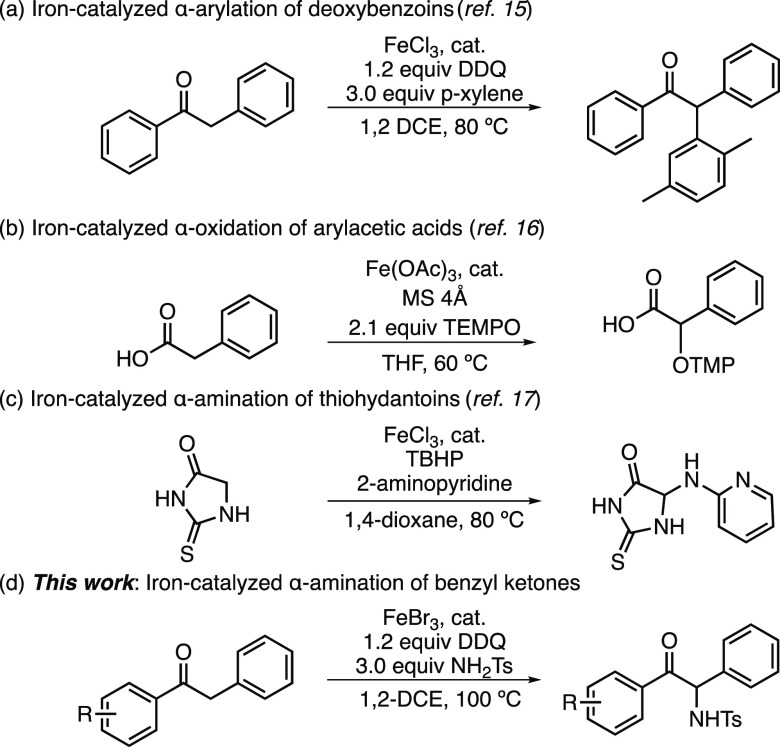
Examples of Direct Iron-Catalyzed α-Functionalization of Carbonyl Compounds

Many pitfalls are possible with this approach to α-amination, including overoxidation,^18,19^ fragmentation,^20^ or homocoupling.^21^ However, in the course of our investigations of iron-catalyzed reactions, we found that C–N bond formation at the α-carbon of the ketone was possible under oxidative conditions using a sulfonamide as the nitrogen source (Scheme 2). Under these conditions, α-arylation, as shown in Scheme 1a, was not observed. The combination of deoxybenzoin **1a** and *p*-toluenesulfonamide **2a** in the presence of iron(III) chloride and 2,3-dichloro-5,6-benzoquinone (DDQ) resulted in C–N bond formation at the α-carbon of the ketone in moderate yield (42%), and the main byproduct was varying amounts of overoxidation to form benzil (**4**). Deoxybenzoin slowly oxidizes to form benzil upon storage or exposure to air and light, but we have not observed this issue with substituted deoxybenzoins. For this reason, we chose the fluorinated analogue of deoxybenzoin (**1b**) for further study of reaction conditions.

**Scheme 2:**
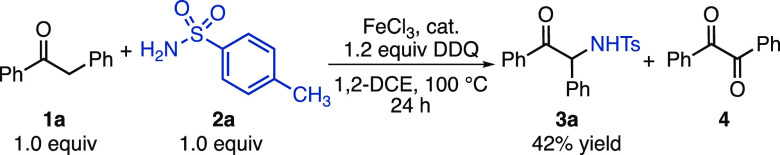
Amination of Deoxybenzoin with *p*-Toluenesulfonamide

Our initial reaction optimization is shown in Table 1. Changing the source of iron to iron(III) bromide combined with the use of the fluoroketone brought the yield to 47% (entry 1). Shortening the reaction time to 4 h resulted in higher yields, indicating that some of the product is decomposing or undergoing further oxidation under the reaction conditions (entry 2). Increasing the number of equivalents of sulfonamide increased the yield to 77% (entry 3). Interestingly, increasing the number of equivalents of ketone relative to sulfonamide resulted in a low yield (entry 4). The use of iron(III) chloride resulted in results similar to those of iron(III) bromide (entry 5), although reactions with iron(III) bromide were more consistent. While both iron halide salts are hygroscopic, iron(III) chloride is available in only kilogram quantities. It may be that more rigorous exclusion of water from iron(III) chloride would result in more consistent results. Iron(III) triflate (entry 6) formed the product in a lower yield but was still competent in the absence of halides. Decreasing the catalyst loading to 10% still resulted in synthetically useful yields (entry 7). Weaker oxidants were less effective in the reaction (entries 8 and 9), and non-quinone oxidants gave little to no product (see the Supporting Information). The addition of water decreased the yield significantly (entry 10), which supports our hypothesis for the disparate results with iron(III) chloride and iron(III) bromide. Ancillary ligands resulted in little or no product formation [entries 11 and 12 (see the Supporting Information for additional results)]. Although the reaction is sensitive to water, exposure of the reaction mixture to air before heating resulted in relatively high yields being maintained (entry 13).

**Table 1:**
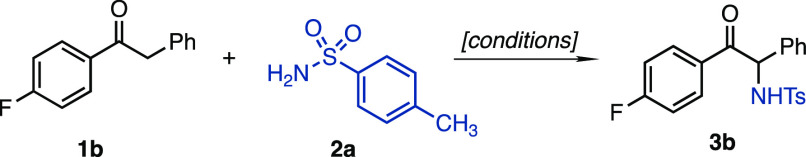
Optimization of the Reaction Conditions^a^

entry	**1b** (equiv)	**2a** (equiv)	catalyst	oxidant	additive (equiv)	time (h)	yield^b^
1	1.0	1.2	FeBr_3_	DDQ	–	24	47
2	1.0	1.2	FeBr_3_	DDQ	–	4	56
3	1.0	3.0	FeBr_3_	DDQ	–	4	77
4	1.2	1.0	FeBr_3_	DDQ	–	4	34^c^
5	1.0	3.0	FeCl_3_	DDQ	–	4	74
6	1.0	3.0	Fe(OTf)_3_	DDQ	–	4	36
7	1.0	3.0	FeBr_3_	DDQ	–	4	72^d^
8	1.0	3.0	FeBr_3_	BQ	–	4	6
9	1.0	3.0	FeBr_3_	*p*-chloranil	–	4	37
10	1.0	3.0	FeBr_3_	DDQ	water (0.3)	4	14
11	1.0	3.0	FeBr_3_	DDQ	pyridine (0.2)	4	0
12	1.0	3.0	FeBr_3_	DDQ	2,2′-bipyridine (0.2)	4	0
13	1.0	3.0	FeBr_3_	DDQ	–	4	72^e^

a Conditions: **1b** (0.200 mmol), **2b** (0.600 mmol), oxidant (0.240 mmol), and catalyst (20 mol %, 0.0400 mmol) in 1,2-dichloroethane (1.0 mL) at 100 °C.

b Yield determined by ^1^H NMR integration of **3b** relative to the internal standard (ethylene carbonate) relative to limiting reagent **1b**.

c Yield based on limiting reagent **2a** (0.200 mmol).

d With 10 mol % catalyst (0.0200 mmol).

e Reaction mixture exposed to air prior to heating.

With the optimized conditions in hand, the scope (Figure 1) of the sulfonamide was examined with 4-chlorophenyl benzyl ketone and 4-fluorophenyl benzyl ketone (**3c** and **3d**, respectively). *ortho-*Substitution on the sulfonamide aryl group was tolerated (**3e**). Lower yields were observed for electron-poor (**3f** and **3g**) and heterocyclic (**3h**) aromatic groups on the sulfonamide. Simple methane sulfonamide also formed the product, albeit in a yield lower than those of reactions with electron-rich aryl sulfonamides (**3i**). Secondary sulfonamides were also successful, with a higher yield for *N*-methyl- than *N*-(*n*-hexyl)-sulfonamide (**3j** and **3k**).

**Figure 1:**
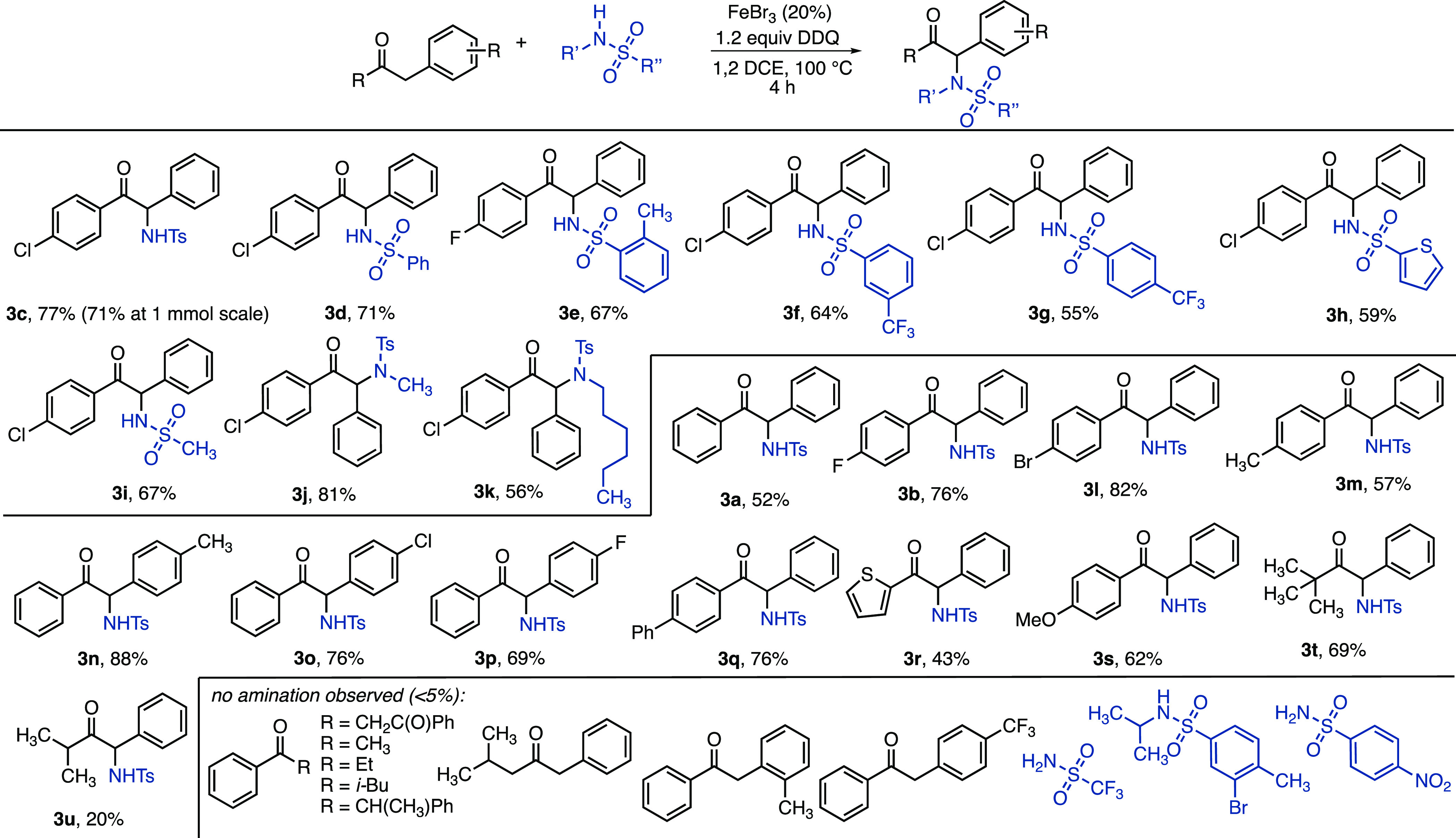
Scope of sulfonamides and ketones for α-amination.

Variations in the ketone starting material revealed that unsubstituted deoxybenzoin gave a moderate (55%) yield (**3a**). Various halogen substituents on either aromatic ring are tolerated (**3b**, **3l**, **3o**, and **3p**) as well as methyl substituents (**3m** and **3n**). A thienyl ketone can form the amination product in a reduced yield (**3r**), and phenyl and methoxy substituents do not hinder reactivity (**3q** and **3s**). Non-aromatic ketones are also reactive, with a higher yield for the *tert*-butyl ketone than for the isopropyl ketone (**3t** and **3u**). Ketones without α-aryl groups were unreactive for α-amination under these reaction conditions, including acetophenone, propiophenone, isovalerophenone, and the diketone dibenzoylmethane. Sterically hindered [α-methyldeoxybenzoin and 1-phenyl-2-(2-methylphenyl)ethanone] and electron-deficient ketones were also unsuccessful. Although various sulfonamides are well-tolerated under these conditions, trifluoromethane sulfonamide, *p*-nitrobenzenesulfonamide, and the more hindered *N*-isopropyl(3-bromo-4-methylbenzene)sulfonamide were not competent nitrogen sources under these reaction conditions.

In conclusion, we have demonstrated a direct α-amination reaction of benzyl ketones with free primary and secondary sulfonamides under oxidative conditions. This direct approach negates the need for prefunctionalization of either starting material, and a range of sulfonamide substituents are tolerated. Studies are ongoing to elucidate the mechanism of this reaction.

## Experimental Section

### General

All reagents and solvents were purchased from various commercial sources and used without further purification, including 1,2-dichloroethane (anhydrous, SureSeal), chloroform D (99.8 atom % D, Millipore Sigma), deoxybenzoin (combiblocks), iron tribromide (anhydrous, Strem), iron trifluoride (anhydrous, Strem), iron(II) chloride (anhydrous, Strem), iron(II) trifluoromethanesulfonate (Strem), iron(II) acetate (anhydrous, Strem), iron(III) acetylacetonate (Strem), iron(III) trifluoromethanesulfonate (Alfa Aesar), benzyl 4-bromophenyl ketone (Acros Organics), benzyl 4-chlorophenyl ketone (TCI America), benzyl 4-fluorophenyl ketone (Matrix Scientific), *tert*-butyl magnesium chloride solution (1.0 M in THF, Millipore Sigma), benzylmagnesium chloride solution (2.0 M in THF, Millipore Sigma), thionyl chloride (1.0 M in DCM, Alfa Aesar), 4-methylbenzyl phenyl ketone (TCI America), silver hexafluorophosphate (TCI America), tetrahydrofuran (anhydrous, SureSeal, Millipore Sigma), 1,4-dioxane (anhydrous, SureSeal, Millipore Sigma), toluene (anhydrous, SureSeal, Millipore Sigma), sodium *tert*-butoxide (Thermo Scientific), *p*-toluenesulfonyl chloride (Thermo Scientific), isovalerophenone (TCI America), *N*-methyl-*p*-toluenesulfonamide (TCI America), and isobutyraldehyde (Thermo Scientific). 4-Methylphenyl benzyl ketone,^22^ 4-methoxyl benzyl ketone,^23^*tert*-butyl benzyl ketone,^24^ biphenyl benzyl ketone,^25^ 4-fluorobenzyl phenyl ketone,^26^ and *N*-(*n*-hexyl)-*p*-toluenesulfonamide^27^ were synthesized via literature procedures. Iron-catalyzed reaction mixtures were assembled in a nitrogen-filled glovebox, and the vials were tightly sealed and removed from the glovebox for heating on an aluminum heating block with temperature control. Other reactions were conducted using standard Schlenk techniques under a nitrogen atmosphere to exclude moisture and air, unless otherwise noted. Compounds were purified via flash chromatography using either Silicycle siliaFlash P60 silica or Biotage Sfär columns. Thin layer chromatography was performed with SiliaPlate silica plates treated with F254 indicator and visualized with UV light or staining with phosphomolybdic acid stain, *p*-anisaldehyde stain, or KMNO_4_ stain, as needed. NMR spectra were recorded on a Bruker 300 MHz or Bruker 500 MHz NMR spectrometer. Chemical shifts are reported in parts per million and referenced to a chloroform solvent as the internal standard. Data are reported as s (singlet), d (doublet), t (triplet), q (quartet), sept (septet), m (multiplet), and br (broad), and coupling constants are reported in hertz, followed by integration.

### Isopropyl Benzyl Ketone^28^

To a solution of isobutyraldehyde (0.456 mL, 5.0 mmol, 1.00 equiv) in THF (10 mL, 0.5 M) was added a benzylmagnesium chloride solution (3.00 mL, 2 M in THF, 6.0 mmol, 1.20 equiv) dropwise at 0 °C. The reaction mixture was stirred at 0 °C for 30 min, allowed to warm to room temperature, and stirred for 16 h. The reaction was quenched with a saturated aqueous NH_4_Cl solution and extracted with dichloromethane (2 × 25 mL). The combined organic layers were dried with anhydrous MgSO_4_, and the crude material was used without purification in the next step.

2-Methyl-3-hydroxy-4-phenylbutane (0.615 g, 3.7 mmol, 1.0 equiv) and Dess-Martin periodinane (2.38 g, 5.62 mmol, 1.5 equiv) were added to DCM (50 mL, 0.075 M) at 0 °C, and the mixture was stirred for 30 min. The reaction mixture was allowed to warm to room temperature and stirred for 24 h. H_2_O (1 mL) was added, and the reaction mixture was filtered through Celite, concentrated under reduced pressure, and purified via column chromatography (hexanes/EtOAc) to yield isopropyl benzyl ketone (163 mg, 20% yield over two steps, white solid). ^1^H NMR (500 MHz, chloroform-*d*): δ 7.38–7.32 (m, 2H), 7.31–7.26 (m, 1H), 7.23 (d, *J* = 7.5 Hz, 2H), 3.77 (s, 2H), 2.76 (sept, *J* = 6.9 Hz, 1H), 1.13 (d, *J* = 6.9 Hz, 6H). ^13^C{^1^H} NMR (126 MHz, CDCl_3_): δ 211.9, 134.4, 129.4, 128.6, 126.8, 47.7, 40.1, 18.3. 3.1.

### General Procedure for Table 1

To an oven-dried vial in the glovebox were added ketone (0.200 mmol, 1.00 equiv), DDQ (54.5 mg, 0.240 mmol, 1.20 equiv), *p*-toluenesulfonamide (103 mg, 0.600 mmol, 3.00 equiv), iron(III) bromide (11.8 mg, 0.0400 mmol, 0.200 equiv), and an oven-dried stir bar. 1,2-Dichloroethane (1.0 mL, 0.20 M, anhydrous) was added, and the vial was sealed with a PTFE-lined cap, removed from the glovebox, and heated at the listed temperature in an aluminum heating block for the indicated time. The reaction mixture was allowed to cool to room temperature and then opened to air, and 1 mL of saturated aqueous NH_4_Cl was added. The aqueous solution was extracted with DCM until the organic phase was clear, and the combined organic layers were filtered through a pad of silica, washing with 20% MeOH in DCM (10 mL). Ethylene carbonate (8.8 mg, 0.10 mmol, 0.5 equiv) was added, and the solvent was removed in vacuo. The crude solid was dissolved in CDCl_3_ (0.5 mL), and a portion of the CDCl_3_ solution was diluted further with CDCl_3_ for ^1^H NMR analysis.

### Scale-up to 1 mmol Scale

To an oven-dried 20 mL vial in the glovebox were added 1-(4-chlorophenyl)-2-phenylethan-1-one (231 mg, 1.00 mmol, 1.00 equiv), DDQ (272 mg, 1.20 mmol, 1.20 equiv), *p*-toluenesulfonamide (514 mg, 3.00 mmol, 3.0 equiv), iron(III) bromide (118 mg, 0.200 mmol, 0.200 equiv), and an oven-dried stir bar. 1,2-Dichloroethane (5.0 mL, 0.20 M, anhydrous) was added, and the vial was sealed with a PTFE-lined cap, removed from the glovebox, and heated in an oil bath (100 °C, 4 h). The reaction mixture was allowed to cool to room temperature and then opened to air, and 5 mL of saturated aqueous NH_4_Cl was added. The aqueous solution was extracted with DCM until the organic phase was clear; the combined organic layers were added to silica (5 g), and the solvent was removed in vacuo. The crude reaction mixture and silica were loaded onto a Biotage Sfär column and purified via flash chromatography (hexanes/ethyl acetate) to yield product **3c** (285 mg, 71% yield) as a white solid.

### General Procedure A for Isolated Yields

To an oven-dried 4 mL vial in the glovebox were added ketone (0.200 mmol, 1.00 equiv), DDQ (54.5 mg, 0.240 mmol, 1.20 equiv), sulfonamide (0.6 mmol, 3.0 equiv), iron(III) bromide (11.8 mg, 0.0400 mmol, 0.200 equiv), and an oven-dried stir bar. 1,2-Dichloroethane (1.0 mL, 0.20 M, anhydrous) was added, and the vial was sealed with a PTFE-lined cap, removed from the glovebox, and heated in an aluminum heating block (100 °C, 4 h). The reaction mixture was allowed to cool to room temperature and then opened to air, and 1–2 mL of saturated aqueous NH_4_Cl was added. The aqueous solution was extracted with DCM until the organic phase was clear, and the combined organic layers were filtered through a pad of silica, washing with 20% MeOH in DCM (10 mL). The solvent was removed in vacuo, and the crude material was purified via flash chromatography.

#### Compound **3a**(29)

General procedure A was followed, eluting with hexanes/ethyl acetate (0–40%) to afford 37.9 mg (52% yield) of **3a** as a white solid. ^1^H NMR (500 MHz, chloroform-*d*): δ 7.82 (d, *J* = 7.1 Hz, 2H), 7.55 (d, *J* = 8.4 Hz, 2H), 7.52 (t, *J* = 7.4 Hz, 1H), 7.38 (dd, *J* = 7.8, 7.8 Hz, 2H), 7.23–7.17 (m, 5H), 7.08 (d, *J* = 8.1 Hz, 2H), 6.26 (d, *J* = 7.4 Hz, 1H), 6.02 (d, *J* = 7.4 Hz, 1H), 2.32 (s, 3H). ^13^C{^1^H} NMR (126 MHz, CDCl_3_): δ 194.6, 143.1, 137.4, 135.7, 134.0, 133.8, 129.4, 129.1, 129.0, 128.7, 128.5, 128.2, 127.0, 61.7, 21.4. HRMS (ESI) *m*/*z*: calcd for C_21_H_19_NO_3_SNa [M + Na]^+^, 388.0983; found, 388.0976.

#### Compound **3b**

General procedure A was followed, eluting with hexanes/ethyl acetate (0–40%) to afford 58.4 mg (76% yield) of **3b** as a white solid. ^1^H NMR (500 MHz, chloroform-*d*): δ 7.84 (dd, *J* = 8.8, 5.4 Hz, 2H), 7.52 (d, *J* = 8.2 Hz, 2H), 7.21–7.12 (m, 5H), 7.05 (d, *J* = 8.0 Hz, 2H), 7.04–6.96 (m, 2H), 6.28 (d, *J* = 7.7 Hz, 1H), 5.96 (dd, *J* = 7.5, 2.5 Hz, 1H), 2.29 (s, 3H). ^13^C{^1^H} NMR (126 MHz, CDCl_3_): δ 193.0, 166.0 (d, *J* = 257.1 Hz), 143.2, 137.4, 135.5, 131.7 (d, *J* = 9.5 Hz), 130.2 (d, *J* = 2.9 Hz), 129.4, 129.2, 128.6, 128.1, 127.0, 116.0 (d, *J* = 22.0 Hz), 61.7, 21.4. ^19^F NMR (471 MHz, CDCl_3_): δ −102.7. HRMS (ESI) *m*/*z*: calcd for C_21_H_18_FNO_3_SNa [M + Na]^+^, 406.0884; found, 406.0882.

#### Compound **3c**(30)

General procedure A was followed, eluting with hexanes/ethyl acetate (0–40%) to afford 61.7 mg (77% yield) of **3c** as a white solid. ^1^H NMR (500 MHz, chloroform-*d*): δ 7.76 (d, *J* = 8.7 Hz, 2H), 7.54 (d, *J* = 8.2 Hz, 2H), 7.35 (d, *J* = 8.5 Hz, 2H), 7.23–7.14 (m, 5H), 7.09 (d, *J* = 8.0 Hz, 2H), 6.23 (d, *J* = 7.3 Hz, 1H), 5.97 (d, *J* = 7.4 Hz, 1H), 2.33 (s, 3H). ^13^C{^1^H} NMR (126 MHz, CDCl_3_): δ 193.4, 143.2, 140.5, 137.3, 135.3, 132.1, 130.3, 129.3, 129.2, 129.1, 128.6, 128.1, 126.9, 61.7, 21.4.

#### Compound **3d**

General procedure A was followed, eluting with hexanes/ethyl acetate (0–40%) to afford 54.6 mg (71% yield) of **3d** as a white solid. ^1^H NMR (500 MHz, chloroform-*d*): δ 7.77 (d, *J* = 8.7 Hz, 2H), 7.64 (dd, *J* = 8.5, 1.3 Hz, 2H), 7.41 (t, *J* = 7.4 Hz, 1H), 7.36 (d, *J* = 8.6 Hz, 2H), 7.32–7.26 (m, 2H), 7.21–7.14 (m, 5H), 6.25 (d, *J* = 6.9 Hz, 1H), 6.00 (d, *J* = 7.2 Hz, 1H). ^13^C{^1^H} NMR (126 MHz, CDCl_3_): δ 193.2, 140.6, 140.4, 135.1, 132.4, 132.0, 130.3, 129.24, 129.15, 128.76, 128.75, 128.1, 126.9, 61.9. HRMS (ESI) *m*/*z*: calcd for C_20_H_16_ClNO_3_SNa [M + Na]^+^, 408.0432; found, 408.0432.

#### Compound **3e**

General procedure A was followed, eluting with hexanes/ethyl acetate (0–40%) to afford 51.1 mg (67% yield) of **3e** as a white solid. ^1^H NMR (500 MHz, chloroform-*d*): δ 7.85 (dd, *J* = 9.0, 5.2 Hz, 2H), 7.79 (dd, *J* = 7.9, 1.5 Hz, 1H), 7.29 (m, 1H), 7.18–7.07 (m, 7H), 7.04 (m, 2H), 6.32 (d, *J* = 7.0 Hz, 1H), 5.93 (d, *J* = 7.0 Hz, 1H), 2.60 (s, 3H). ^13^C{^1^H} NMR (126 MHz, CDCl_3_): δ 192.9, 166.0 (d, *J* = 257.1 Hz), 138.1, 137.1, 135.2, 132.6, 132.3, 131.8 (d, *J* = 9.5 Hz), 130.1 (d, *J* = 3.1 Hz), 129.12, 129.10, 128.7, 127.9, 125.8, 116.0 (d, *J* = 22.0 Hz), 61.7, 20.2. ^19^F NMR (471 MHz, CDCl_3_): δ −102.72. HRMS (ESI) *m*/*z*: calcd for C_21_H_18_FNO_3_SNa [M + Na]^+^, 406.0884; found, 406.0881.

#### Compound **3f**

General procedure A was followed, eluting with hexanes/ethyl acetate (10–40%) to afford 57.9 mg (64% yield) of **3f** as a white solid. ^1^H NMR (500 MHz, chloroform-*d*): δ 7.84–7.75 (m, 4H), 7.62 (d, *J* = 7.8 Hz, 1H), 7.42 (dd, *J* = 7.9, 7.9 Hz, 1H), 7.37 (d, *J* = 8.7 Hz, 2H), 7.18–7.09 (m, 5H), 6.47 (d, *J* = 6.8 Hz, 1H), 6.09 (d, *J* = 6.5 Hz, 1H). ^13^C{^1^H} NMR (126 MHz, CDCl_3_): δ 192.5, 141.9, 140.8, 134.3, 131.6, 131.1 (q, *J* = 33.5 Hz), 130.4, 129.9, 129.4, 129.23, 129.18, 129.0, 128.8 (q, *J* = 3.5 Hz), 128.1, 124.0 (q, *J* = 3.8 Hz), 123.0 (q, *J* = 272.9 Hz), 62.1. ^19^F NMR (471 MHz, CDCl_3_): δ −62.86. HRMS (ESI) *m*/*z*: calcd for C_21_H_15_ClF_3_NO_3_SNa [M+ Na]^+^, 476.0305; found, 476.0310.

#### Compound **3g**

General procedure A was followed, eluting with hexanes/ethyl acetate (10–40%) to afford 50.0 mg (55% yield) of **3g** as a white solid. ^1^H NMR (300 MHz, chloroform-*d*): δ 7.78 (d, *J* = 8.5 Hz, 2H), 7.64 (d, *J* = 8.1 Hz, 2H), 7.47 (d, *J* = 8.2 Hz, 2H), 7.35 (d, *J* = 8.4 Hz, 2H), 7.19–7.01 (m, 5H), 6.32 (d, *J* = 6.5 Hz, 1H), 6.02 (d, *J* = 6.5 Hz, 1H). ^13^C{^1^H} NMR (126 MHz, CDCl_3_): δ 192.6, 144.1, 140.8, 134.4, 133.7 (q, *J* = 32.4 Hz), 131.6, 130.4, 129.21, 129.17 128.9, 128.2, 127.3, 125.7 (q, *J* = 3.7 Hz), 123.1 (q, *J* = 272.8 Hz), 62.0. ^19^F NMR (471 MHz, CDCl_3_): δ −63.27. HRMS (ESI) *m*/*z*: calcd for C_21_H_15_ClF_3_NO_3_SNa [M + Na]^+^, 476.0305; found, 476.0308.

#### Compound **3h**

General procedure A was followed, eluting with hexanes/ethyl acetate (0–40%) to afford 45.9 mg (59% yield) of **3h** as a white solid. ^1^H NMR (500 MHz, chloroform-*d*): δ 7.81 (d, *J* = 8.7 Hz, 2H), 7.42 (dd, *J* = 5.0, 1.3 Hz, 1H), 7.37 (d, *J* = 8.6 Hz, 2H), 7.34 (dd, *J* = 3.7, 1.3 Hz, 1H), 7.27–7.20 (m, 5H), 6.86 (dd, *J* = 5.0, 3.8 Hz, 1H), 6.38 (d, *J* = 7.4 Hz, 1H), 6.05 (d, *J* = 7.4 Hz, 1H). ^13^C{^1^H} NMR (126 MHz, CDCl_3_): δ 193.1, 141.4, 140.6, 135.1, 132.2, 131.9 (2), 130.3, 129.3, 129.2, 128.8, 128.1, 127.1, 62.1. HRMS (ESI) *m*/*z*: calcd for C_18_H_14_ClNO_3_S_2_ [M + H]^+^, 392.0176; found, 392.0179.

#### Compound **3i**

General procedure A was followed, eluting with hexanes/ethyl acetate (10–40%) to afford 43.6 mg (67% yield) of **3i** as a white solid. ^1^H NMR (300 MHz, chloroform-*d*): δ 7.89 (d, *J* = 8.5 Hz, 1H), 7.46–7.30 (m, 7H), 6.10 (d, *J* = 6.1 Hz, 1H), 6.02 (d, *J* = 6.3 Hz, 1H), 2.60 (s, 3H). ^13^C{^1^H} NMR (75 MHz, CDCl_3_): δ 193.1, 140.7, 135.9, 131.9, 130.5, 129.7, 129.3, 129.2, 128.2, 62.2, 42.3. HRMS (ESI) *m*/*z*: calcd for C_15_H_14_ClNO_3_SNa [M + Na]^+^, 346.0275; found, 346.0275.

#### Compound **3j**

General procedure A was followed, eluting with hexanes/ethyl acetate (0–15%) to afford 61.6 mg (81% yield) of **3j** as a white solid. ^1^H NMR (500 MHz, chloroform-*d*): δ 7.75 (d, *J* = 8.6 Hz, 2H), 7.65 (d, *J* = 8.3 Hz, 2H), 7.39–7.32 (m, 5H), 7.26 (d, *J* = 8.0 Hz, 2H), 7.25–7.19 (m, 2H), 6.75 (s, 1H), 2.82 (s, 3H), 2.44 (s, 3H). ^13^C{^1^H} NMR (126 MHz, CDCl_3_): δ 195.6, 143.3, 140.0, 136.4, 133.8, 133.6, 130.0, 129.7, 129.5, 129.2, 129.1, 128.9, 127.3, 64.5, 31.4, 21.5. HRMS (ESI) *m*/*z*: calcd for C_22_H_20_ClNO_3_SNa [M + Na]^+^, 436.0745; found, 436.0744.

#### Compound **3k**

General procedure A was followed, eluting with hexanes/ethyl acetate (0–15%) to afford 54.0 mg (56% yield) of **3k** as a white solid. ^1^H NMR (300 MHz, chloroform-*d*): δ 7.79–7.49 (m, 4H), 7.43–7.31 (m, 5H), 7.30–7.16 (m, 4H), 6.64 (s, 1H), 3.42 (ddd, *J* = 15.9, 10.8, 5.0 Hz, 1H), 3.21 (ddd, *J* = 15.6, 11.4, 5.1 Hz, 1H), 2.42 (s, 3H), 1.55–1.35 (m, 2H), 1.15–0.82 (m, 6H), 0.77 (t, *J* = 7.1 Hz, 3H). ^13^C{^1^H} NMR (75 MHz, CDCl_3_): δ 195.3, 143.2, 139.9, 137.1, 134.4, 133.6, 129.9, 129.8, 129.5, 129.3, 129.1, 129.0, 127.3, 65.3, 46.8, 31.0, 30.5, 26.3, 22.3, 21.5, 13.9. HRMS (ESI) *m*/*z*: calcd for C_27_H_30_ClNO_3_SNa [M + Na]^+^, 506.1527; found, 506.1544.

#### Compound **3l**

General procedure A was followed, eluting with hexanes/ethyl acetate (0–40%) to afford 72.7 mg (82% yield) of **3l** as a white solid. ^1^H NMR (500 MHz, chloroform-*d*): δ 7.68 (d, *J* = 8.7 Hz, 2H), 7.56–7.48 (m, 4H), 7.23–7.15 (m, 5H), 7.09 (d, *J* = 8.1 Hz, 2H), 6.22 (d, *J* = 7.4 Hz, 1H), 5.96 (d, *J* = 7.4 Hz, 1H), 2.33 (s, 3H). ^13^C{^1^H} NMR (126 MHz, CDCl_3_): δ 193.6, 143.1, 137.3, 135.2, 132.4, 132.0, 130.3, 129.3, 129.2, 129.1, 128.6, 128.0, 126.9, 61.7, 21.4. HRMS (ESI) *m*/*z*: calcd for C_21_H_18_BrNO_3_SNa [M + Na]^+^, 466.0083; found, 466.0100.

#### Compound **3m**

General procedure A was followed, eluting with hexanes/ethyl acetate (0–40%) to afford 43.2 mg (57% yield) of **3m** as a white solid. ^1^H NMR (500 MHz, chloroform-*d*): δ 7.73 (d, *J* = 7.0 Hz, 2H), 7.54 (d, *J* = 6.9 Hz, 2H), 7.23–7.14 (m, 7H), 7.08 (d, *J* = 7.8 Hz, 2H), 6.26 (d, *J* = 7.3 Hz, 1H), 5.99 (d, *J* = 7.2 Hz, 1H), 2.36 (s, 3H), 2.32 (s, 3H). ^13^C{^1^H} NMR (126 MHz, CDCl_3_): δ 194.0, 145.1, 143.0, 137.4, 136.0, 131.2, 129.4, 129.3, 129.1, 129.0, 128.3, 128.1, 126.9, 61.5, 21.7, 21.4. HRMS (ESI) *m*/*z*: calcd for C_22_H_22_NO_3_S [M + H]^+^, 380.1315; found, 380.1317.

#### Compound **3n**

General procedure A was followed, eluting with hexanes/ethyl acetate (0–40%) to afford 66.6 mg (88% yield) of **3n** as a white solid. ^1^H NMR (300 MHz, chloroform-*d*): δ 7.82 (d, *J* = 8.1 Hz, 2H), 7.60–7.47 (m, 3H), 7.43–7.33 (m, 2H), 7.11–7.06 (m, 4H), 6.99 (d, *J* = 7.9 Hz, 2H), 6.26 (d, *J* = 7.5 Hz, 1H), 5.98 (d, *J* = 7.5 Hz, 1H), 2.32 (s, 3H), 2.25 (s, 3H). ^13^C{^1^H} NMR (75 MHz, CDCl_3_): δ 194.6, 143.0, 138.4, 137.4, 133.82, 133.76, 132.6, 129.7, 129.2, 128.9, 128.6, 128.0, 127.0, 61.4, 21.4, 21.0. HRMS (ESI) *m*/*z*: calcd for C_21_H_21_NO_3_SNa [M + Na]^+^, 402.1134; found, 402.1142.

#### Compound **3o**(30)

General procedure A was followed, eluting with hexanes/ethyl acetate (0–40%) to afford 60.6 mg (76% yield) of **3o** as a white solid. ^1^H NMR (500 MHz, chloroform-*d*): δ 7.81 (d, *J* = 7.0 Hz, 2H), 7.58–7.49 (m, 3H), 7.40 (dd, *J* = 7.8, 7.8 Hz, 2H), 7.17–7.06 (m, 6H), 6.27 (d, *J* = 7.0 Hz, 1H), 5.99 (d, *J* = 7.1 Hz, 1H), 2.35 (s, 3H). ^13^C{^1^H} NMR (126 MHz, CDCl_3_): δ 194.1, 143.3, 137.3, 134.6, 134.2, 134.1, 133.5, 129.5, 129.4, 129.2, 128.9, 128.8, 126.9, 60.9, 21.4. HRMS (ESI) *m*/*z*: calcd for C_21_H_18_ClNO_3_SNa [M + Na]^+^, 422.0588; found, 422.0585.

#### Compound **3p**

General procedure A was followed, eluting with hexanes/ethyl acetate (0–40%) to afford 52.7 mg (69% yield) of **3p** as a white solid. ^1^H NMR (500 MHz, chloroform-*d*): δ 7.80 (d, *J* = 7.7 Hz, 2H), 7.58–7.51 (m, 3H), 7.43–7.35 (m, 2H), 7.21–7.15 (m, 2H), 7.10 (d, *J* = 8.0 Hz, 2H), 6.92–6.83 (m, 2H), 6.24 (d, *J* = 7.0 Hz, 1H), 6.01 (d, *J* = 8.9 Hz, 1H), 2.34 (s, 3H). ^13^C{^1^H} NMR (126 MHz, CDCl_3_): δ 194.4, 162.6 (d, *J* = 248.6 Hz), 143.3, 137.4, 134.1, 133.6, 131.6 (d, *J* = 3.3 Hz), 130.0 (d, *J* = 8.5 Hz), 129.4, 128.9, 128.8, 126.9, 116.1 (d, *J* = 21.9 Hz), 60.9, 21.4. ^19^F NMR (471 MHz, CDCl_3_): δ −112.7. HRMS (ESI) *m*/*z*: calcd for C_21_H_19_FNO_3_S [M + H]^+^, 384.1064; found, 384.1062.

#### Compound **3q**

General procedure A was followed, eluting with hexanes/ethyl acetate (0–40%) to afford 66.9 mg (76% yield) of **3q** as a white solid. ^1^H NMR (500 MHz, chloroform-*d*): δ 7.90 (d, *J* = 7.1 Hz, 2H), 7.60 (d, *J* = 9.0 Hz, 2H), 7.57 (dt, *J* = 5.2, 2.5 Hz, 4H), 7.51–7.44 (m, 2H), 7.41 (t, *J* = 7.3 Hz, 1H), 7.27–7.19 (m, 5H), 7.10 (d, *J* = 7.8 Hz, 2H), 6.26 (d, *J* = 7.3 Hz, 1H), 6.05 (d, *J* = 7.2 Hz, 1H), 2.33 (s, 3H). ^13^C{^1^H} NMR (126 MHz, CDCl_3_): δ 194.0, 146.7, 143.1, 139.4, 137.5, 135.8, 132.4, 129.6, 129.4, 129.1, 129.0, 128.54, 128.51, 128.2, 127.3, 127.2, 127.0, 61.7, 21.4. HRMS (ESI) *m*/*z*: calcd for C_27_H_23_NO_3_SNa [M + Na]^+^, 464.1291; found, 464.1306.

#### Compound **3r**

General procedure A was followed, eluting with hexanes/ethyl acetate (10–40%) to afford 31.7 mg (43% yield) of **3r** as a white solid. ^1^H NMR (500 MHz, chloroform-*d*): δ 7.68–7.60 (m, 2H), 7.55 (d, *J* = 7.5 Hz, 3H), 7.27–7.19 (m, 5H), 7.09 (d, *J* = 7.7 Hz, 2H), 7.07–7.03 (m, 1H), 6.18 (d, *J* = 6.6 Hz, 1H), 5.82 (d, *J* = 6.7 Hz, 1H), 2.33 (d, *J* = 2.6 Hz, 3H). ^13^C{^1^H} NMR (126 MHz, CDCl_3_): δ 187.1, 143.2, 140.3, 137.3, 135.9, 135.2, 133.9, 129.3, 129.1, 128.6, 128.3, 128.1, 127.0, 62.6, 21.4. HRMS (ESI) *m*/*z*: calcd for C_19_H_18_NO_3_S_2_ [M + H]^+^, 372.0723; found, 372.0726.

#### Compound **3s**

General procedure A was followed, eluting with hexanes/ethyl acetate (0–40%) to afford 49.0 mg (62% yield) of **3s** as a white solid. ^1^H NMR (300 MHz, chloroform-*d*): δ 7.88–7.74 (m, 2H), 7.59–7.46 (m, 2H), 7.27–7.14 (m, 5H), 7.08 (d, *J* = 7.9 Hz, 2H), 6.99–6.75 (m, 2H), 6.27 (d, *J* = 7.2 Hz, 1H), 5.95 (d, *J* = 7.7 Hz, 1H), 3.83 (s, 2H), 2.32 (s, 3H). ^13^C{^1^H} NMR (126 MHz, CDCl_3_): δ 192.7, 164.1, 143.0, 137.5, 136.2, 131.4, 129.3, 129.0, 128.3, 128.0, 126.9, 126.6, 113.9, 61.3, 55.5, 21.4. HRMS (ESI) *m*/*z*: calcd for C_22_H_21_NO_4_SNa [M + Na]^+^, 418.1083; found, 418.1093.

#### Compound **3t**

General procedure A was followed, eluting with hexanes/ethyl acetate (0–30%) to afford 47.5 mg (69% yield) of **3t** as a white solid. ^1^H NMR (500 MHz, chloroform-*d*): δ 7.54 (d, *J* = 8.3 Hz, 2H), 7.26–7.18 (m, 3H), 7.18–7.07 (m, 4H), 6.04 (d, *J* = 7.8 Hz, 1H), 5.42 (d, *J* = 7.8 Hz, 1H), 2.36 (s, 3H), 0.91 (s, 9H). ^13^C{^1^H} NMR (126 MHz, CDCl_3_): δ 210.5, 143.1, 137.3, 135.2, 129.3, 129.0, 128.5, 128.3, 127.0, 61.0, 43.9, 26.9, 21.4. HRMS (ESI) *m*/*z*: calcd for C_19_H_23_NO_3_SNa [M + Na]^+^, 368.1291; found, 368.1288.

#### Compound **3u**

General procedure A was followed, eluting with hexanes/ethyl acetate (0–30%) to afford 13.3 mg (20% yield) of **3a** as a white solid. ^1^H NMR (500 MHz, chloroform-*d*): δ 7.51 (d, *J* = 7.8 Hz, 2H), 7.28–7.18 (m, 3H), 7.19–7.07 (m, 4H), 6.12 (d, *J* = 5.8 Hz, 1H), 5.17 (d, *J* = 5.7 Hz, 1H), 2.57 (sept, *J* = 7.0 Hz, 1H), 2.36 (s, 3H), 0.95 (d, *J* = 7.0 Hz, 3H), 0.79 (d, *J* = 6.6 Hz, 3H). ^13^C{^1^H} NMR (126 MHz, CDCl_3_): δ 208.2, 143.1, 137.3, 134.9, 129.2, 129.0, 128.6, 128.2, 127.0, 64.2, 37.3, 21.4, 19.0, 17.9. HRMS (ESI) *m*/*z*: calcd for C_18_H_22_NO_3_S [M + H]^+^, 332.1315; found, 332.1314.

## Data Availability

The data underlying this study are available in the published article and its Supporting Information.

## Supporting Information Available

Experimental procedures and spectroscopic data (PDF)
