# Supplementary material for: Iron-Catalyzed Oxidative α-Amination of Ketones with Primary and Secondary Sulfonamides
Source: J Org Chem. 2023 Feb 22;88(5):3353–8. doi: 10.1021/acs.joc.3c00210 (PMC9990065; doi:10.1021/acs.joc.3c00210)
Supplement: Supplementary file 1 — jo3c00210_si_001.pdf [file jo3c00210_si_001.pdf]

Iron-Catalyzed Oxidative α-Amination of Ketones with Primary and Secondary Sulfonamides

Fubin Song, So Hyun Park, Christine Wu, and Alexandra E. Strom*

Smith College
Department of Chemistry
100 Green St
Northampton, MA 01106, United States

*Email: astrom@smith.edu

Table of Contents

1. Amination Reactions 2
1.1 General Procedure for Table S1 .... *2*
1.2 Table S.1. Optimization of reaction conditions *3*
2. Spectroscopic Characterization 3

1. Amination Reactions

1.1 General Procedure for Table S1

To an oven-dried vial in the glovebox was added ketone (0.200 mmol, 1.00 equiv), DDQ (54.5 mg, 0.240 mmol, 1.20 equiv), *p*-toluenesulfonamide (103 mg, 0.600 mmol, 3.00 equiv), iron(III) bromide (11.8 mg, 0.0400 mmol, 0.200 equiv) and an oven-dried stir bar. 1,2-Dichloroethane (1.0 mL, 0.20 M, anhydrous) was added, and the reaction was sealed with a PTFE-lined cap, removed from the glovebox, and heated at the listed temperature in an aluminum heating block for the indicated time. The reaction was allowed to cool to room temperature, then opened to air and 1 mL sat $NH_4Cl_{(aq)}$ was added. The aqueous solution was extracted with DCM until the organic phase was clear, and the combined organic layers were filtered through a pad of silica, washing with 20% MeOH in DCM (10 mL). Ethylene carbonate (8.8 mg, 0.10 mmol, 0.5 equiv) was added and the solvent was removed *in vacuo*. The crude solid was dissolved in $CDCl_3$ (0.5 mL) and a portion of the $CDCl_3$ solution was diluted further with $CDCl_3$ for ^1H NMR analysis.

1.2 Table S.1. Optimization of reaction conditions

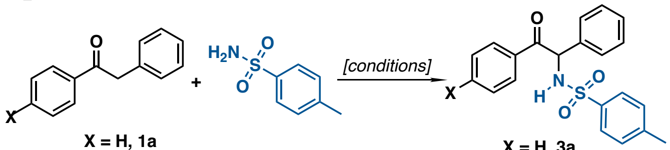

X = H, **1a**
X = F, **1b**
2a
X = H, **3a**
X = F, **3b**

entry	equiv **ketone**	equiv **2a**	catalyst (mol %)	oxidant	solvent	temp (°C)	additive (equiv)	time (h)	yield[b]
1	1.0 **1b**	1.2	$FeBr_3$ (20)	DDQ	1,2 DCE	100	(none)	24	47
2	1.0 **1b**	1.2	$FeBr_3$ (20)	DDQ	1,2 DCE	110	(none)	4	52
3	1.0 **1b**	1.0	$FeCl_3$ (20)	DDQ	1,2 DCE	100	(none)	24	43
4	1.0 **1a**	1.0	$FeCl_3$ (20)	DDQ	1,2 DCE	100	DMAP (0.2)	24	0
5	1.0 **1a**	1.0	$FeCl_3$ (20)	DDQ	1,2 DCE	100	4-cyanopyridine (0.2)	24	6
6	1.0 **1a**	1.0	$FeCl_3$ (20)	DDQ	1,2 DCE	100	2,6-lutidine (0.2)	24	0
7	1.0 **1a**	1.0	$FeCl_3$ (20)	DDQ	1,2 DCE	100	phen (0.2)	24	0
8	1.0 **1a**	1.0	$FeCl_3$ (20)	DDQ	1,2 DCE	100	bipy (0.2)	24	0
9	1.0 **1a**	1.0	$FeCl_3$ (20)	DDQ	DME	100	(none)	24	0
10	1.0 **1a**	1.0	$FeCl_3$ (20)	DDQ	HFIP	100	(none)	24	0
11	1.0 **1a**	1.0	$FeCl_3$ (20)	DDQ	1,2 DCE	100	$NaBF_4$ (1.0)	24	18
12	1.0 **1a**	3.0	$FeBr_3$ (20)	DDQ	1,2 DCE	100	tiron (0.2)	4	45
13	1.0 **1b**	3.0	$FeBr_3$ (20)	DDQ	1,2 DCE	100	(none)	4	77
14	1.0 **1b**	3.0	FeF_3 (20)	DDQ	1,2 DCE	100	(none)	4	0
15	1.0 **1b**	3.0	$CuCl_2$ (20)	DDQ	1,2 DCE	100	(none)	4	0
16	1.0 **1b**	3.0	$FeBr_3$ (20)	DDQ	1,2 DCE	80	(none)	4	60
17	1.0 **1b**	3.0	$MnCl_2$ (20)	DDQ	1,2 DCE	100	(none)	4	0
18	1.0 **1a**	1.2	$FeBr_3$ (20)	PIDA	1,2 DCE	100	(none)	24	5
19	1.0 **1a**	3.0	$FeBr_3$ (20)	DMP	1,2 DCE	100	(none)	24	0
20	1.0 **1b**	3.0	$FeBr_3$ (20)	*o*-chloranil	1,2 DCE	100	(none)	4	23
21	1.0 **1b**	3.0	-	DDQ	1,2 DCE	100	(none)	4	0
22	1.0 **1b**	3.0	$FeBr_3$ (20)	-	1,2 DCE	100	(none)	4	0
23	1.0 **1b**	3.0	$FeBr_3$ (200)	DDQ (0.2)	1,2 DCE	100	(none)	4	18
24	1.0 **1a**	1.2	$FeBr_3$ (100)	-	1,2 DCE	100	(none)	22	0
25	1.0 **1b**	3.0	$FeBr_3$ (20)	DDQ	1,4-dioxane	100	(none)	4	0
26	1.0 **1b**	3.0	$FeBr_3$ (20)	DDQ	1,2 DCE	100	$AgPF_6$ (0.2)	4	64
27	1.0 **1b**	3.0	$FeBr_3$ (20)	DDQ	1,2 DCE	100	$CuCl_2$ (0.20)	4	78
28	1.0 **1b**	3.0	$FeBr_3$ (20)	DDQ	1,2 DCE	100	$AgBF_4$ (0.2)	4	72
29	1.0 **1b**	3.0	$FeBr_3$ (20)	DDQ	1,2 DCE	100	Cu_2O (0.20)	4	76
30	1.0 **1b**	3.0	$FeBr_3$ (20)	DDQ	1,2 DCE	100	bipy (0.2)	4	0
31	1.0 **1b**	3.0	$FeCl_2{\cdot}4H_2O$ (20)	DDQ	1,2 DCE	100	(none)	4	54
32	1.0 **1b**	3.0	$Fe(OAc)_2$ (20)	DDQ	1,2 DCE	100	(none)	4	0
33	1.0 **1b**	3.0	$FeCl_2$ (20)	DDQ	1,2 DCE	100	(none)	4	8
34	1.0 **1b**	3.0	$Fe(OTf)_2$ (20)	DDQ	1,2 DCE	100	(none)	4	2
35	1.0 **1b**	3.0	$Fe(acac)_3$ (20)	DDQ	1,2 DCE	100	(none)	4	0
36	1.0 **1b**	1.2	$FeBr_3$ (20)	DDQ	1,2 DCE	100	(R,R)-phen pybox (0.2)	4	0

2. Spectroscopic Characterization

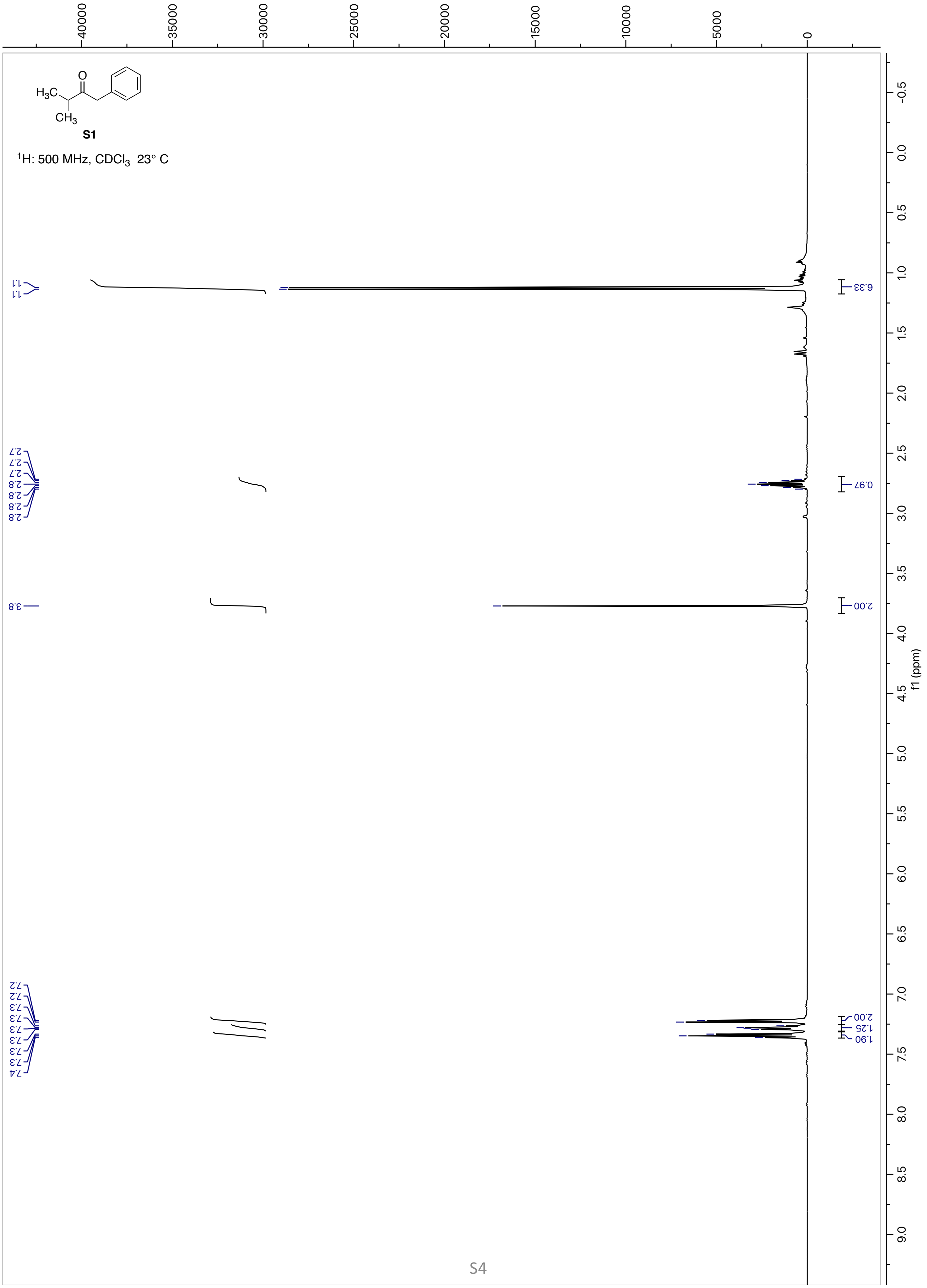

S1
^{1}H: 500 MHz, CDCl$_3$ 23° C
f1 (ppm)

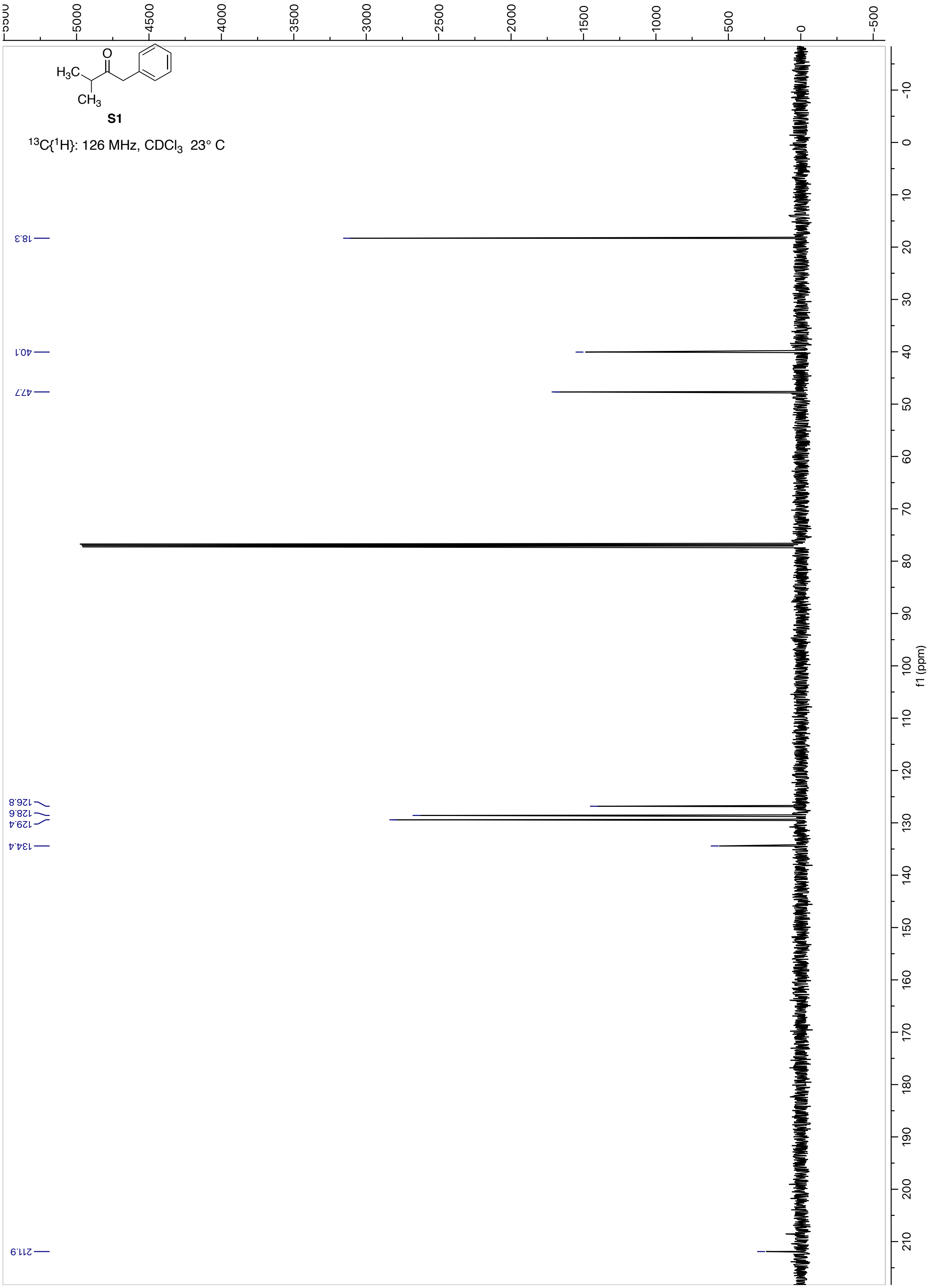
S1
$^{13}C\{^1H\}$: 126 MHz, $CDCl_3$ 23° C
18.3
40.1
47.7
126.8
128.6
129.4
134.4
211.9
f1 (ppm)

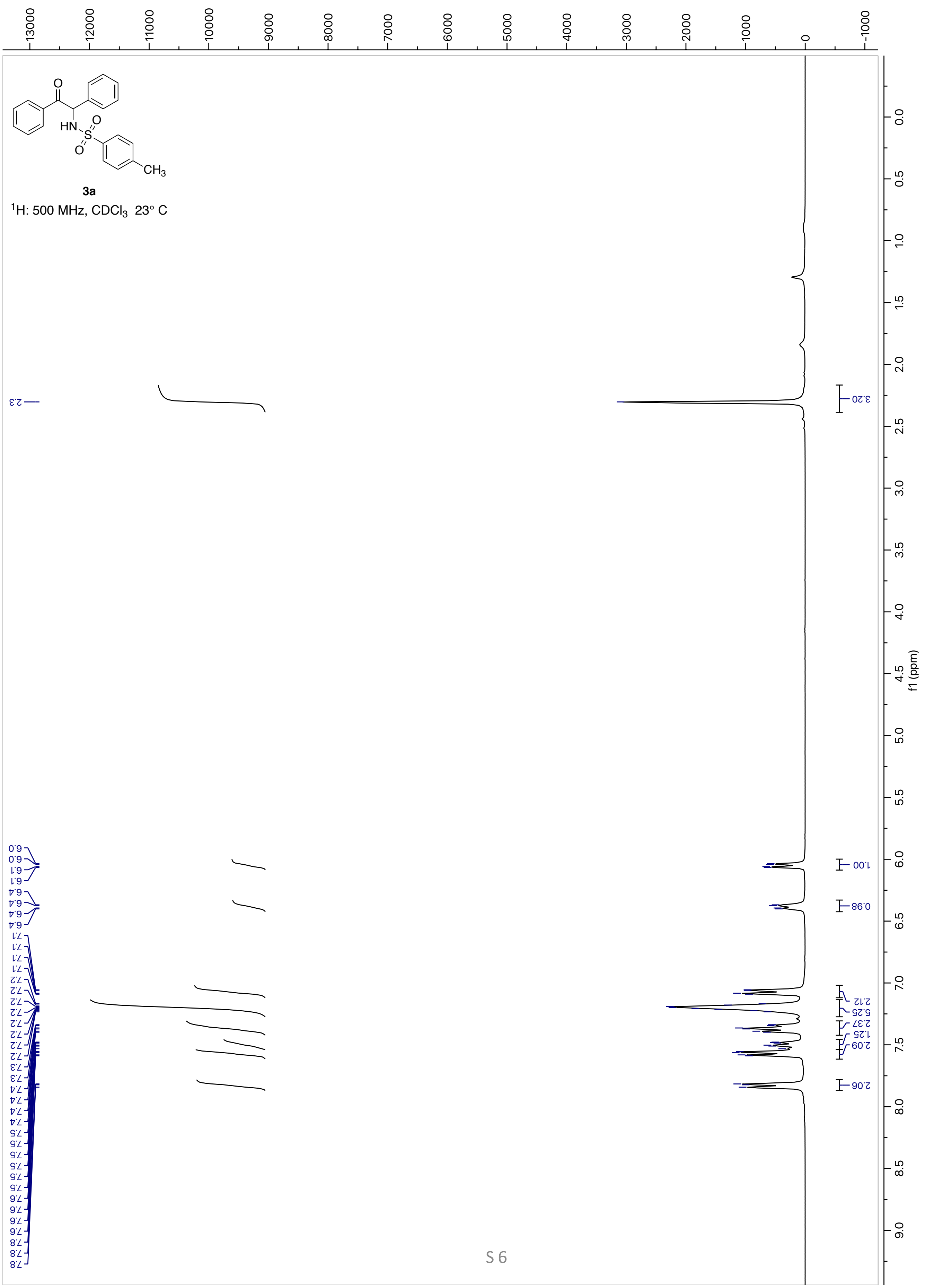
3a
1H: 500 MHz, CDCl3 23° C
f1 (ppm)

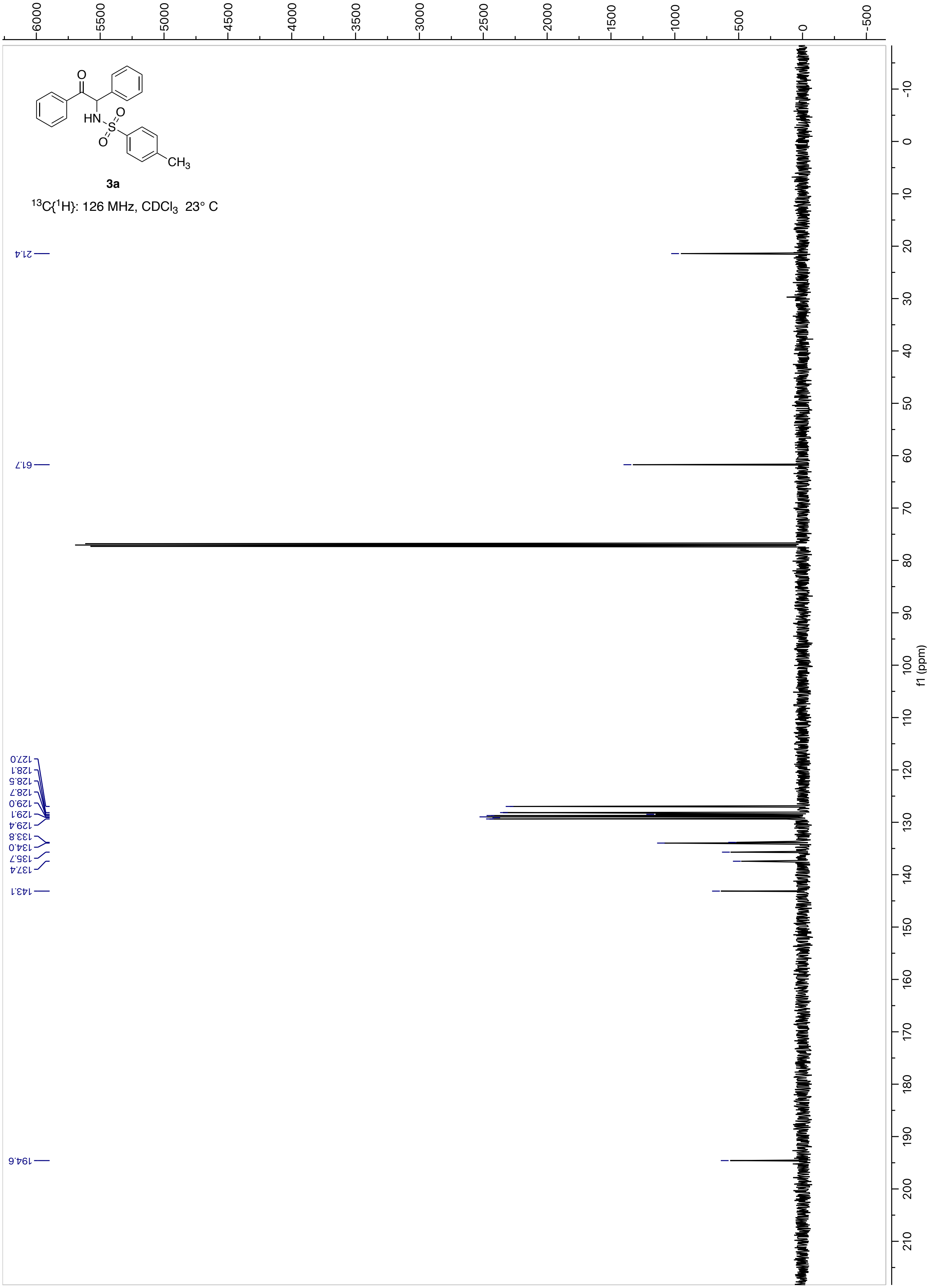

3a
$^{13}C\{^{1}H\}$: 126 MHz, $CDCl_3$ 23° C
21.4
61.7
127.0
128.1
128.5
128.7
129.0
129.1
129.4
133.8
134.0
135.7
137.4
143.1
194.6
f1 (ppm)

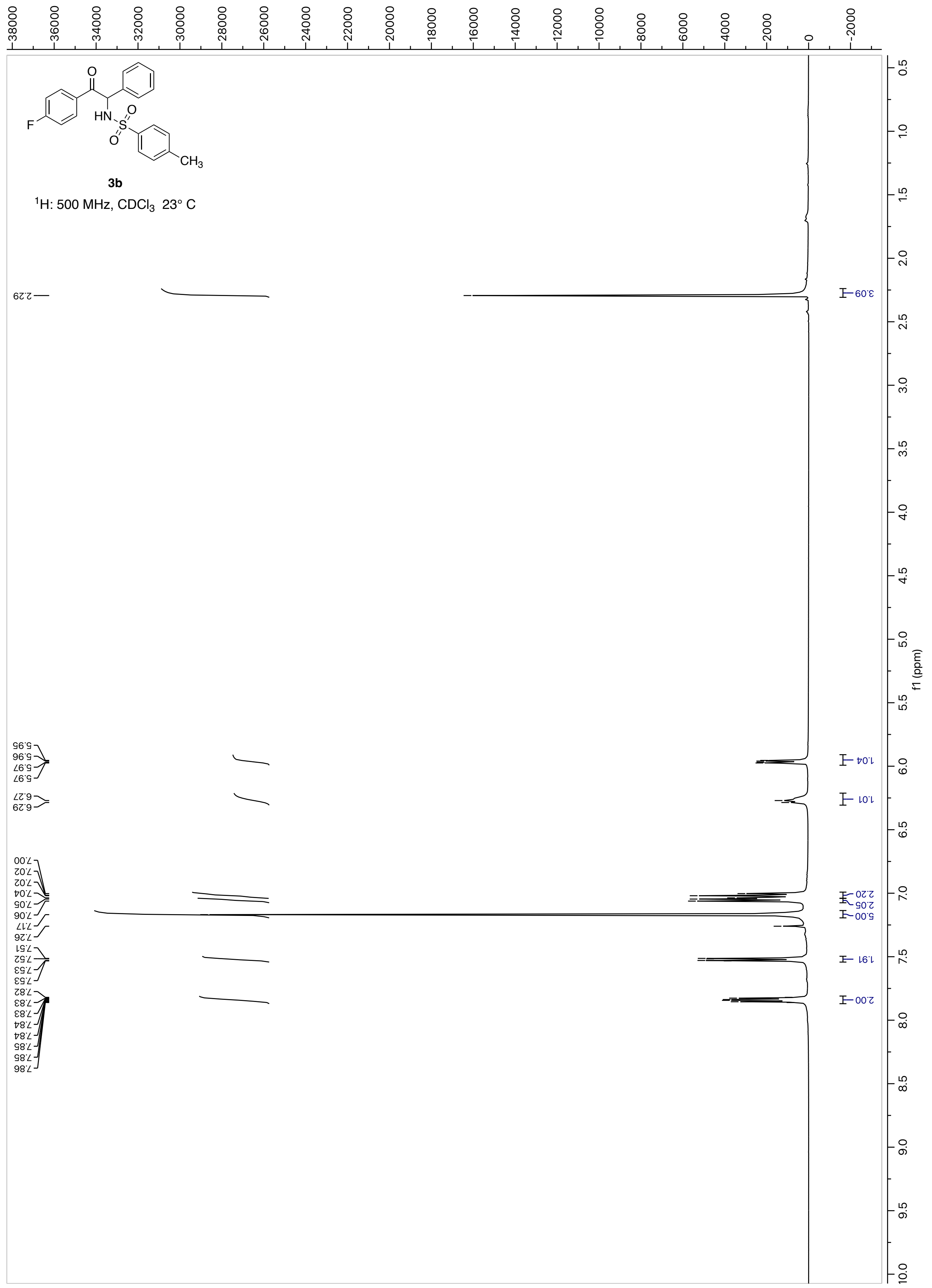
3b
¹H: 500 MHz, CDCl₃ 23° C
f1 (ppm)

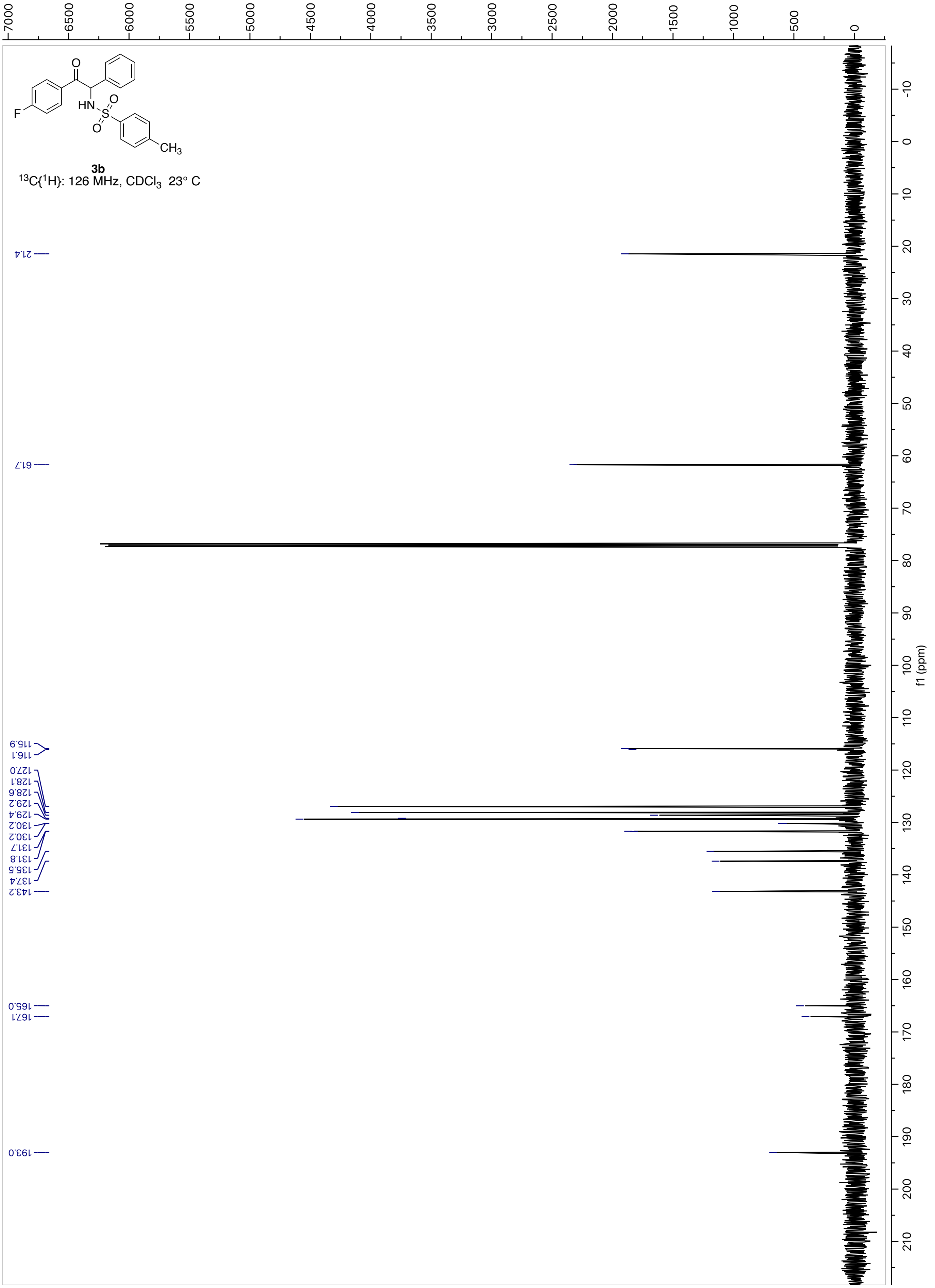
3b
$^{13}C\{^1H\}$: 126 MHz, $CDCl_3$ 23° C
21.4
61.7
115.9
116.1
127.0
128.1
128.6
129.2
129.4
130.2
130.2
131.7
131.8
135.5
137.4
143.2
165.0
167.1
193.0
f1 (ppm)

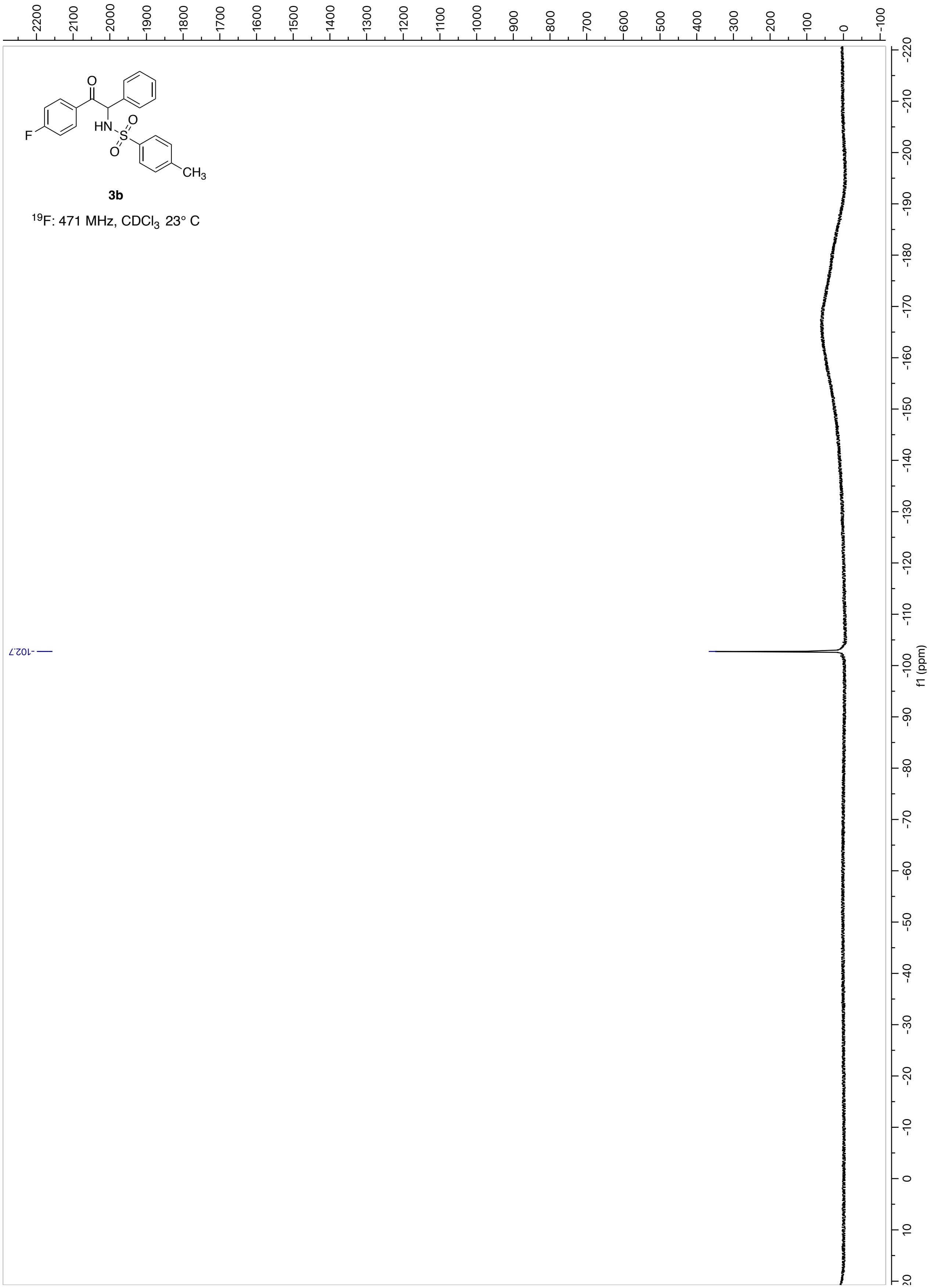
3b
^{19}F: 471 MHz, $CDCl_3$ 23° C
-102.7
f1 (ppm)

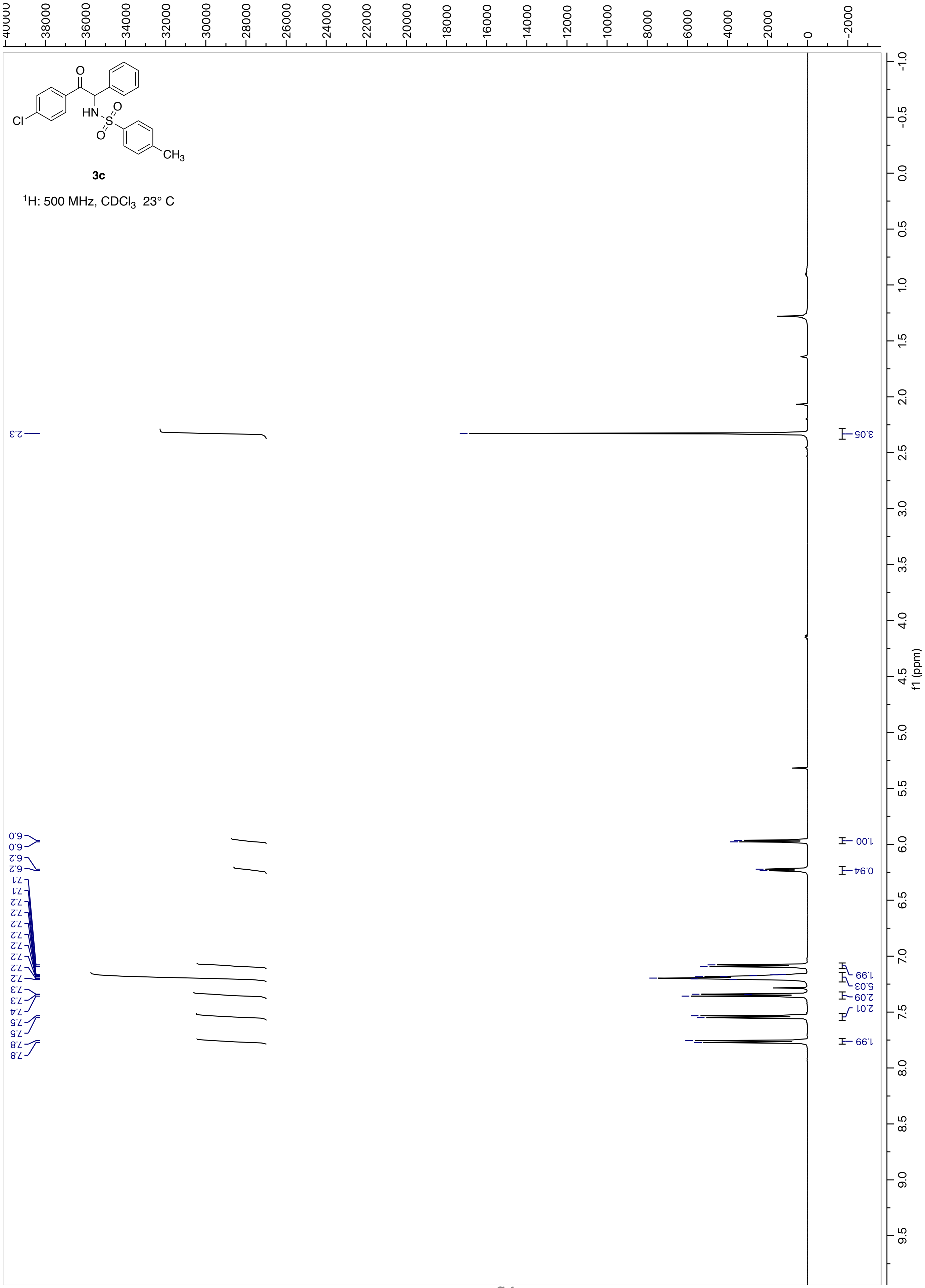
3c
1H: 500 MHz, CDCl3 23° C
f1 (ppm)

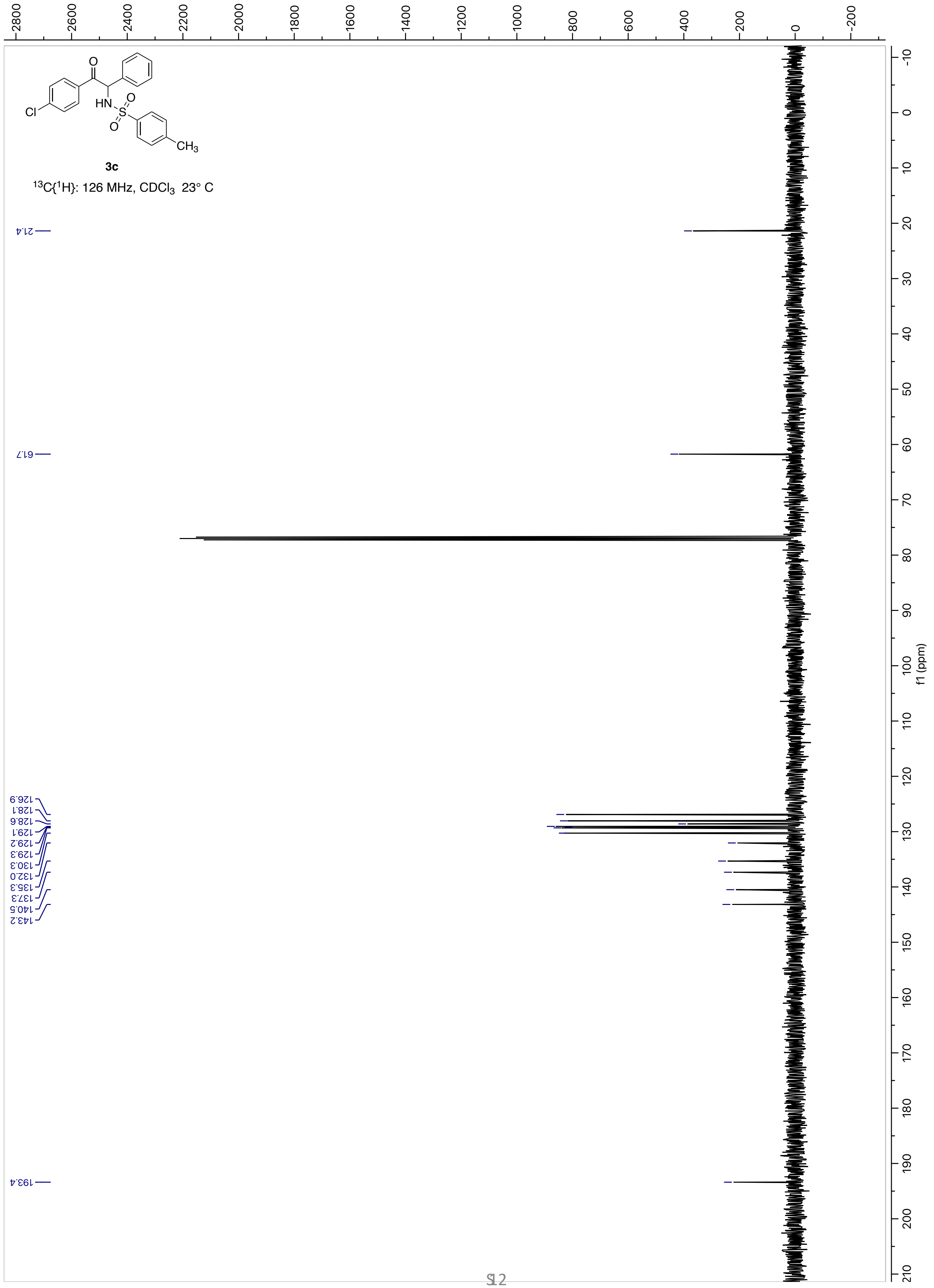
3c
$^{13}C\{^1H\}$: 126 MHz, $CDCl_3$ 23° C
21.4
61.7
126.9
128.1
128.6
129.1
129.2
129.3
130.3
132.0
135.3
137.3
140.5
143.2
193.4
f1 (ppm)

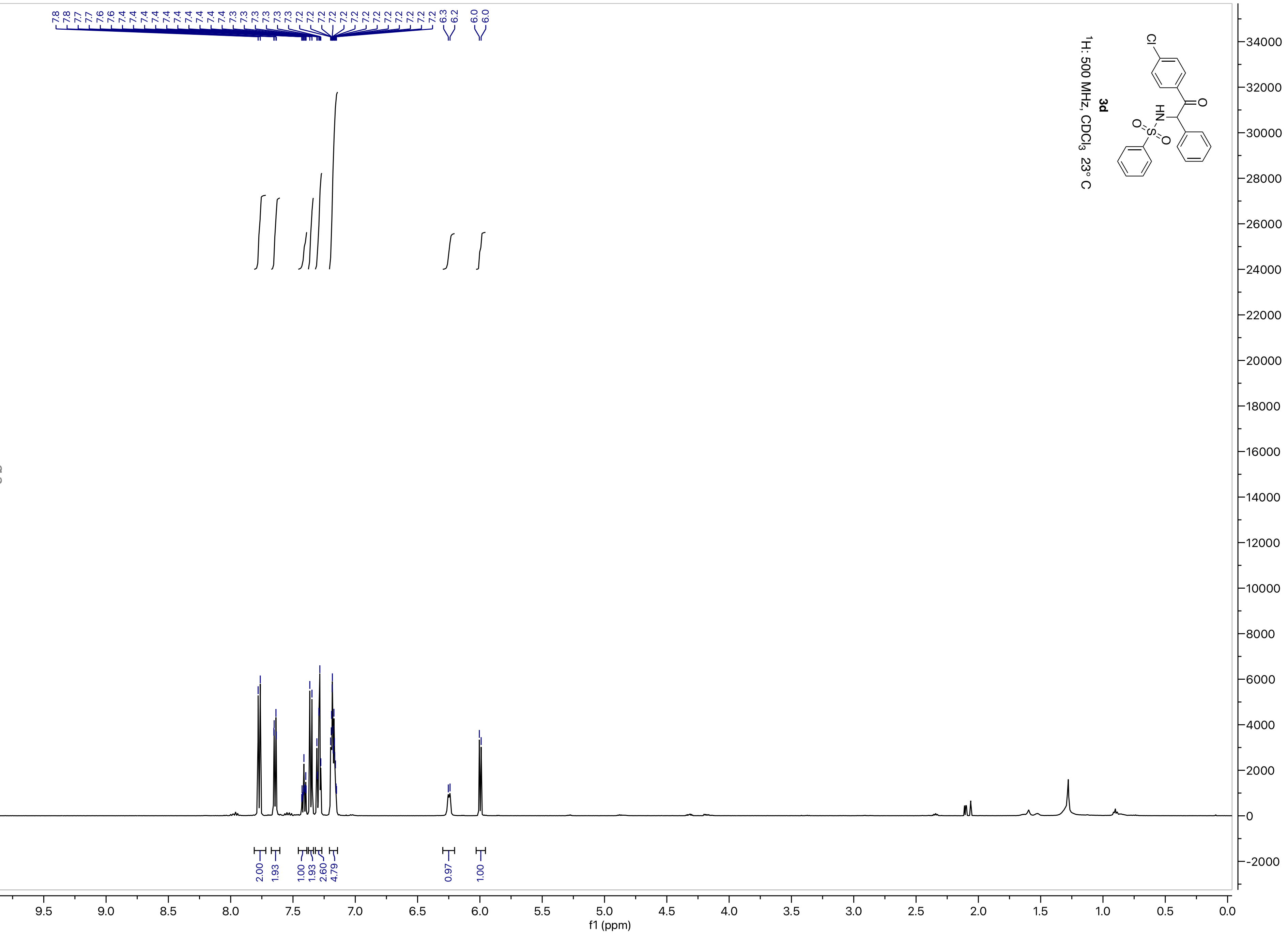
Cl
O
HN
S
O
O
3d
1H: 500 MHz, CDCl3 23° C
7.8
7.8
7.7
7.7
7.6
7.6
7.4
7.4
7.4
7.4
7.4
7.4
7.4
7.4
7.4
7.4
7.3
7.3
7.3
7.3
7.3
7.3
7.2
7.2
7.2
7.2
7.2
7.2
7.2
7.2
7.2
7.2
7.2
7.2
7.2
6.3
6.2
6.0
6.0
2.00
1.93
1.00
1.93
2.60
4.79
0.97
1.00
f1 (ppm)

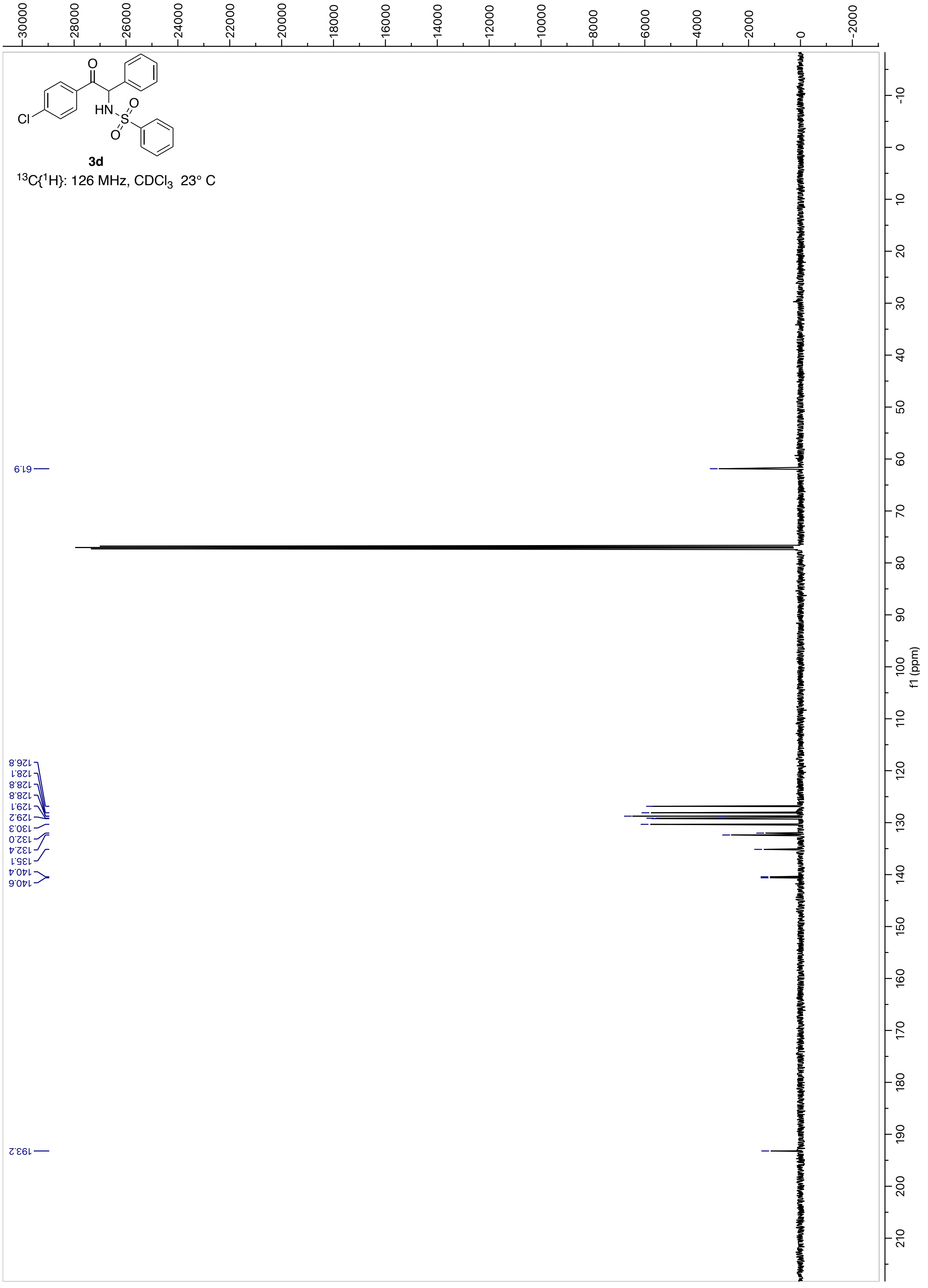
3d
$^{13}C\{^{1}H\}$: 126 MHz, $CDCl_3$ 23° C
61.9
126.8
128.1
128.8
128.8
129.1
129.2
130.3
132.0
132.4
135.1
140.4
140.6
193.2
f1 (ppm)

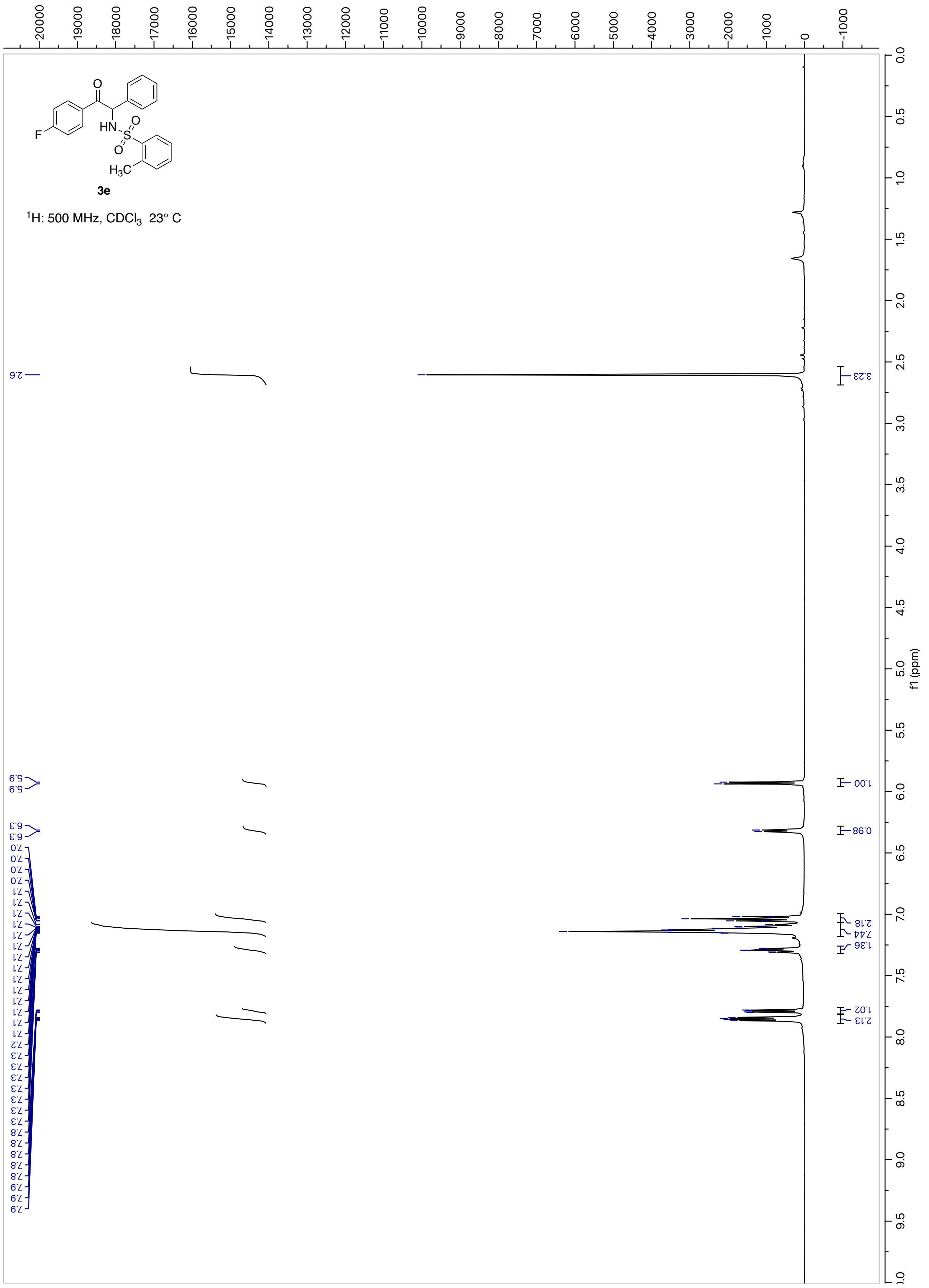

3e
1H: 500 MHz, CDCl3 23° C
f1 (ppm)
3.23
1.00
0.98
2.18
7.44
1.36
1.02
2.13

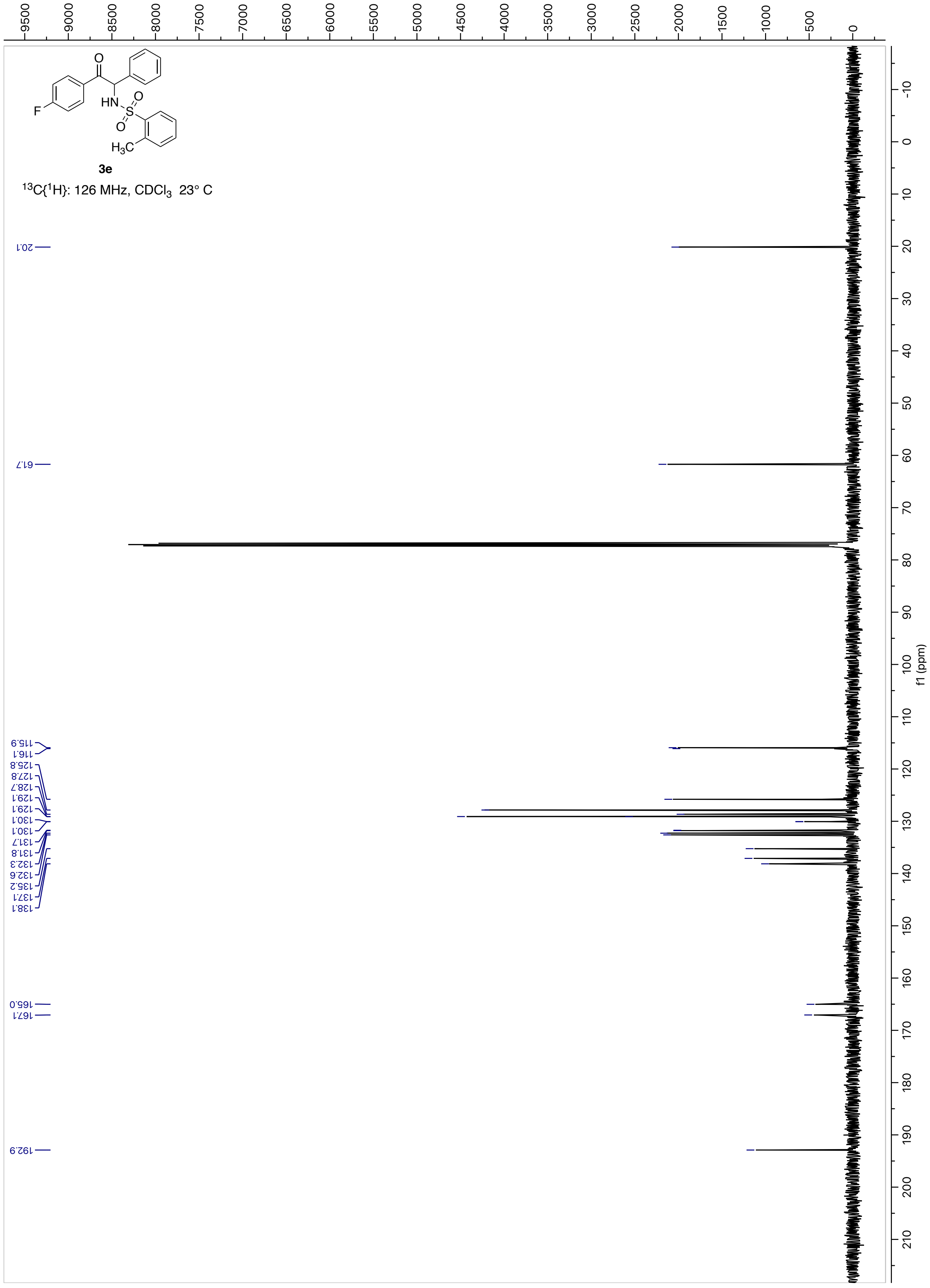

3e
$^{13}C\{^{1}H\}$: 126 MHz, $CDCl_3$ 23° C
f1 (ppm)

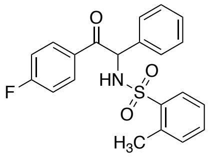

3e

^{19}F: 471 MHz, $CDCl_3$ 23° C

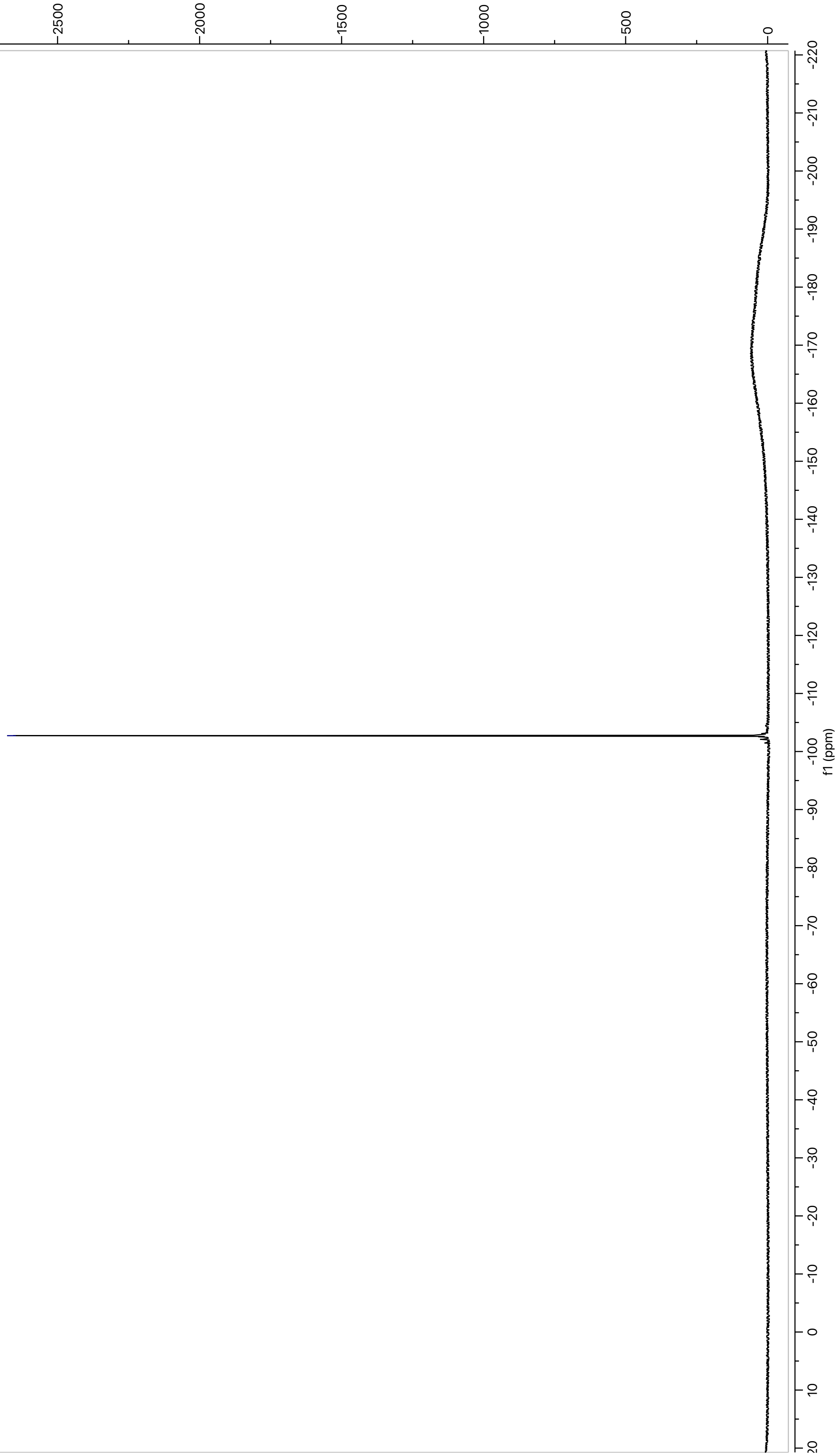

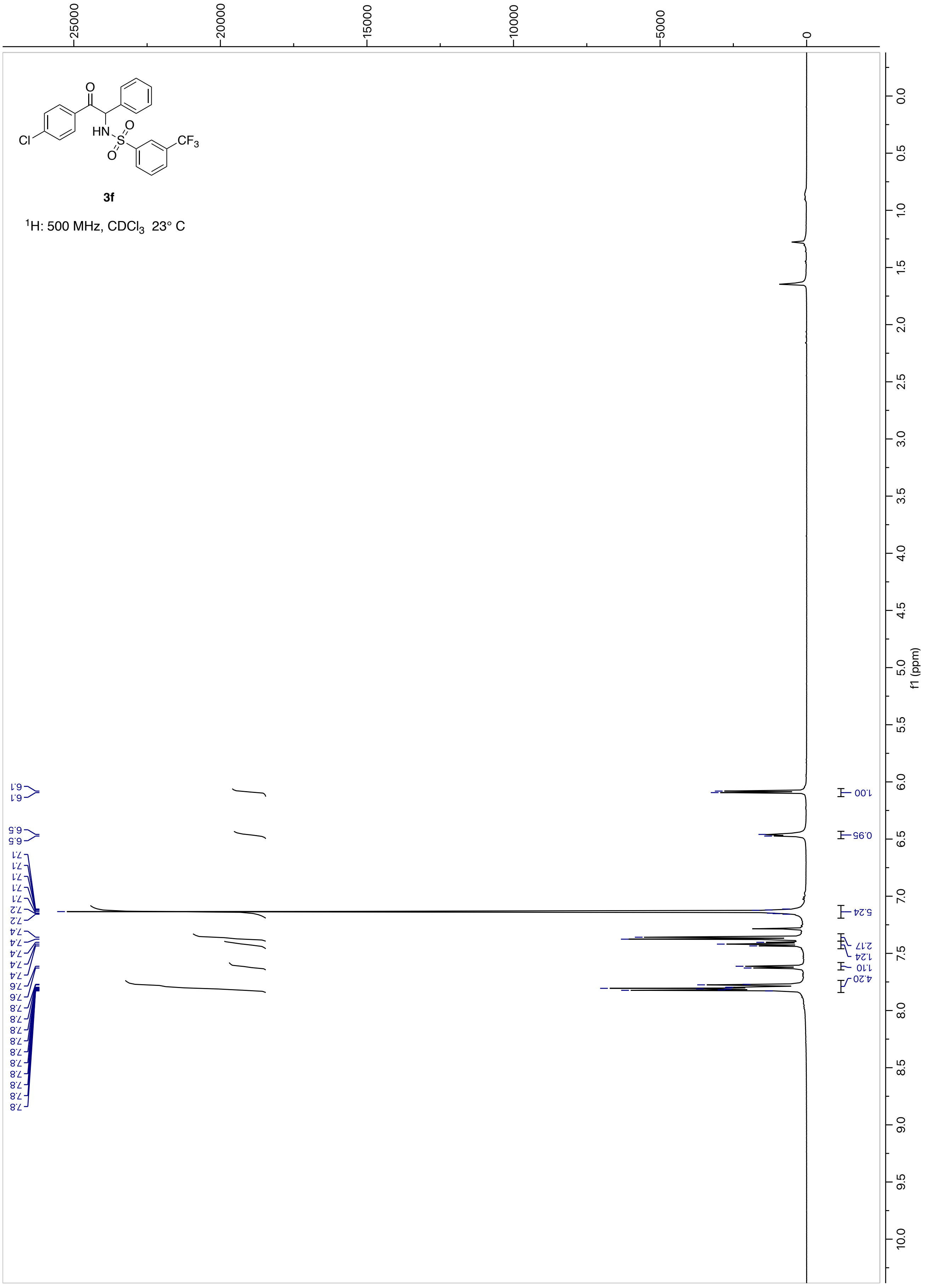
3f
1H: 500 MHz, CDCl3 23° C
f1 (ppm)

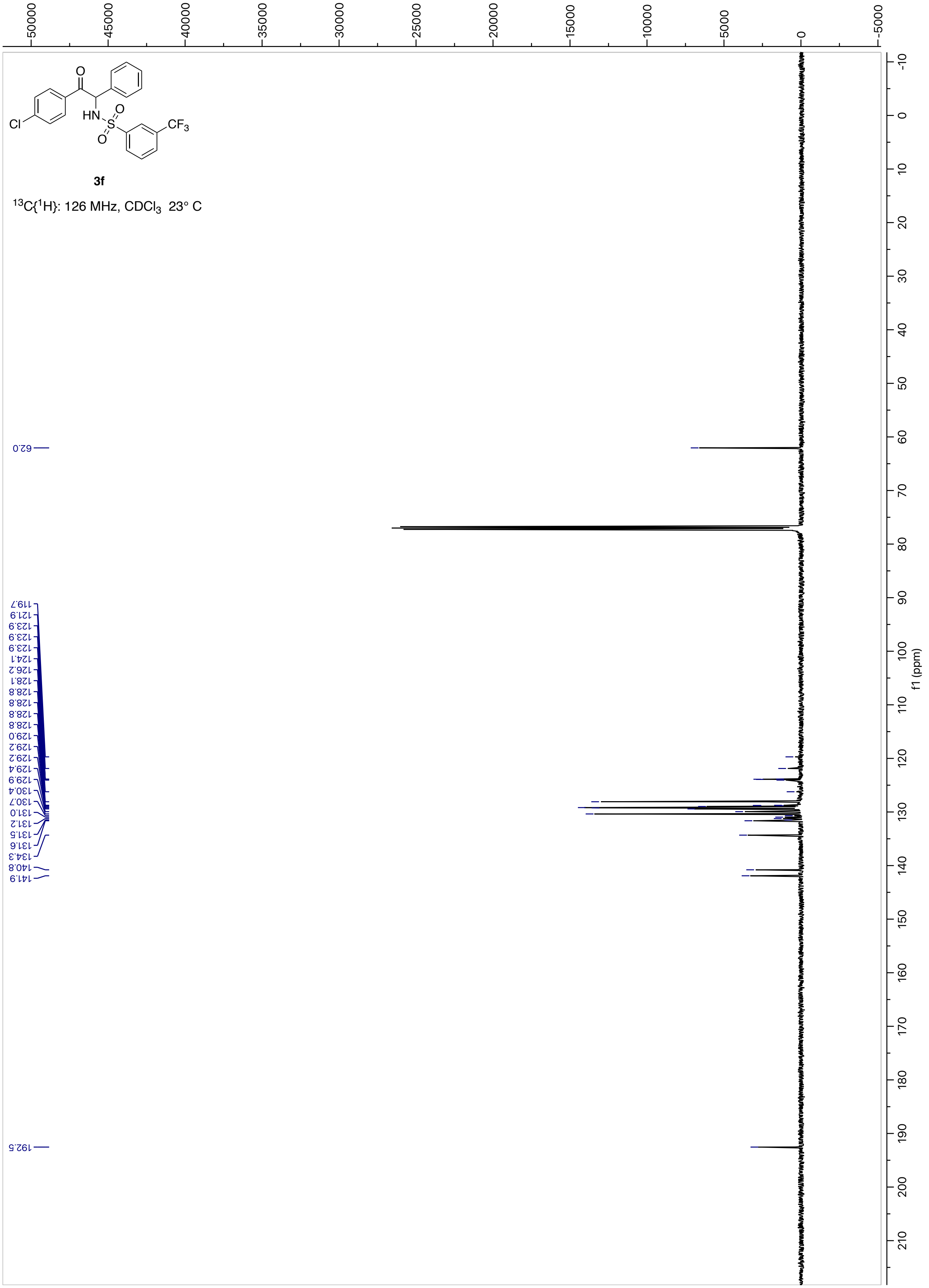

O
HN
S
O
O
Cl
CF3
3f
13C{1H}: 126 MHz, CDCl3 23° C
62.0
119.7
121.9
123.9
123.9
123.9
124.1
126.2
128.1
128.8
128.8
128.8
128.8
129.0
129.2
129.2
129.4
129.9
130.4
130.7
131.0
131.2
131.5
131.6
134.3
140.8
141.9
192.5
f1 (ppm)

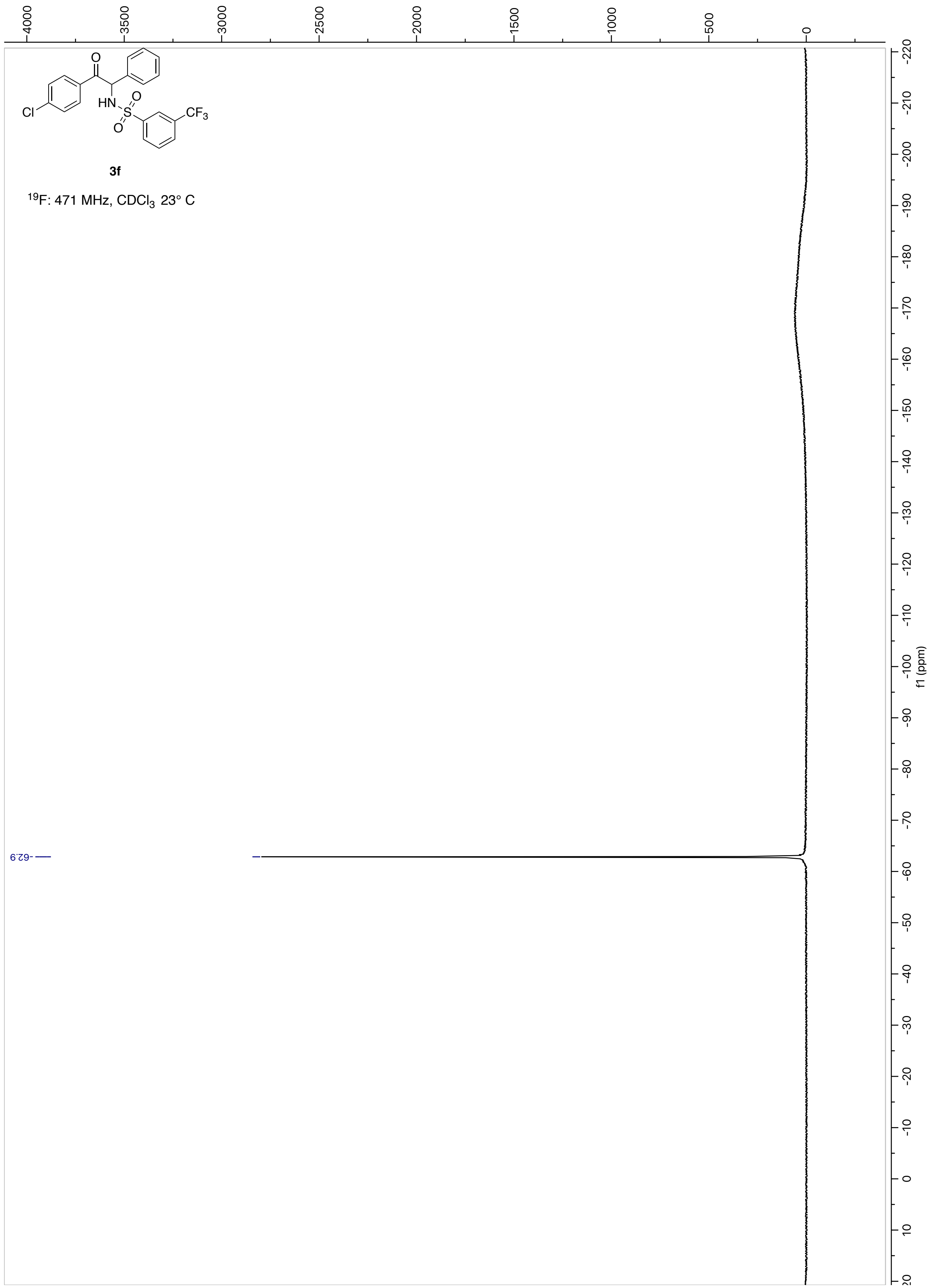
3f
^{19}F: 471 MHz, CDCl$_3$ 23° C
-62.9
f1 (ppm)

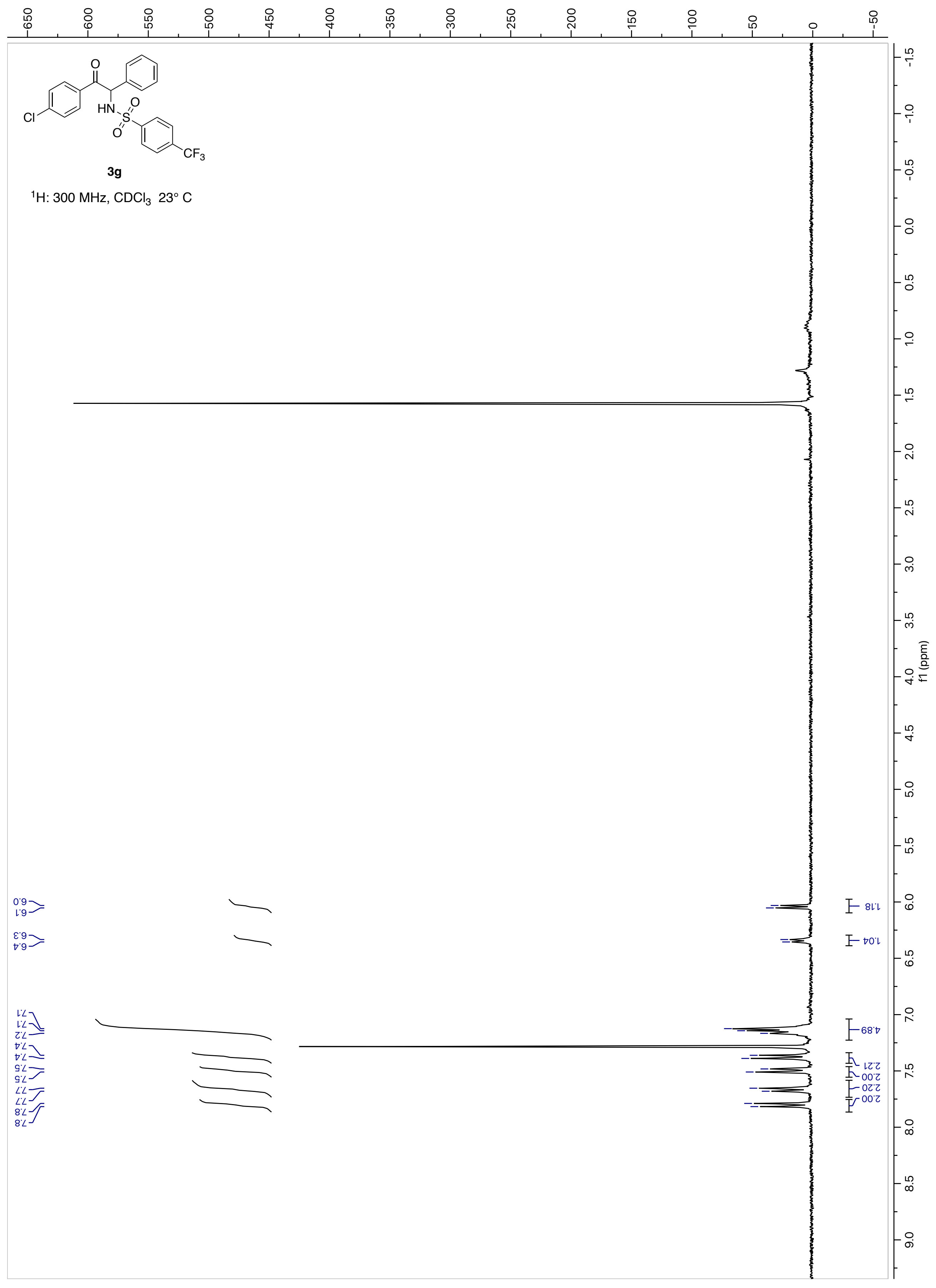

Cl
O
HN
S
O
O
CF3
3g
1H: 300 MHz, CDCl3 23° C
f1 (ppm)
1.18
1.04
4.89
2.21
2.00
2.20
2.00

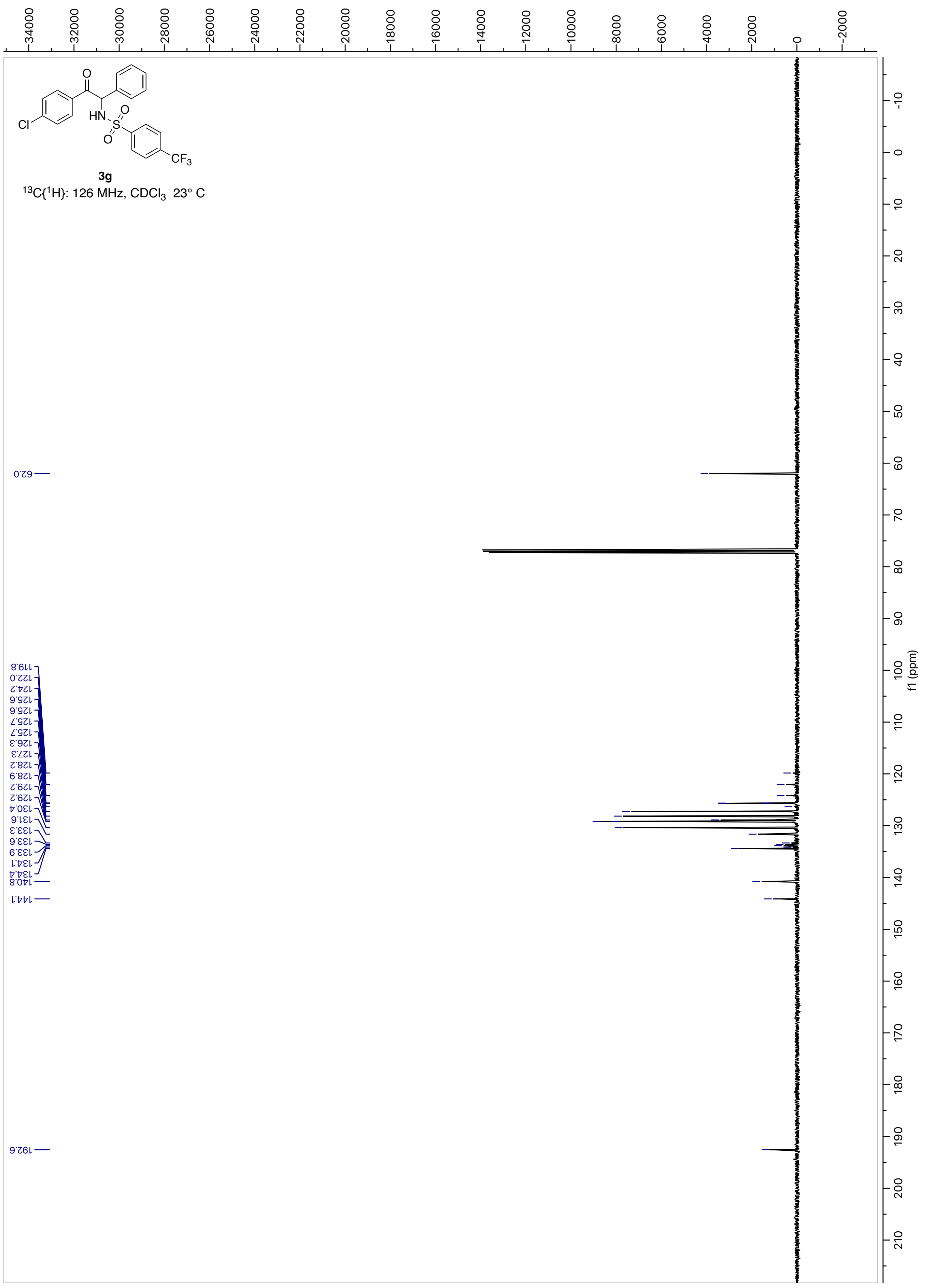
Cl
O
HN
S
O
O
CF3
3g
13C{1H}: 126 MHz, CDCl3 23° C
62.0
119.8
122.0
124.2
125.6
125.6
125.7
125.7
126.3
127.3
128.2
128.9
129.2
129.2
130.4
131.6
133.3
133.6
133.9
134.1
134.4
140.8
144.1
192.6
f1 (ppm)

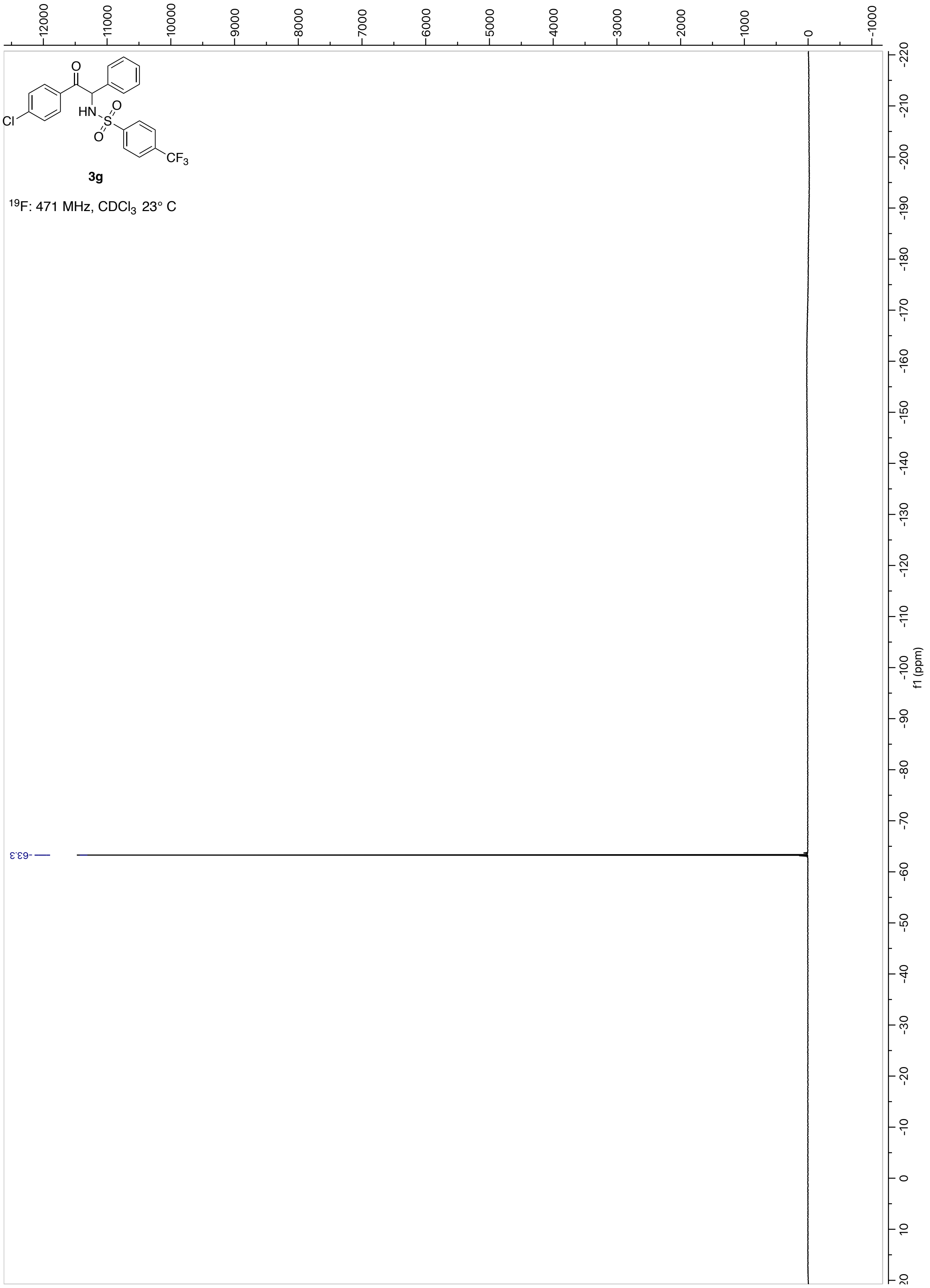
3g
19F: 471 MHz, CDCl3 23° C
-63.3
f1 (ppm)

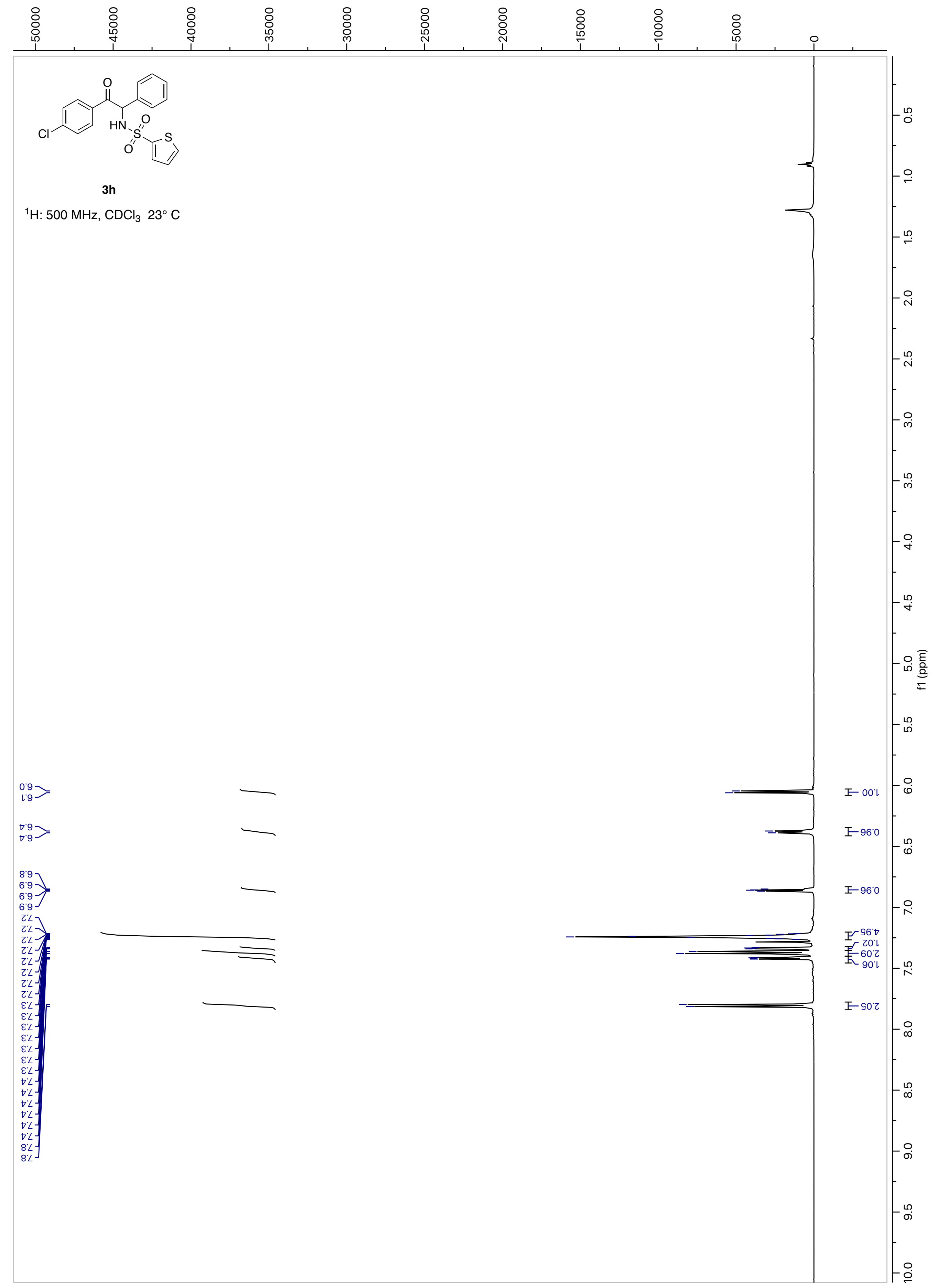
3h
^{1}H: 500 MHz, CDCl$_3$ 23° C
f1 (ppm)

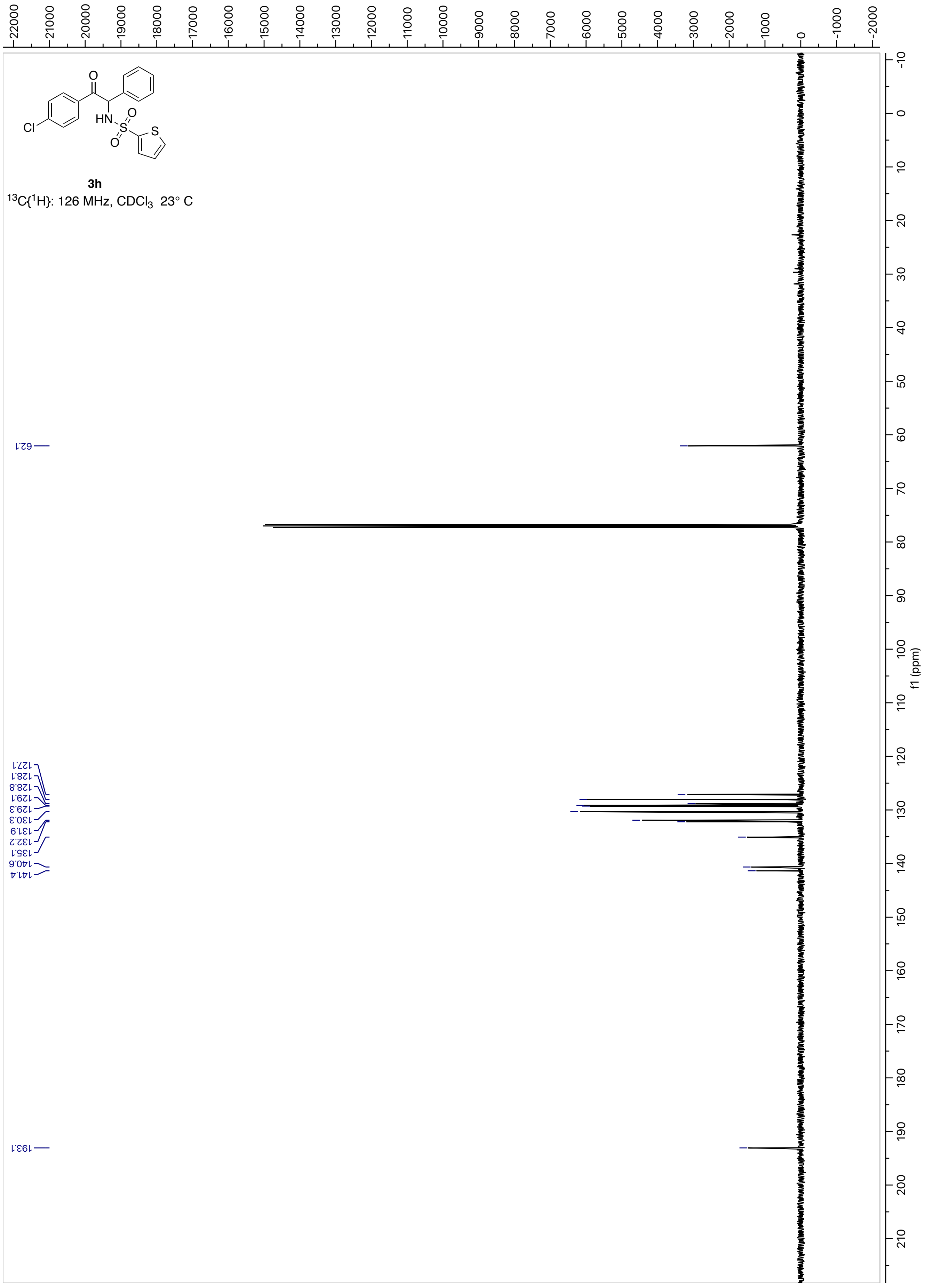
3h
$^{13}C\{^{1}H\}$: 126 MHz, $CDCl_3$ 23° C
193.1
141.4
140.6
135.1
132.2
131.9
130.3
129.3
129.1
128.8
128.1
127.1
62.1
f1 (ppm)

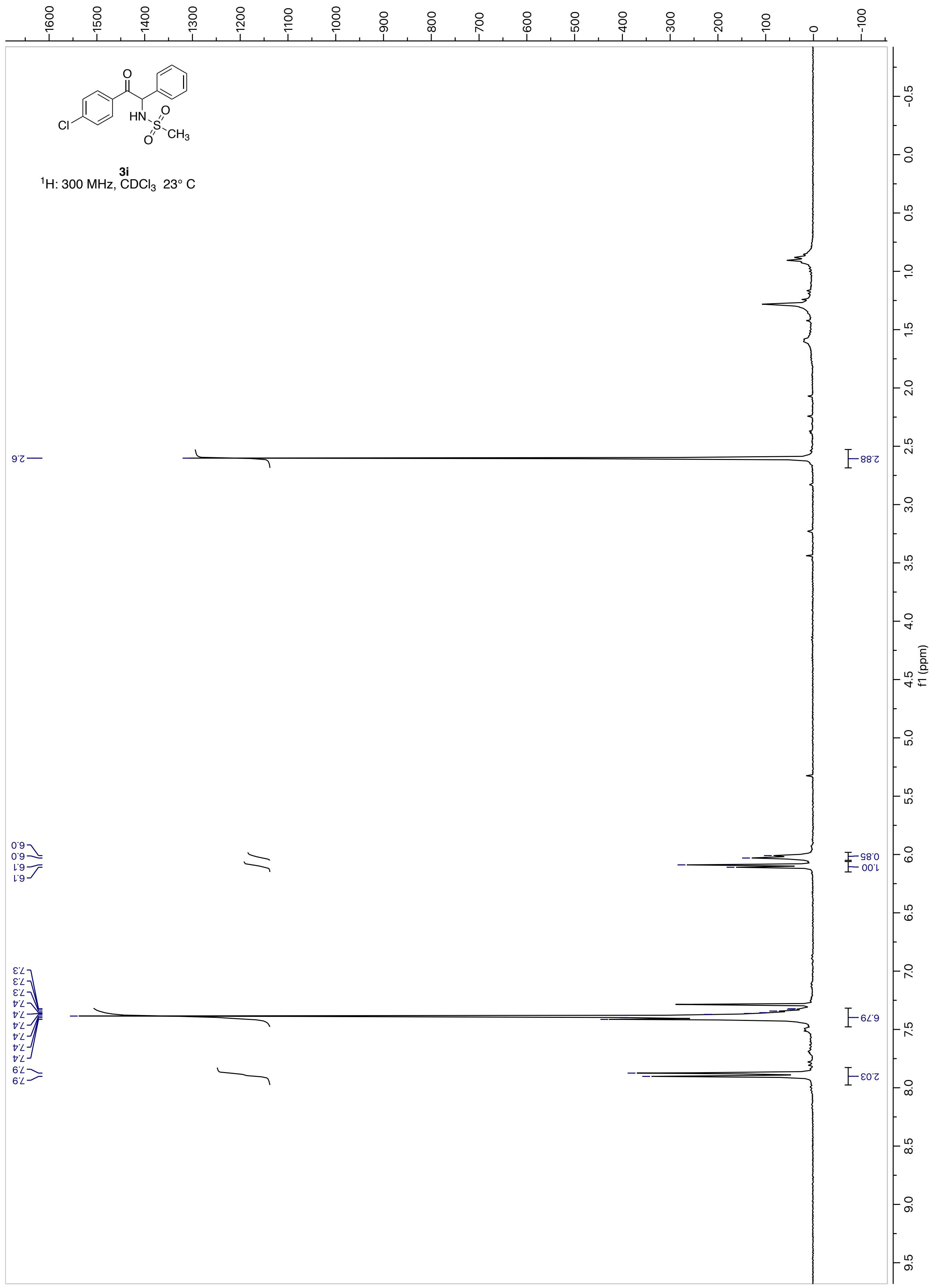
3i
^{1}H: 300 MHz, $CDCl_3$ 23° C
f1 (ppm)
2.88
0.85
1.00
6.79
2.03

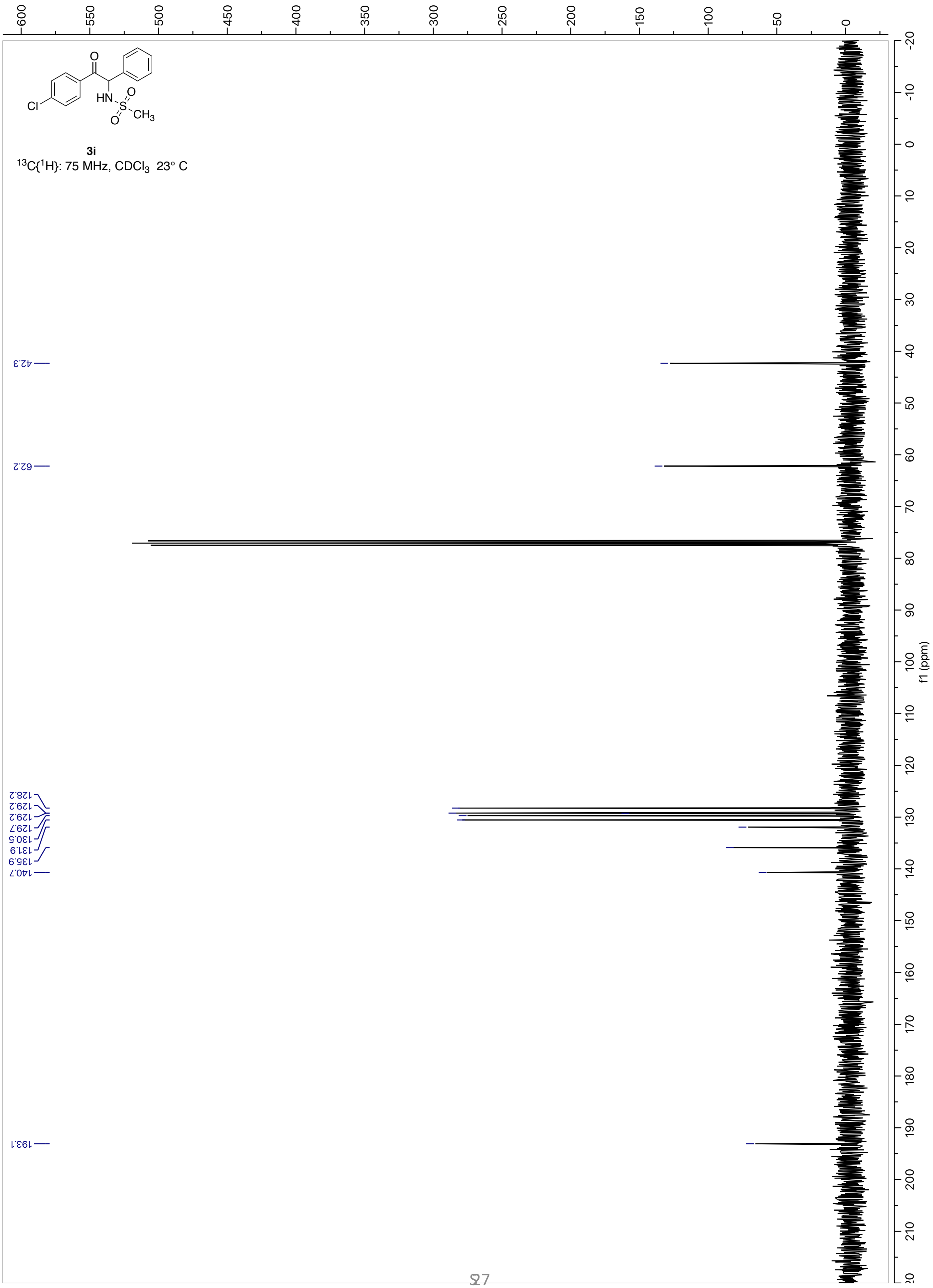
O
HN
S
O
O
CH3
Cl
3i
13C{1H}: 75 MHz, CDCl3 23° C
42.3
62.2
128.2
129.2
129.2
129.7
130.5
131.9
135.9
140.7
193.1
f1 (ppm)

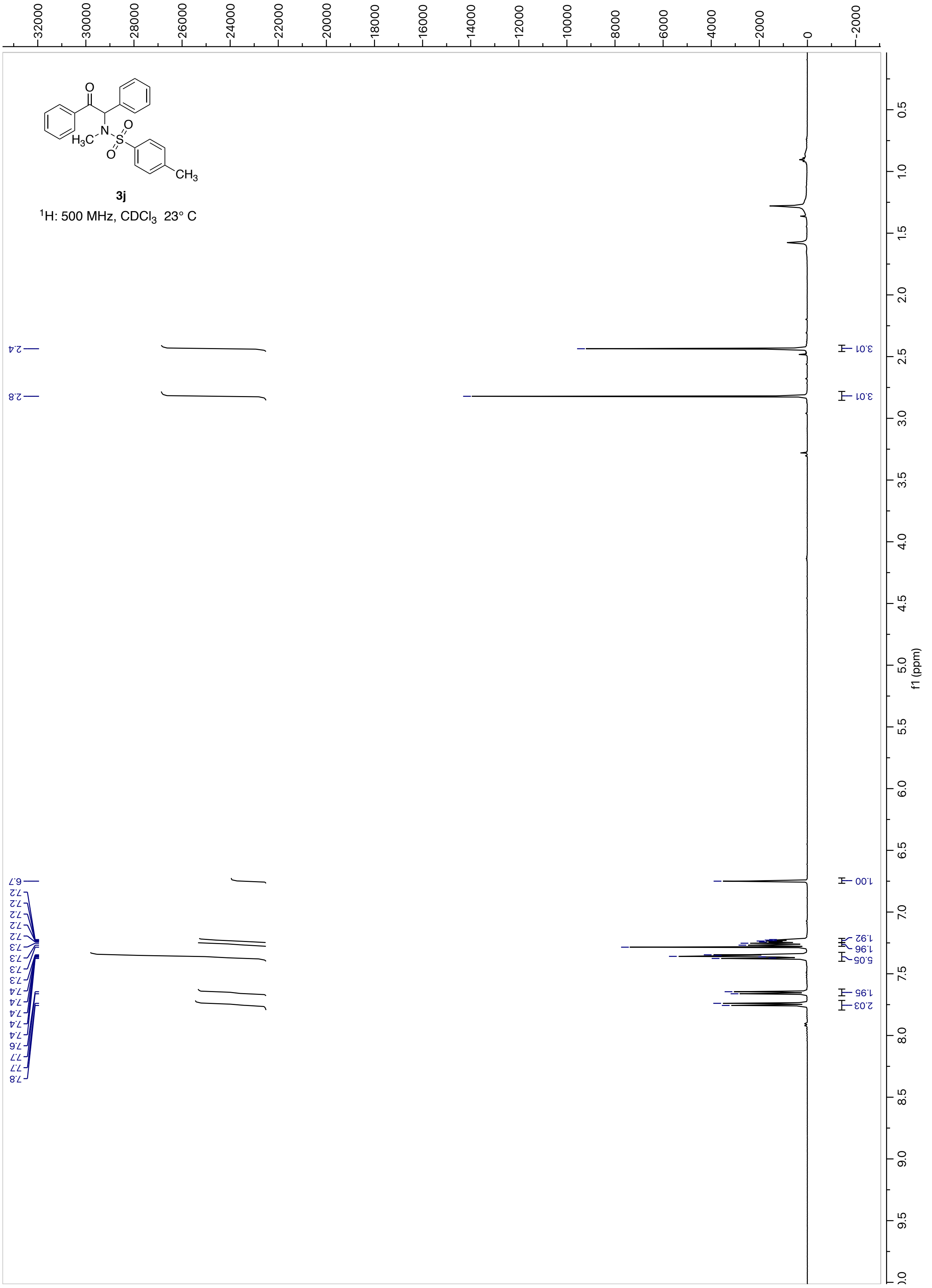
3j
^{1}H: 500 MHz, CDCl$_3$ 23° C
f1 (ppm)

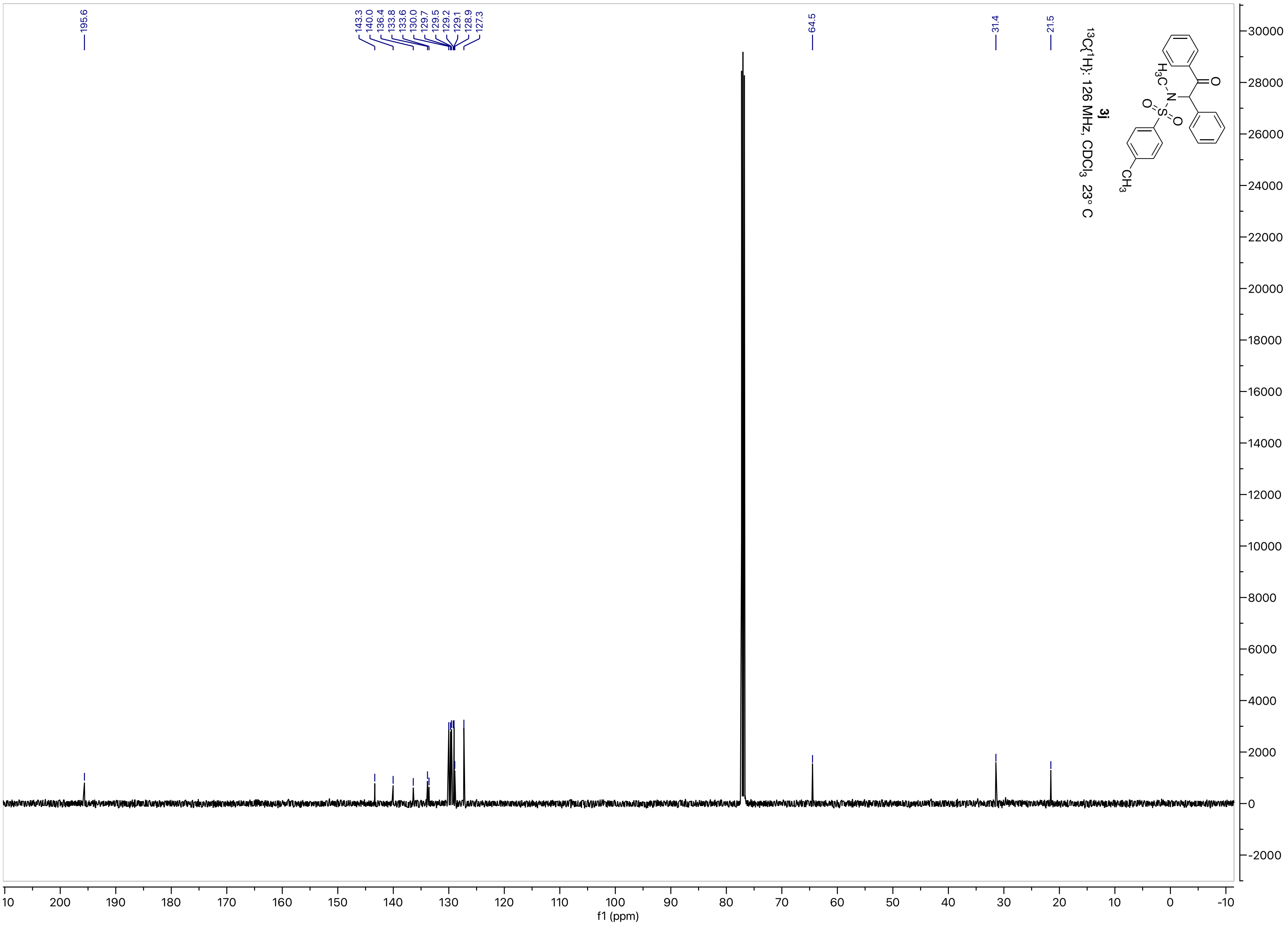
3j
$^{13}C\{^1H\}$: 126 MHz, $CDCl_3$ 23° C
195.6
143.3
140.0
136.4
133.8
133.6
130.0
129.7
129.5
129.2
129.1
128.9
127.3
64.5
31.4
21.5
f1 (ppm)

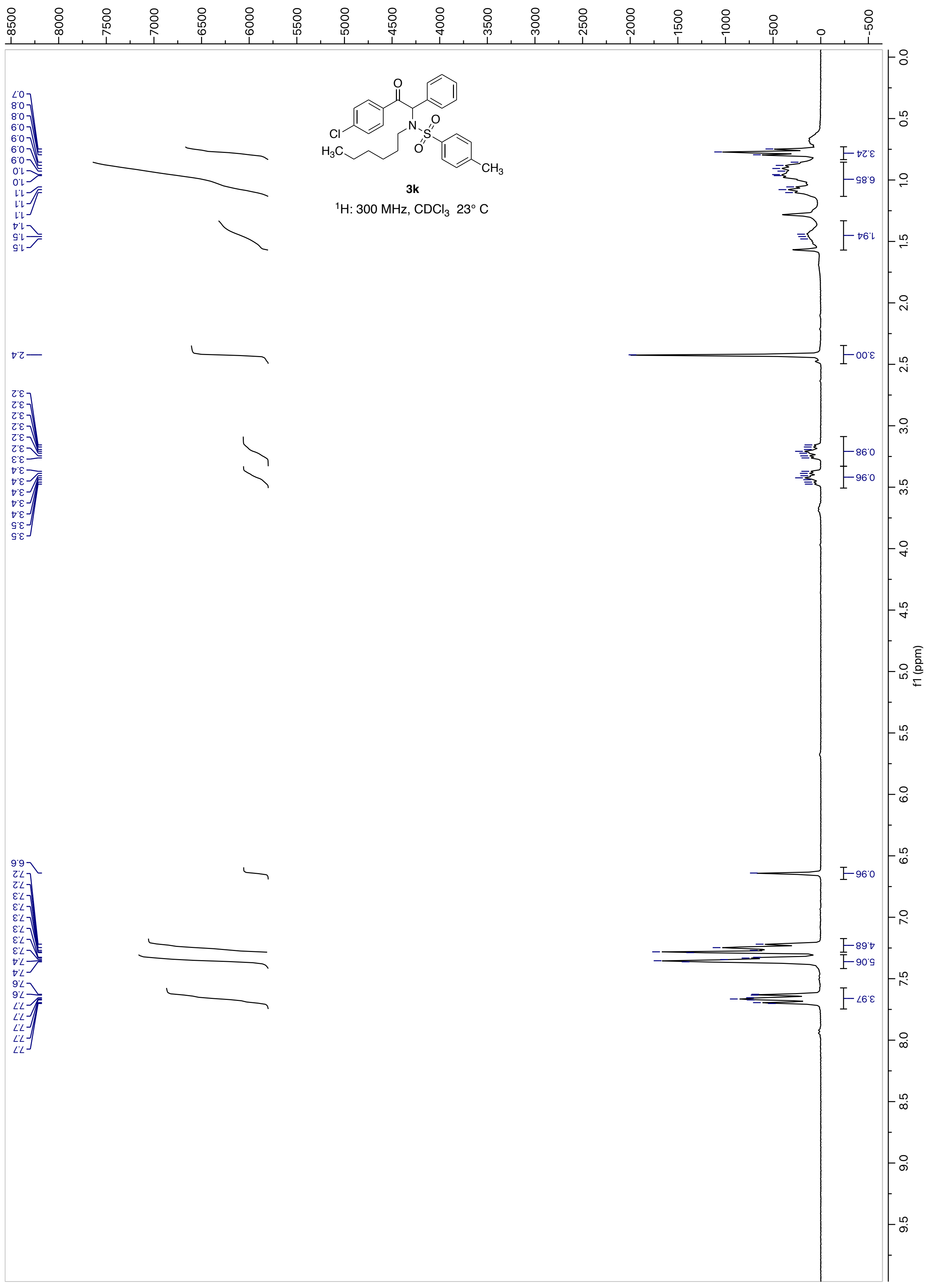
3k
1H: 300 MHz, CDCl3 23° C
f1 (ppm)

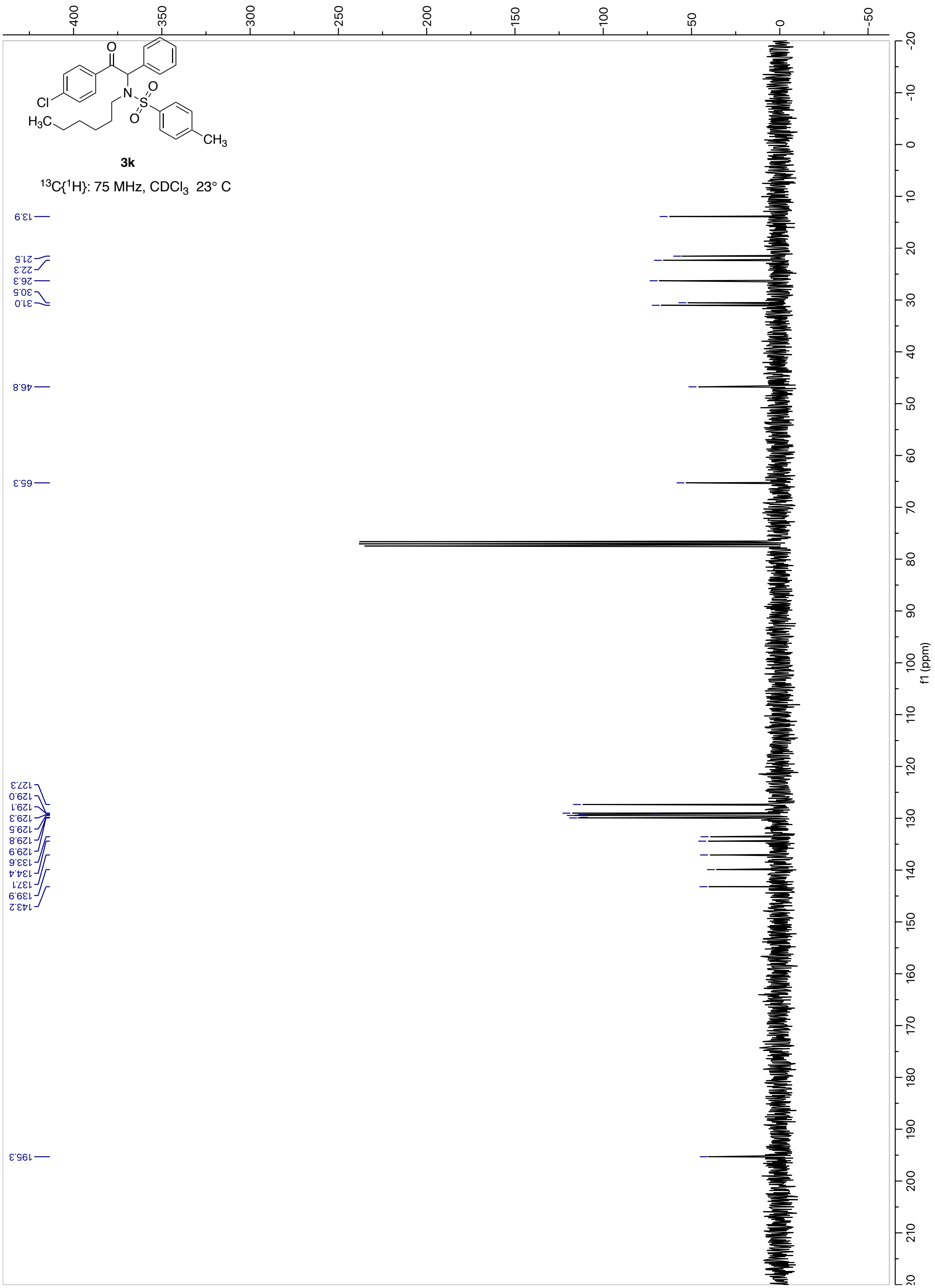
Cl
H3C
CH3
3k
13C{1H}: 75 MHz, CDCl3 23° C
f1 (ppm)

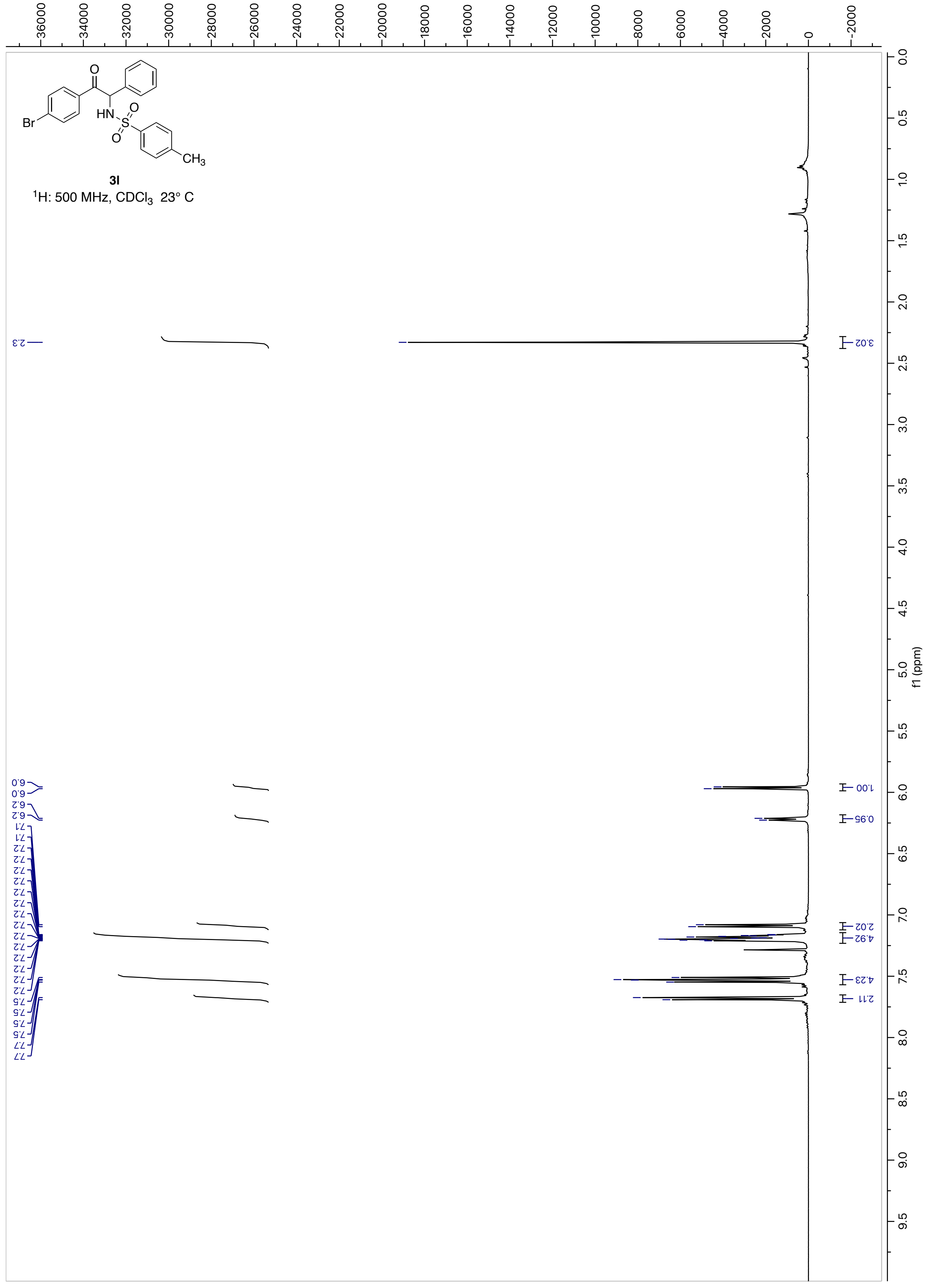

Br
HN
CH3
3l
1H: 500 MHz, CDCl3 23° C
f1 (ppm)

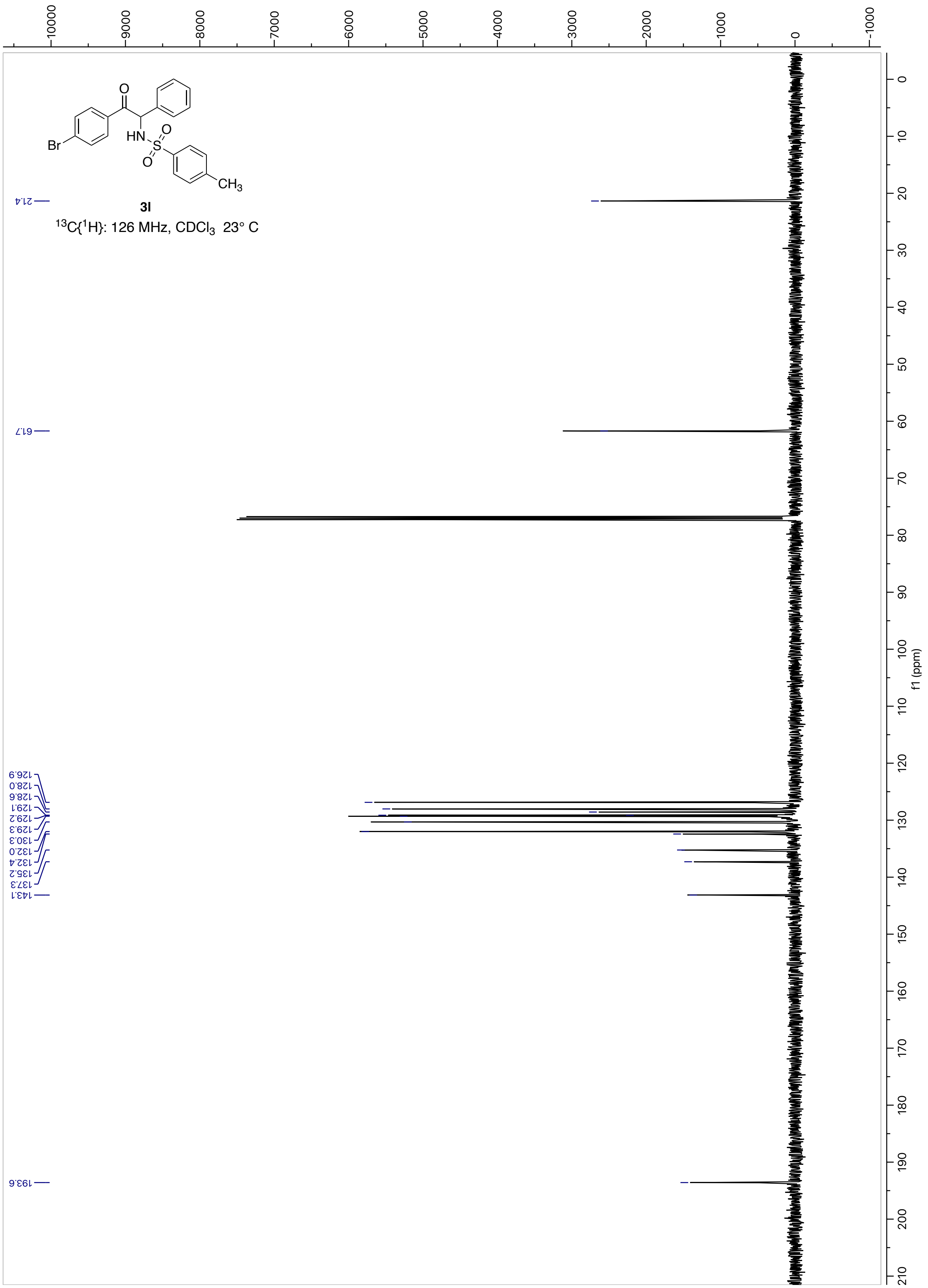
3l
$^{13}C\{^1H\}$: 126 MHz, $CDCl_3$ 23° C
21.4
61.7
126.9
128.0
128.6
129.1
129.2
129.3
130.3
132.0
132.4
135.2
137.3
143.1
193.6
f1 (ppm)

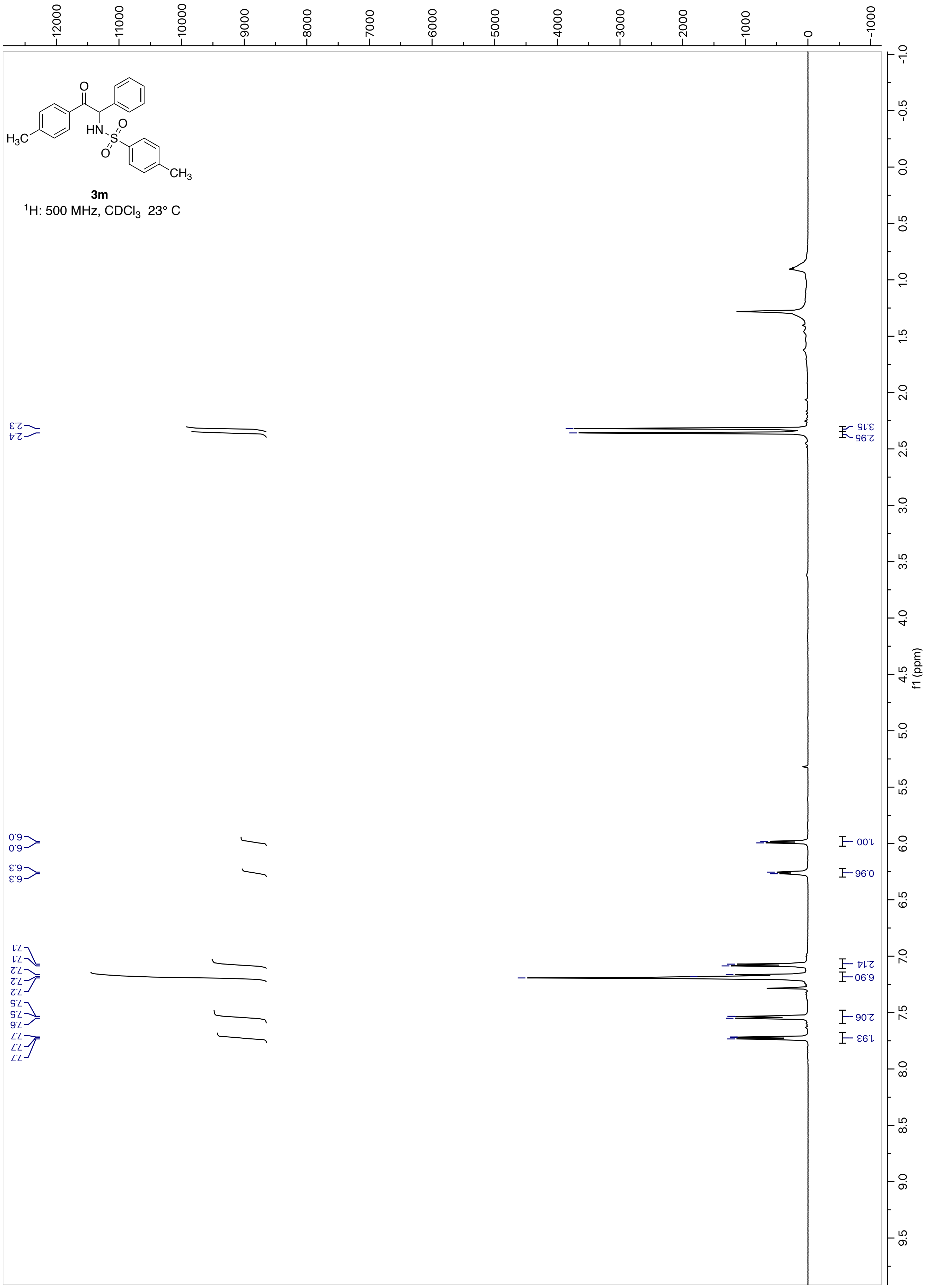
3m
1H: 500 MHz, CDCl3 23° C
f1 (ppm)

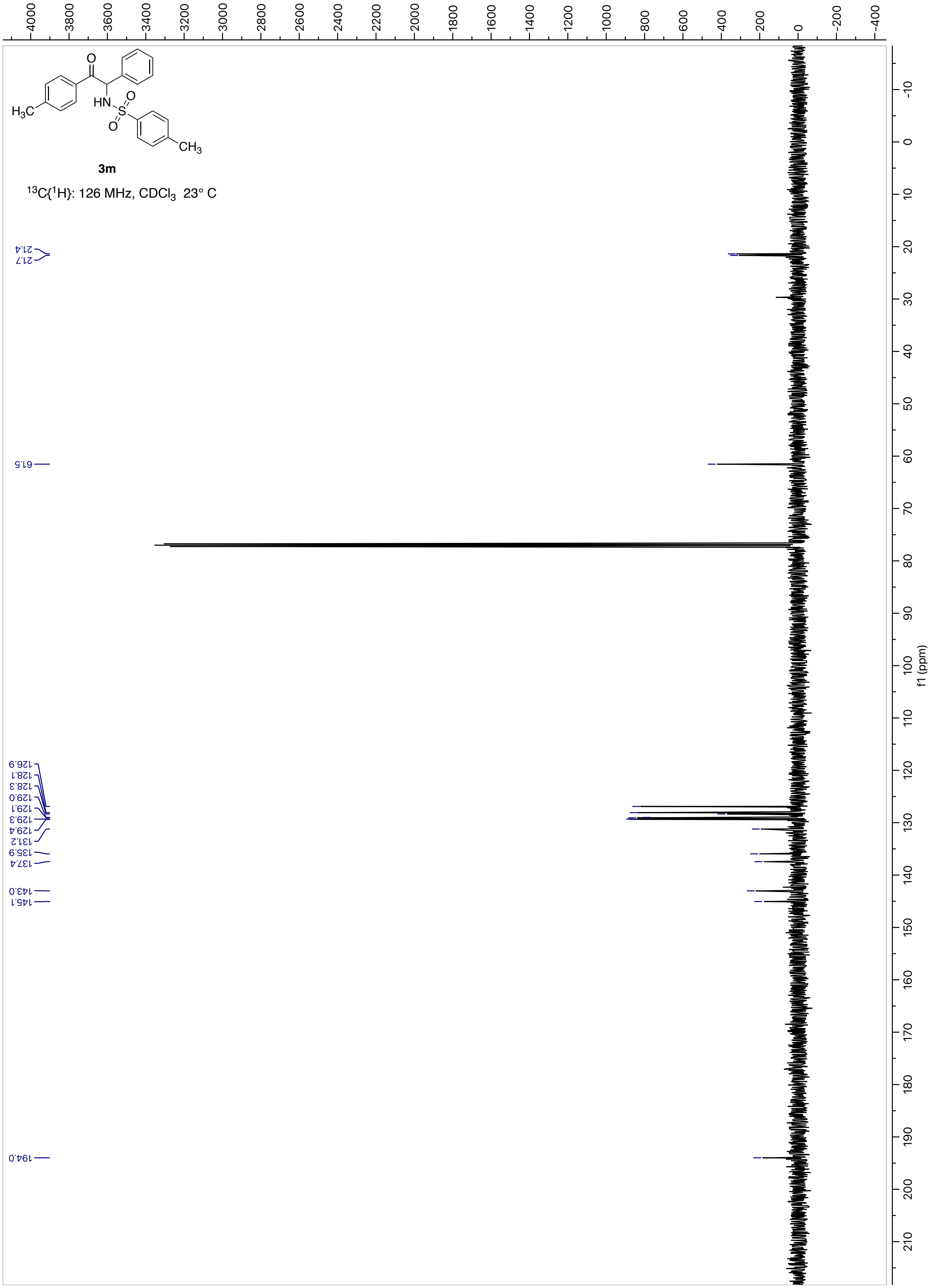
3m
$^{13}C\{^1H\}$: 126 MHz, $CDCl_3$ 23° C
21.4
21.7
61.5
126.9
128.1
128.3
129.0
129.1
129.3
129.4
131.2
135.9
137.4
143.0
145.1
194.0
f1 (ppm)

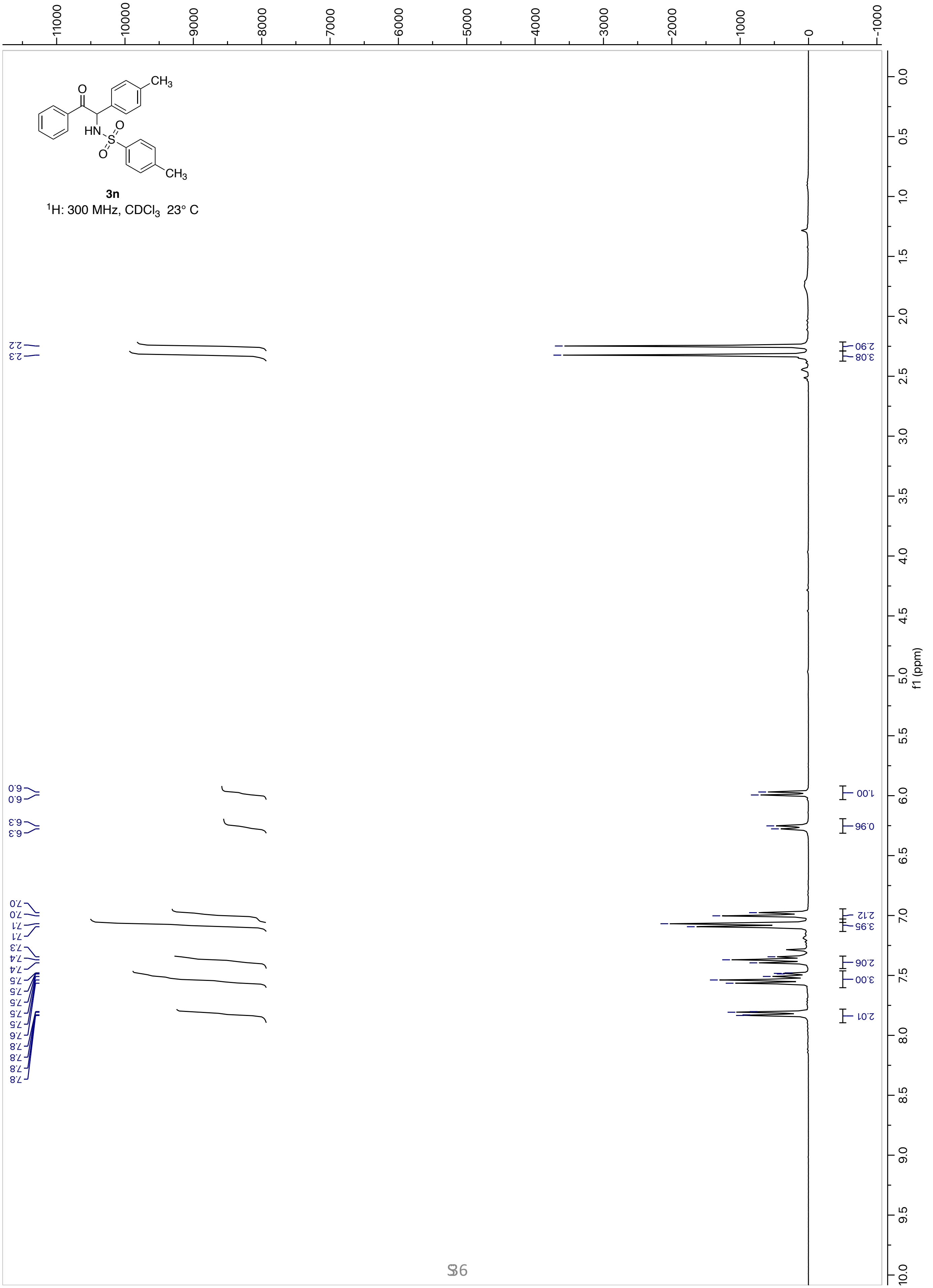
CH3
HN
S
O
O
O
CH3
3n
1H: 300 MHz, CDCl3 23° C
f1 (ppm)

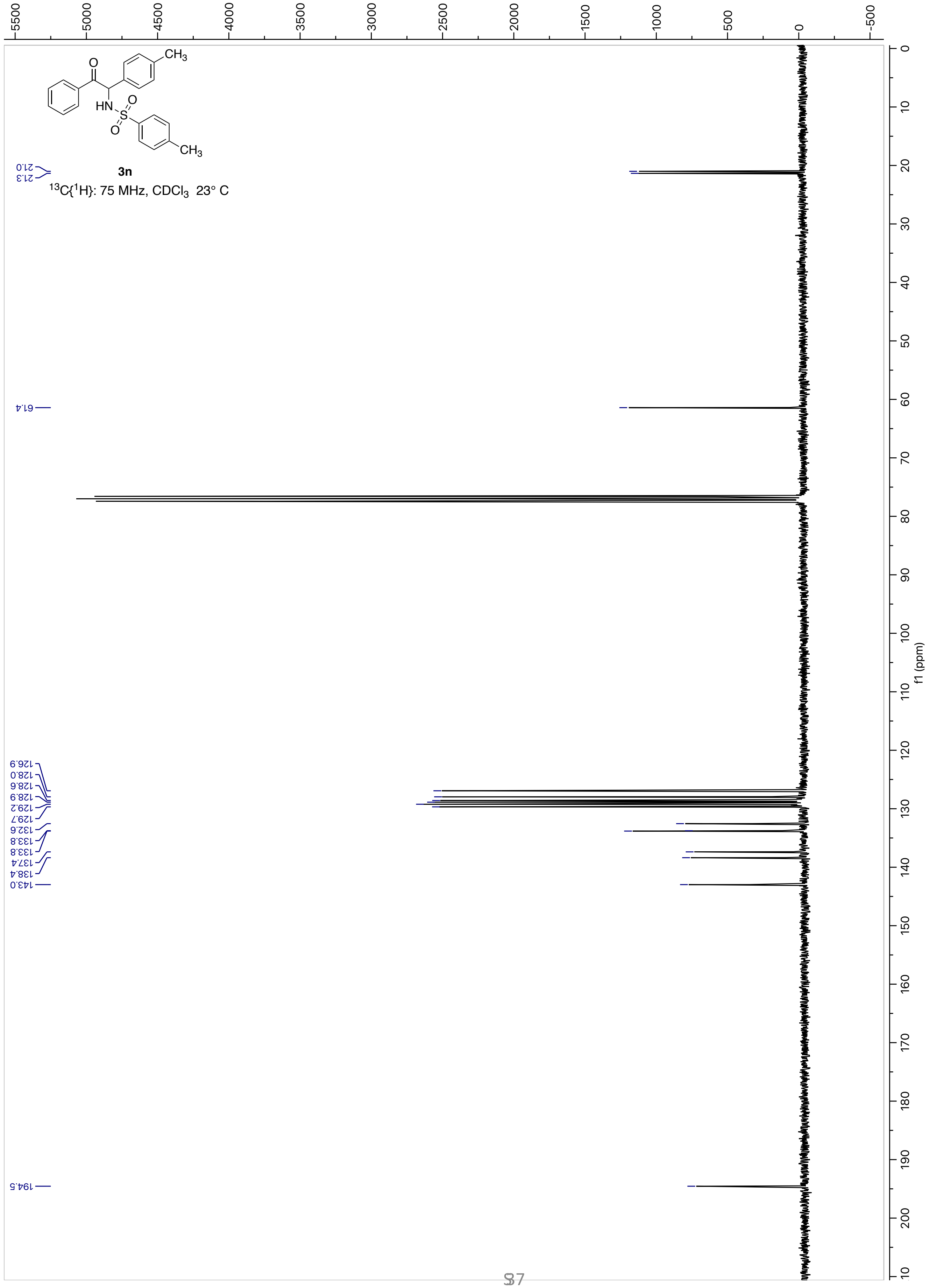
3n
$^{13}C\{^1H\}$: 75 MHz, $CDCl_3$ 23° C
21.0
21.3
61.4
126.9
128.0
128.6
128.9
129.2
129.7
132.6
133.8
133.8
137.4
138.4
143.0
194.5
f1 (ppm)

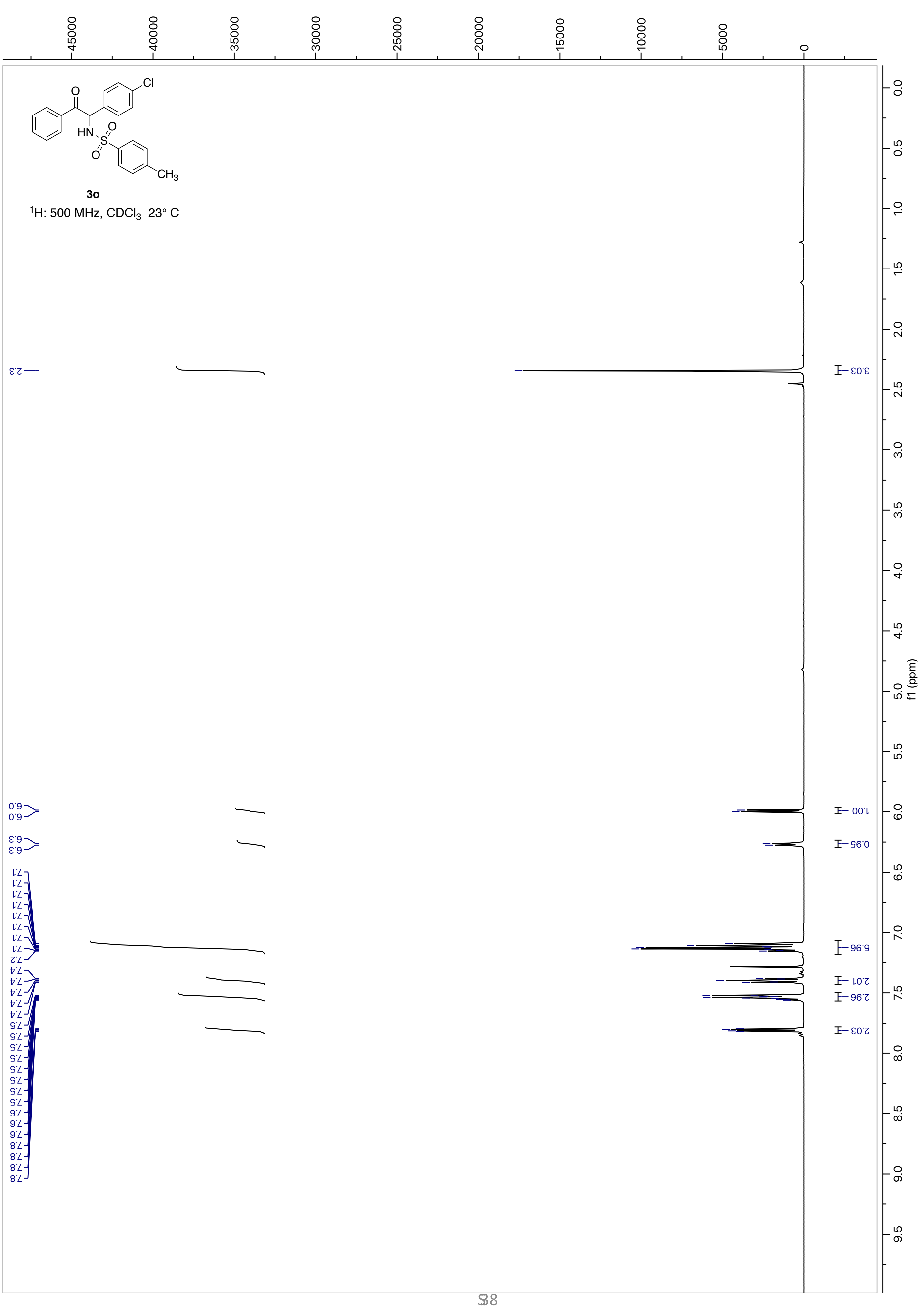
3o
¹H: 500 MHz, CDCl₃ 23° C
f1 (ppm)

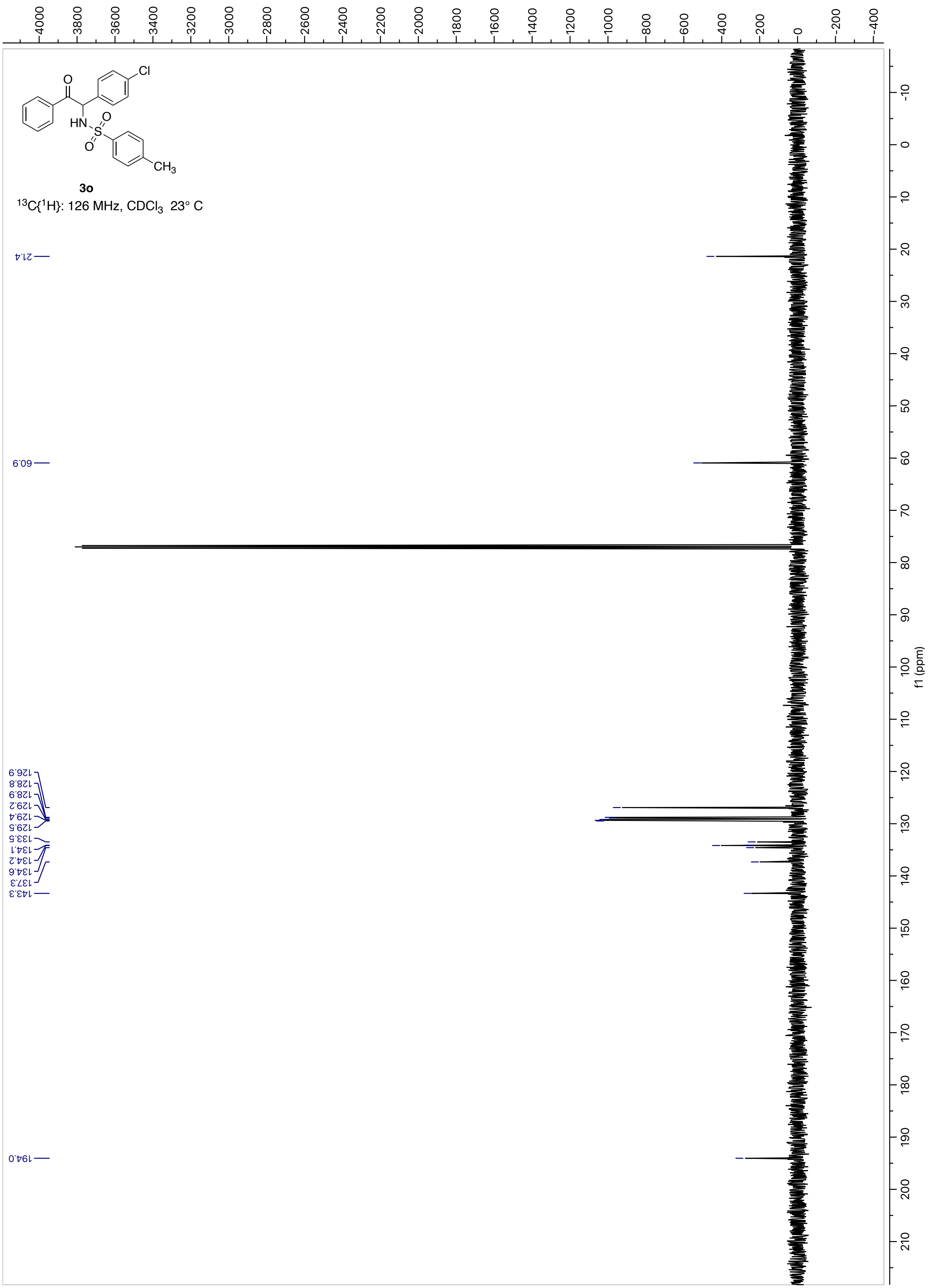
Cl
O
HN
S
O
O
CH3
3o
13C{1H}: 126 MHz, CDCl3 23° C
21.4
60.9
126.9
128.8
128.9
129.2
129.4
129.5
133.5
134.1
134.2
134.6
137.3
143.3
194.0
f1 (ppm)

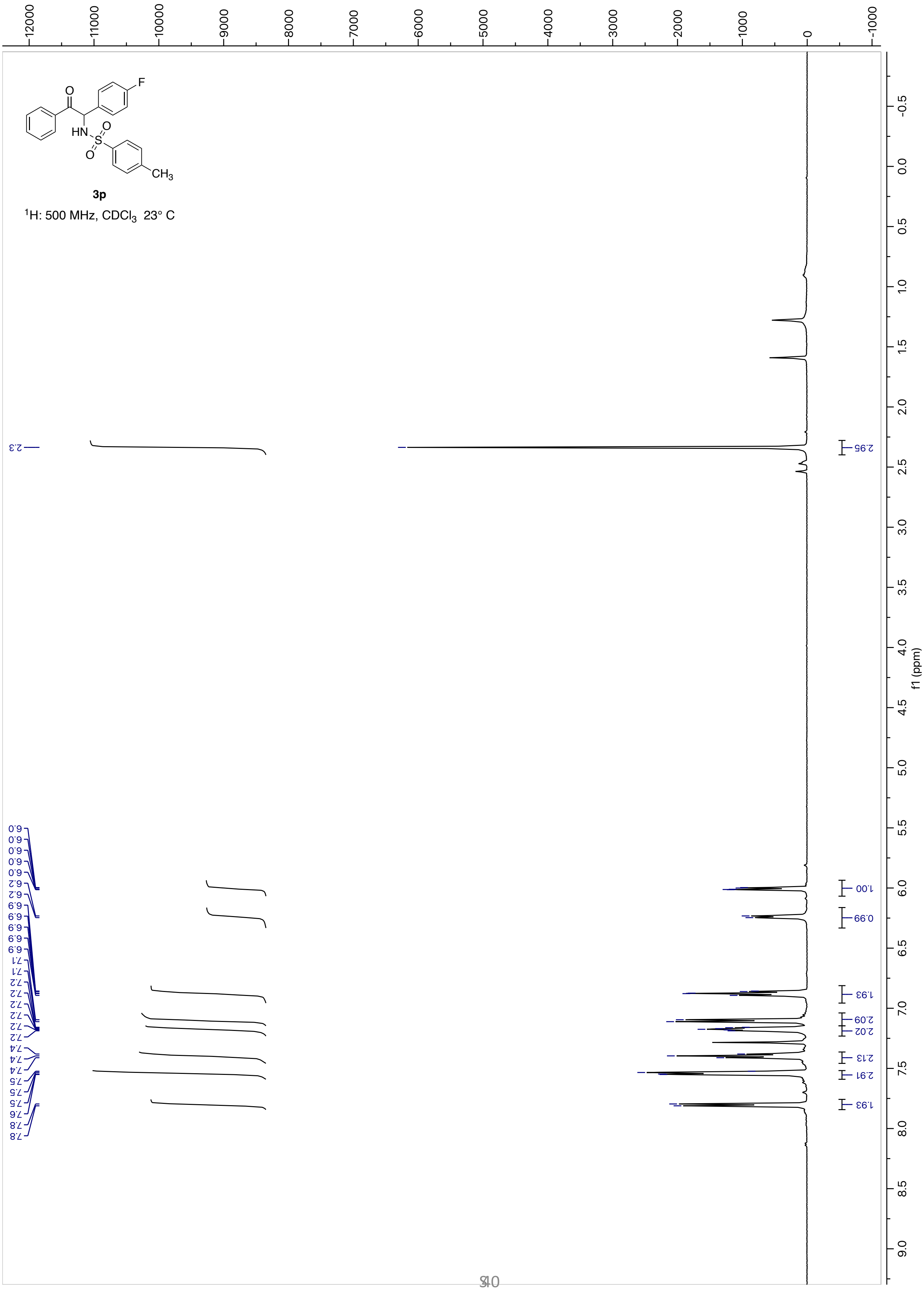
3p
1H: 500 MHz, CDCl3 23° C
f1 (ppm)

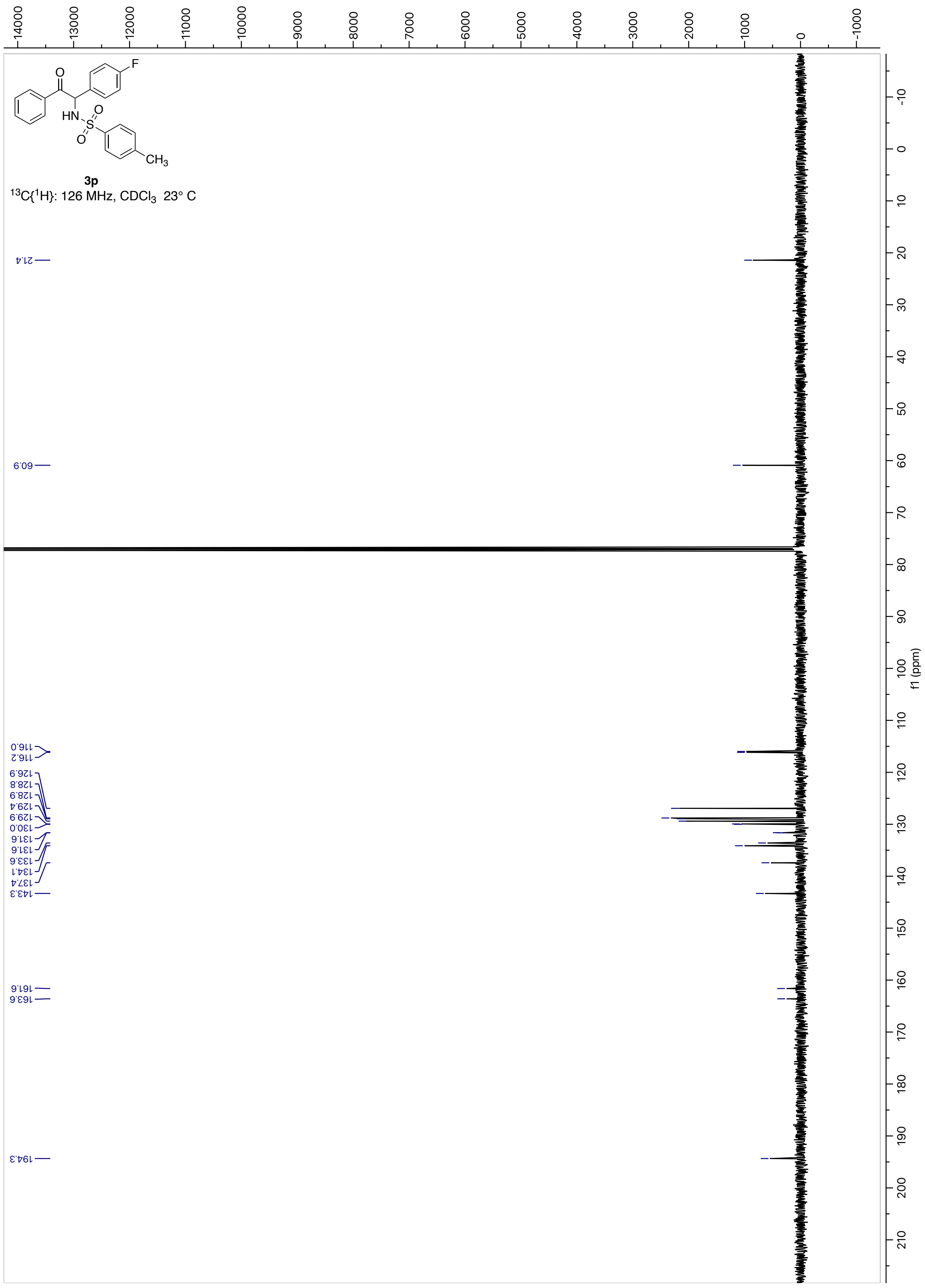
3p
$^{13}C\{^1H\}$: 126 MHz, $CDCl_3$ 23° C
HN
O
S
F
CH_3
194.3
163.6
161.6
143.3
137.4
134.1
133.6
131.6
131.6
130.0
129.9
129.4
128.9
128.8
126.9
116.2
116.0
60.9
21.4
f1 (ppm)
210
200
190
180
170
160
150
140
130
120
110
100
90
80
70
60
50
40
30
20
10
0
-10
14000
13000
12000
11000
10000
9000
8000
7000
6000
5000
4000
3000
2000
1000
0
-1000

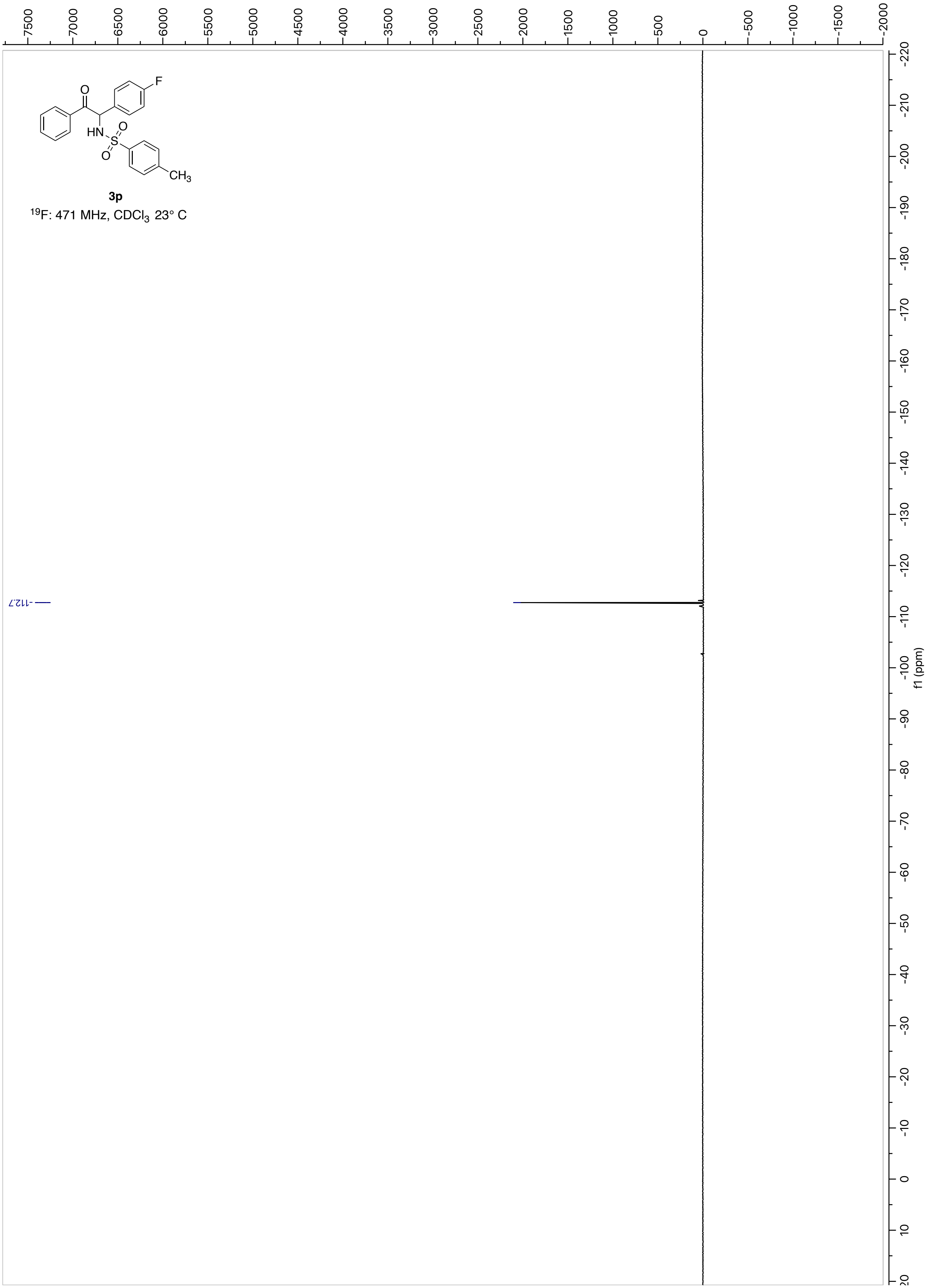

O
F
HN
S
O
O
CH3
3p
19F: 471 MHz, CDCl3 23° C
-112.7
f1 (ppm)

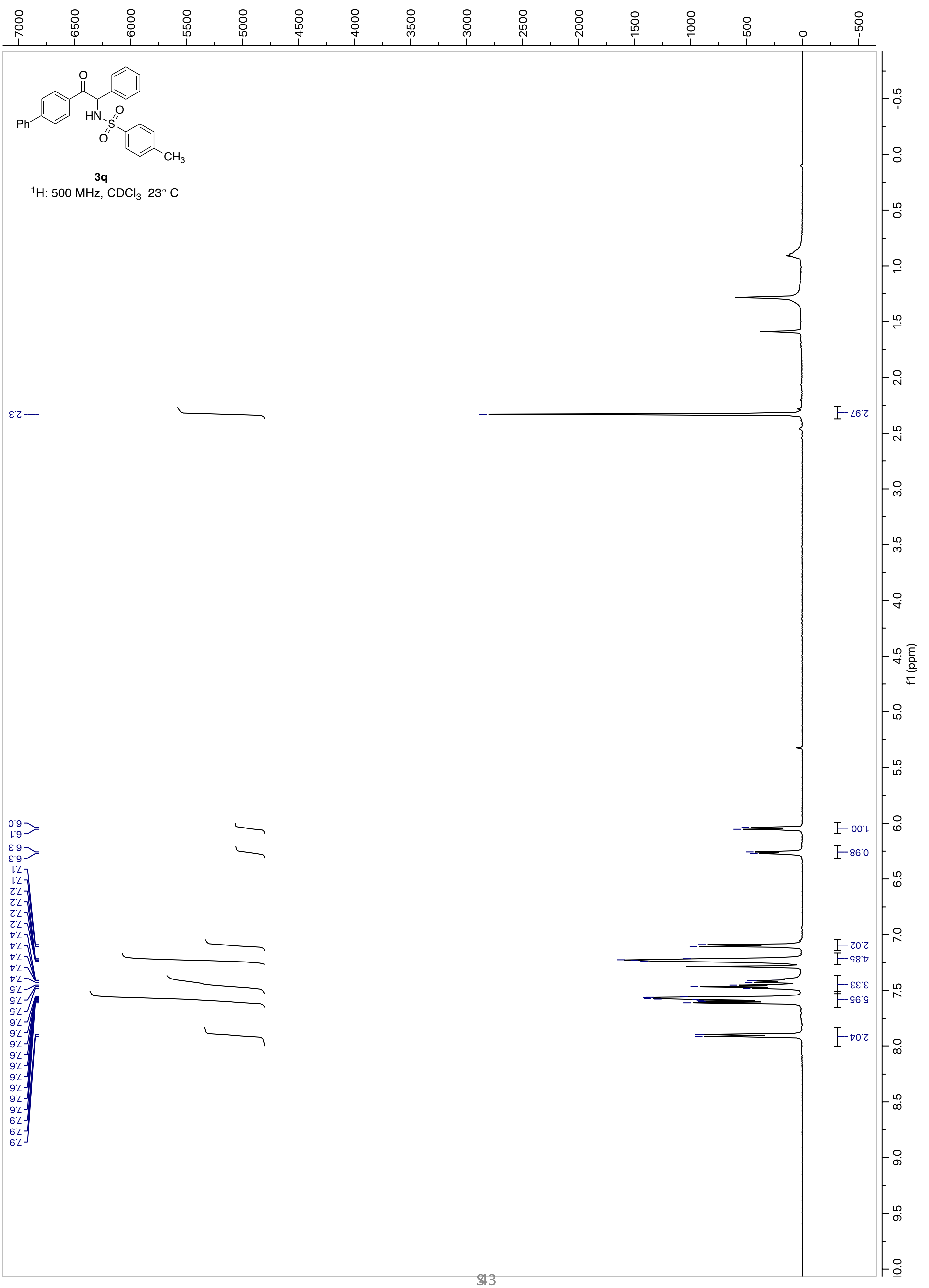
Ph
HN
O
S
CH3
3q
1H: 500 MHz, CDCl3 23° C
f1 (ppm)

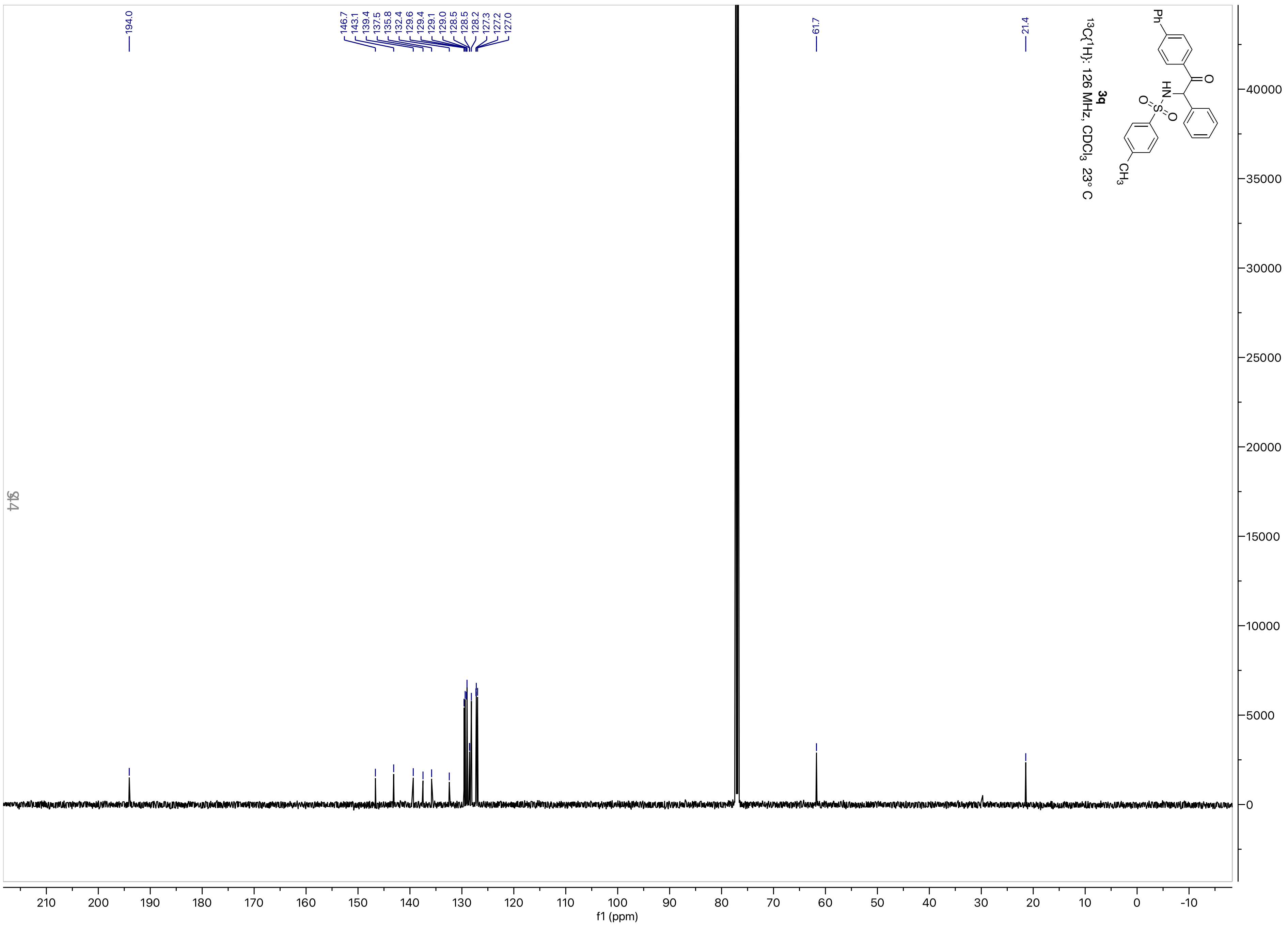
Ph
O
HN
S
O
O
CH3
3q
13C{1H}: 126 MHz, CDCl3 23° C
194.0
146.7
143.1
139.4
137.5
135.8
132.4
129.6
129.4
129.1
129.0
128.5
128.5
128.2
127.3
127.2
127.0
61.7
21.4
f1 (ppm)

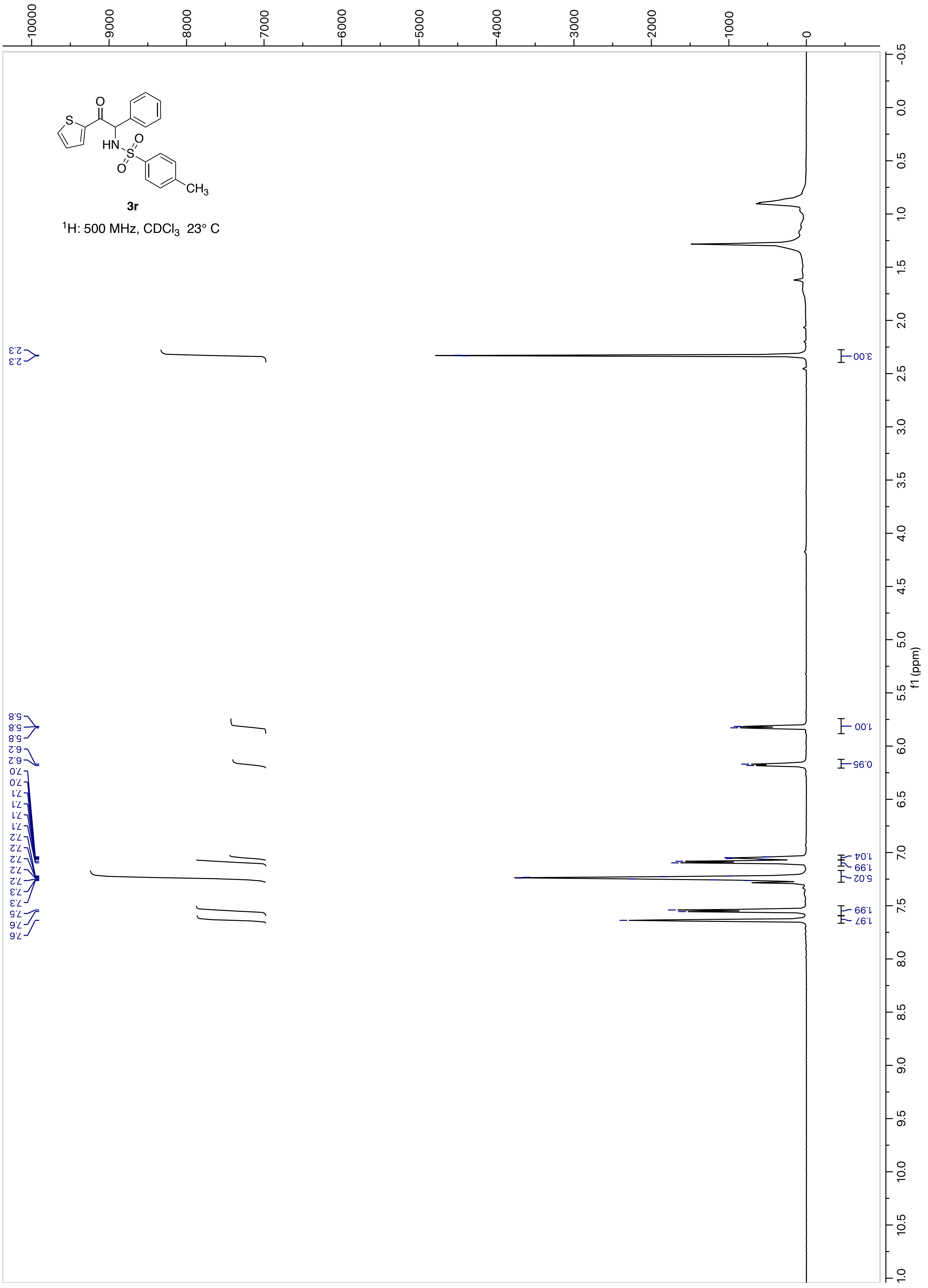
3r
^{1}H: 500 MHz, CDCl$_3$ 23° C
f1 (ppm)

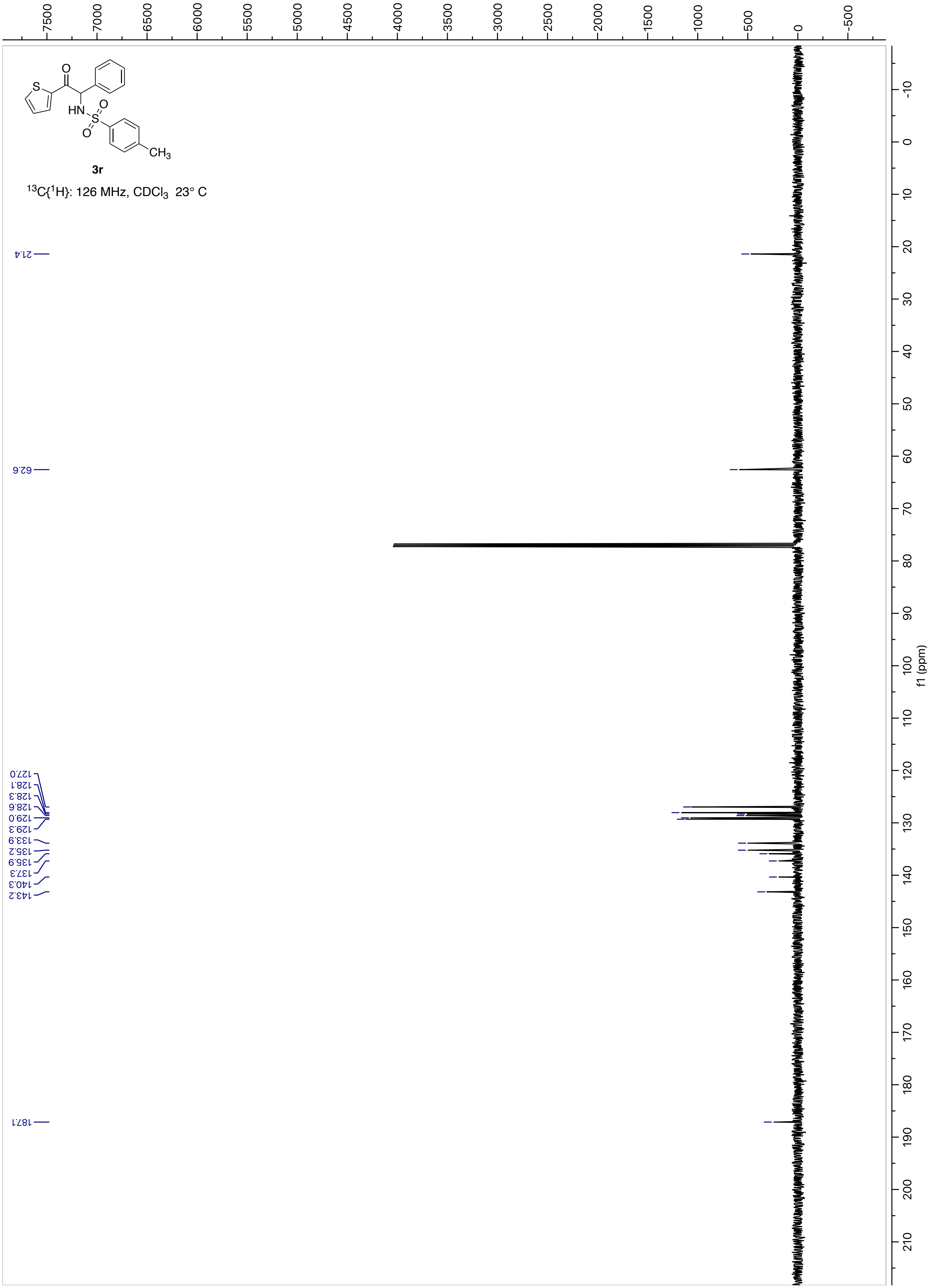
3r
$^{13}C\{^{1}H\}$: 126 MHz, $CDCl_3$ 23° C
21.4
62.6
127.0
128.1
128.3
128.6
129.0
129.3
133.9
135.2
135.9
137.3
140.3
143.2
187.1
f1 (ppm)

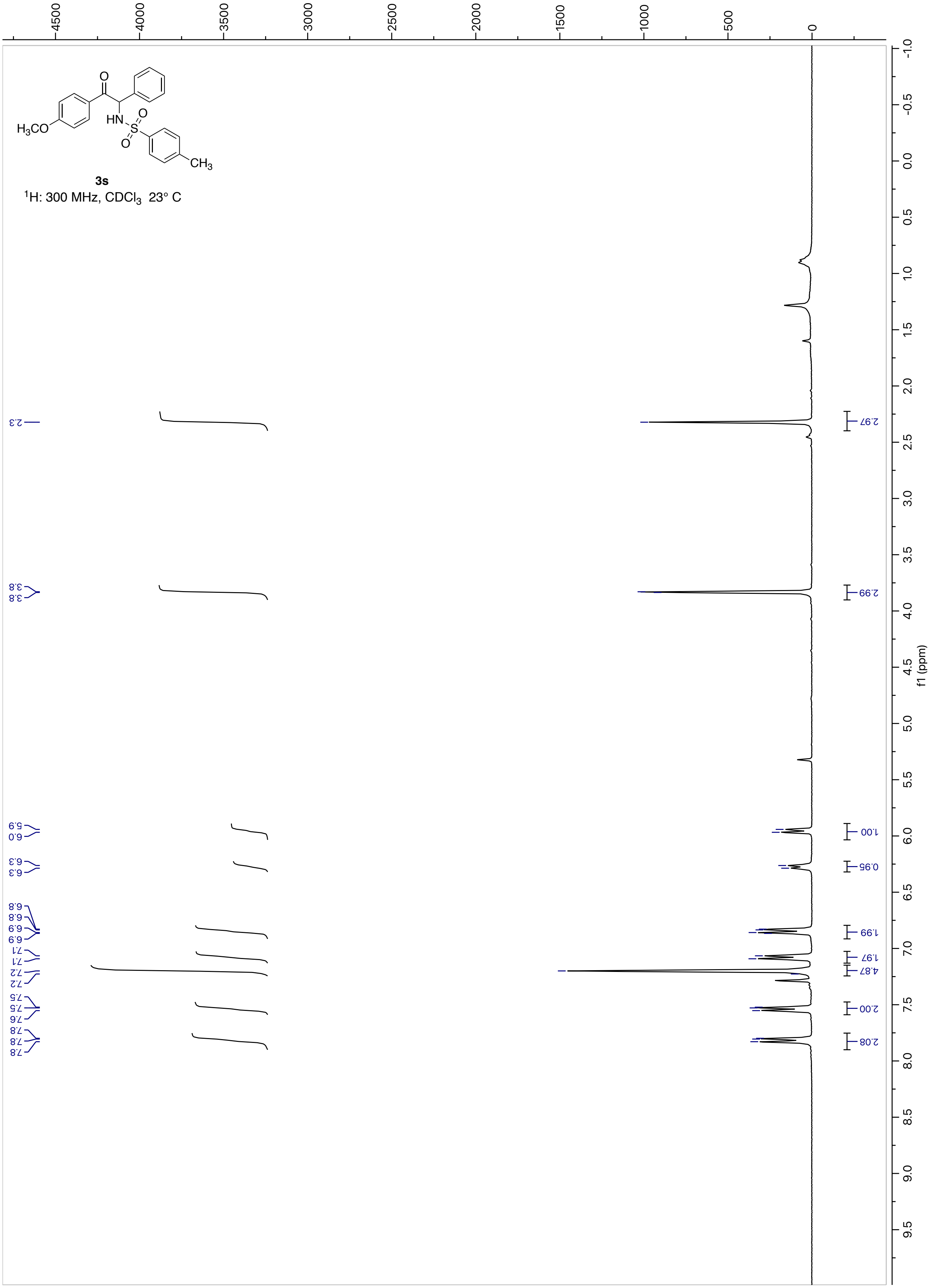
3s
^{1}H: 300 MHz, $CDCl_3$ 23° C
f1 (ppm)
2.97
2.99
1.00
0.95
1.99
1.97
4.87
2.00
2.08
2.3
3.8
3.8
5.9
6.0
6.3
6.3
6.8
6.8
6.9
6.9
7.1
7.1
7.2
7.2
7.5
7.5
7.6
7.8
7.8
7.8

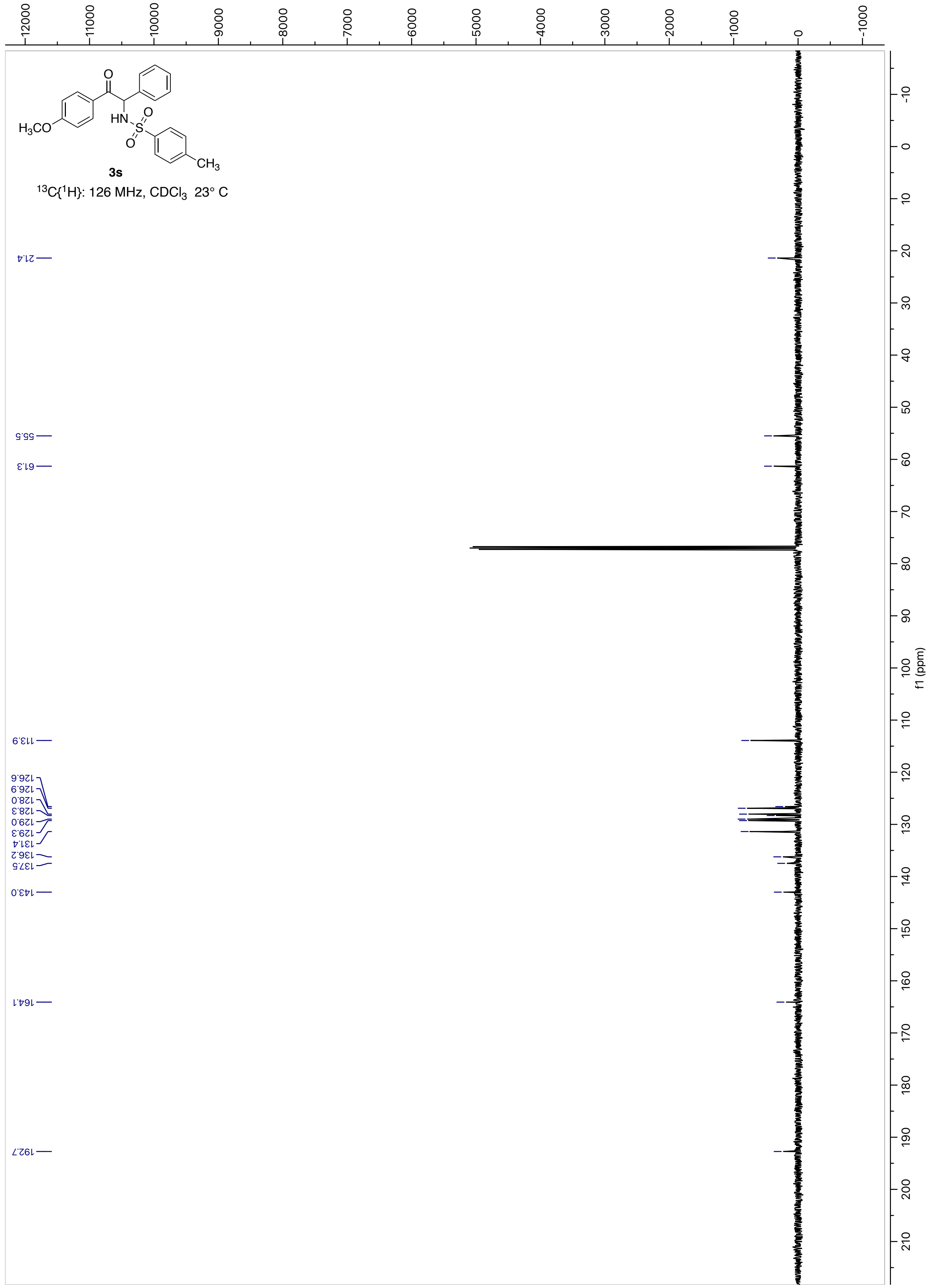
3s
$^{13}C\{^1H\}$: 126 MHz, $CDCl_3$ 23° C
21.4
55.5
61.3
113.9
126.6
126.9
128.0
128.3
129.0
129.3
131.4
136.2
137.5
143.0
164.1
192.7
f1 (ppm)

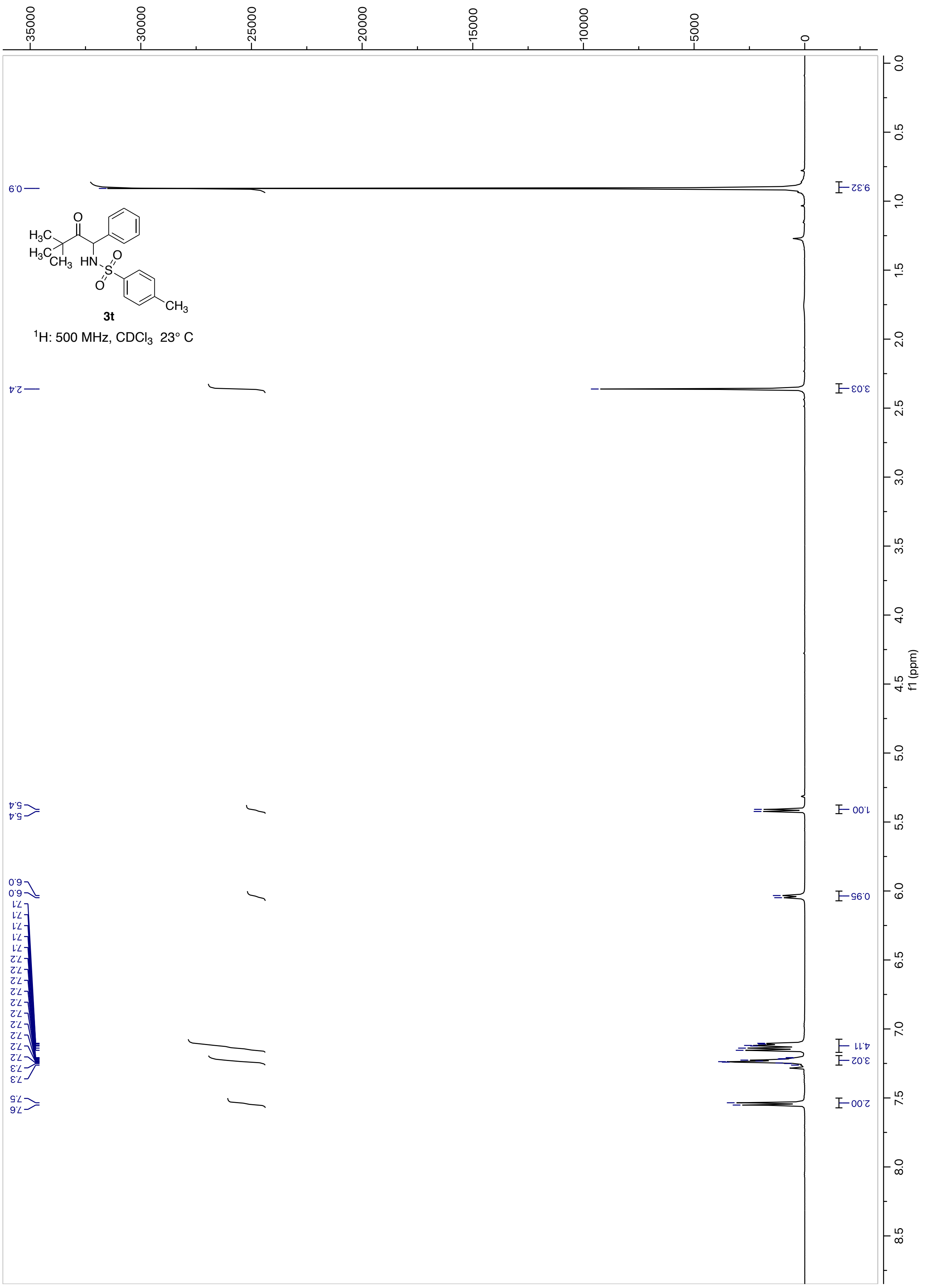

3t
1H: 500 MHz, CDCl3 23° C
f1 (ppm)

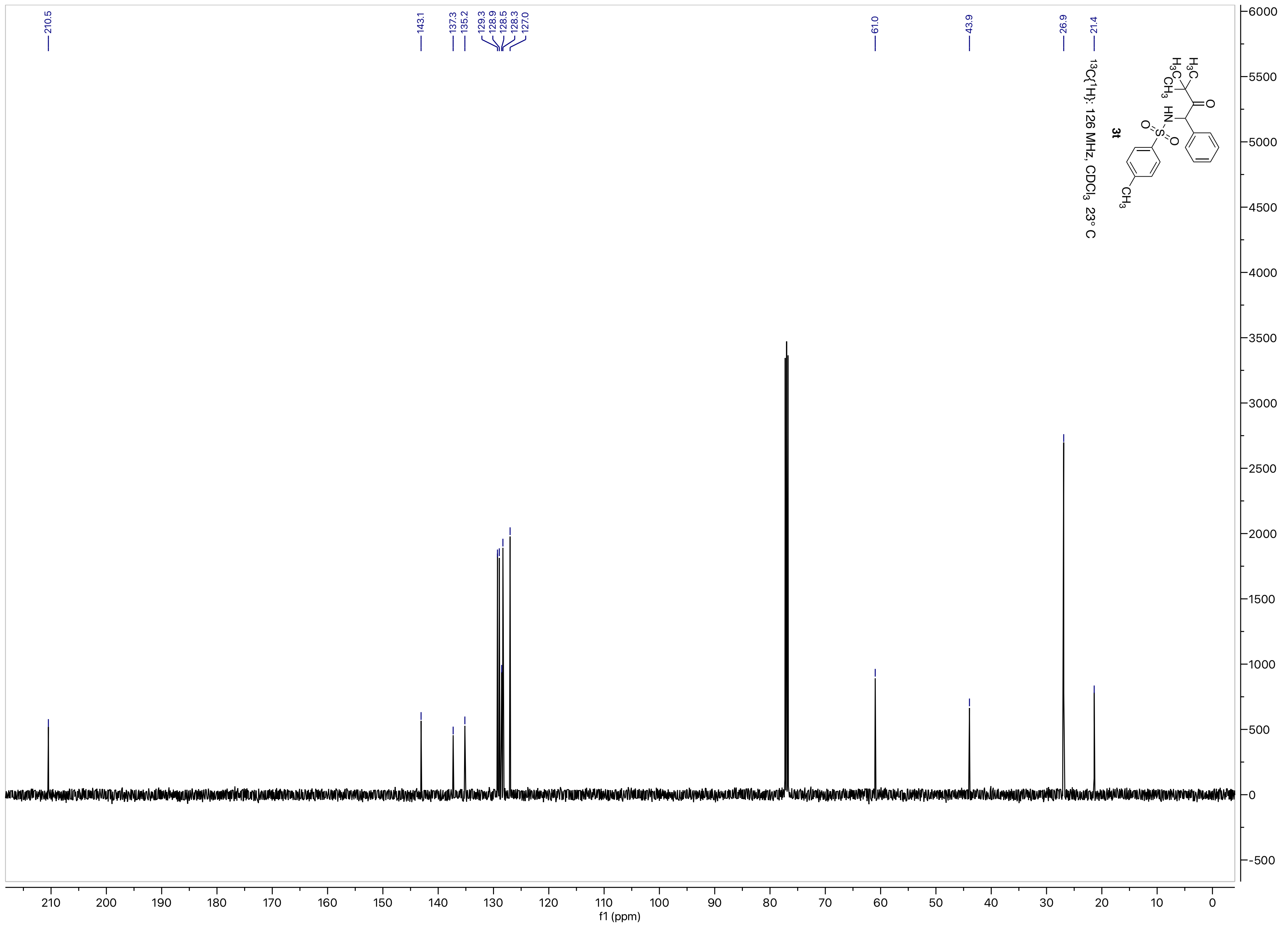
210.5
143.1
137.3
135.2
129.3
128.9
128.5
128.3
127.0
61.0
43.9
26.9
21.4
3t
$^{13}C\{^{1}H\}$: 126 MHz, $CDCl_3$ 23° C
f1 (ppm)

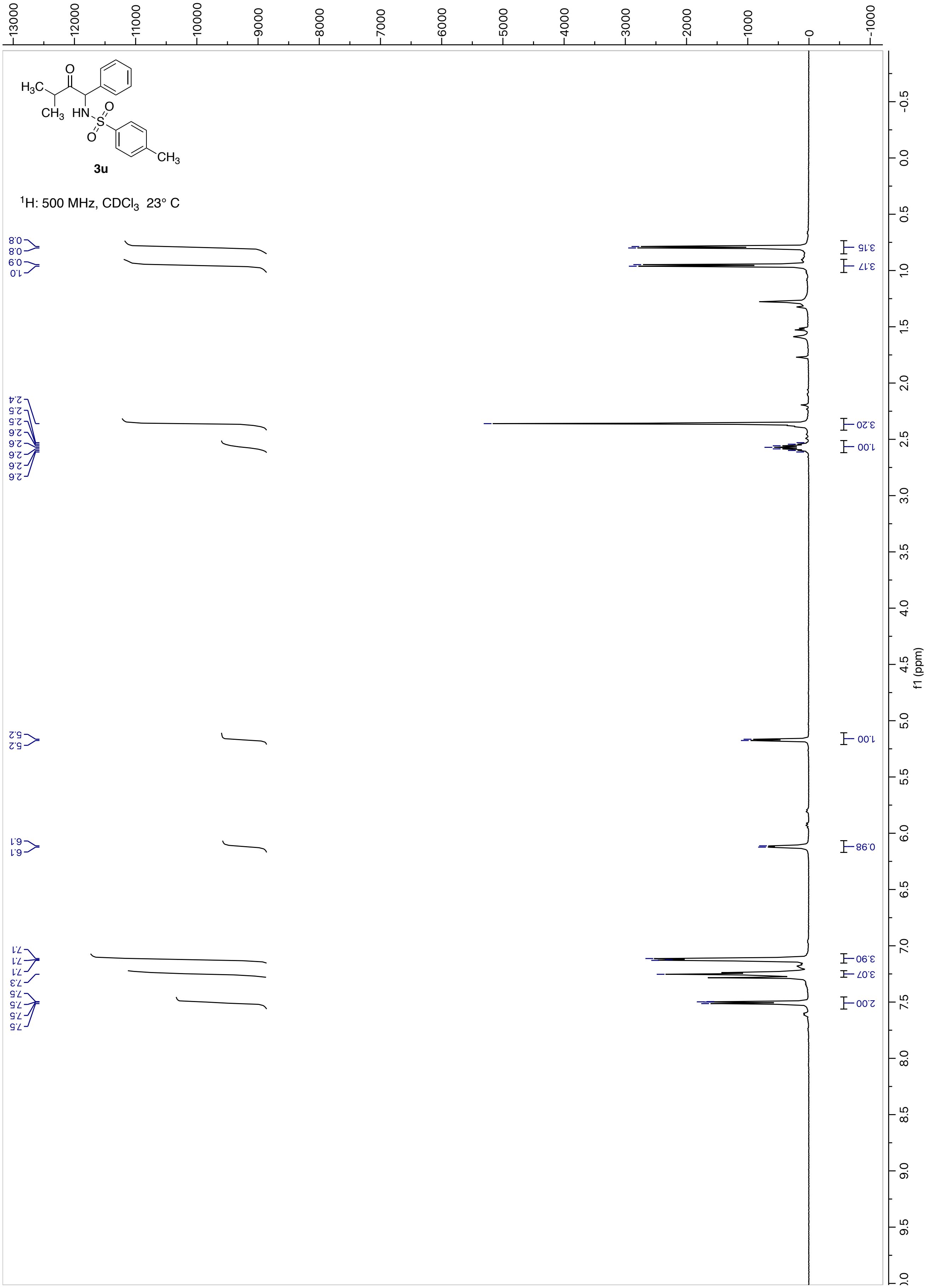
3u
^{1}H: 500 MHz, $CDCl_3$ 23° C
f1 (ppm)

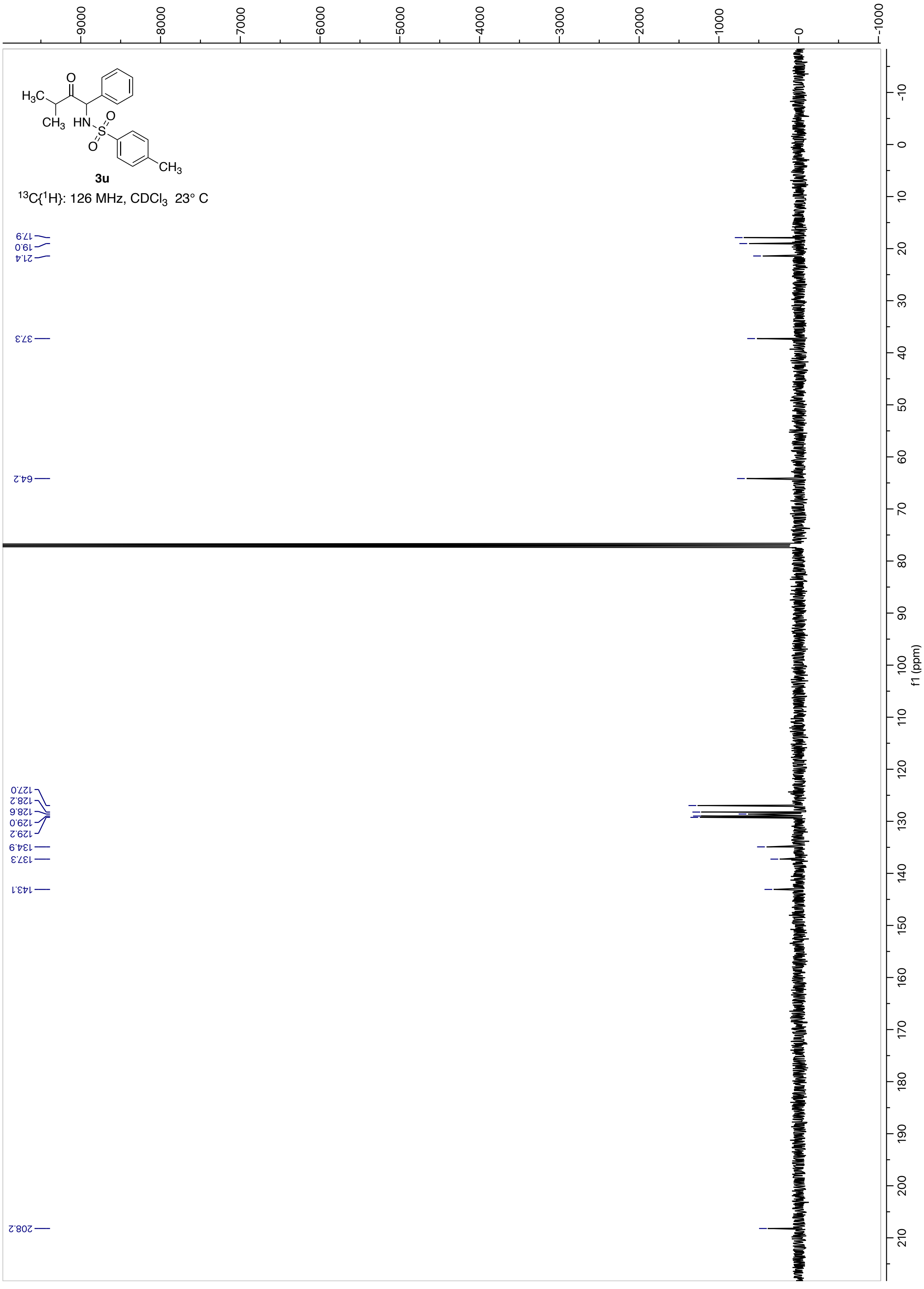
3u
13C{1H}: 126 MHz, CDCl3 23° C
17.9
19.0
21.4
37.3
64.2
127.0
128.2
128.6
129.0
129.2
134.9
137.3
143.1
208.2
f1 (ppm)
